# Supplementary material for: Systematic review and tools appraisal of prognostic factors of return to work in workers on sick leave due to musculoskeletal and common mental disorders
Source: PLoS One. 2024 Jul 17;19(7):e0307284. doi: 10.1371/journal.pone.0307284 (PMC11253986; doi:10.1371/journal.pone.0307284)
Supplement: S1 File — (DOCX) [file pone.0307284.s001.docx]

**Supplementary material 1**

**Operationalization of psychometric and usability criteria**

PSYCHOMETRIC CRITERIA. **Face validity**, which is inherently satisfied for physical examination tools, was more formally assessed with questionnaires by evaluating whether their items refer to the specific factor/concept of interest. Team members (co-authors) have the complementary expertise to perform this assessment. Actually, before determining the levels of evidence of each factor, some tools (often single-item tools) that were interpreted (in the article) as measuring a given factor (e.g., recovery expectations) were in fact measuring another concept (e.g., RTW expectations) and consequently, were ascribed to the right concept (here RTW expectations). Consequently, this close inspection of the tools always ensured that face validity was met. **Construct validity** was evaluated positively if a factor analysis of the structure of the measure was conducted. **Convergent validity** was evaluated by significant and positive correlations (r≥0.30) with theoretically similar concepts. **Internal consistency** was evaluated positively with ratings for Cronbach’s alpha between .70 and .95. **Test-retest reliability** was rated positively when repeated testing of the same participants yielded reproducible results (correlation coefficients higher than .60). **Predictive validity** was automatically satisfied because only those tools that demonstrated a significant correlation with RTW were retained. From the information available (initial validation study or review on psychometric properties), when a given psychometric property was not documented, it was considered untested and not met.

USABILITY CRITERIA (PRACTICAL RELEVANCE). **Time for completion** of the measure was rated as favourable for tools taking ≤ 5 minutes to complete or having less than 8 items in case of questionnaires. **Administrative burden** was assessed as favourably when items (or procedures in case of clinical examination) and response options (or observations) are easy, and the final score was calculated simply. For questionnaires, scoring is facilitated when adding up the item individual scores, instead of complex formula, and when there is no reversed items. **Ease of interpretation** is rated positively when not requiring specific training for administration and interpretation of the results. **Accessibility** was rated positively when the tool was available for free (in an article or online) or required simple/inexpensive equipment usually available in clinics.

**Syntax strategy**

**CINAHL DATABASE**

| S15 | S11 AND S12 AND S13 |
| --- | --- |
| S14 | S11 AND S12 AND S13 |
| S13 | S5 OR S6 OR S7 |
| S12 | S8 OR S9 OR S10 |
| S11 | S1 OR S2 OR S3 OR S4 |
| S10 | AB absence OR absenteeism OR disability OR "off work" OR off-work OR sick OR "sick leave" OR sick-listed OR sickness OR "sickness absence" OR "work interruption" OR "work-related injur*" OR "work injur*" OR "occupational diseas*" OR "occupational injur*" OR "occupational health" |
| S9 | TI absence OR absenteeism OR disability OR "off work" OR off-work OR sick OR "sick leave" OR sick-listed OR sickness OR "sickness absence" OR "work interruption" OR "work-related injur*" OR "work injur*" OR "occupational diseas*" OR "occupational injur*" OR "occupational health" |
| S8 | (MH "Accidents, Occupational") OR (MH "Occupational-Related Injuries") OR (MH "Occupational Diseases") OR (MH "Sick Leave") OR (MH "Absenteeism") |
| S7 | AB "back to work" OR "job maintenance" OR "job reentry" OR "job re-entry" OR "job retention" OR "labor force participation" OR presenteeism OR reemployment OR "resuming work" OR "return to work" OR return-to-work OR rtw OR "time at work" OR "vocational rehabilitation" OR "work re-entry" OR "work re-entry" OR "work reintegration" OR "work re-integration" OR "job reintegration" OR "job re-integration" OR "work resumption" |
| S6 | TI "back to work" OR "job maintenance" OR "job reentry" OR "job re-entry" OR "job retention" OR "labor force participation" OR presenteeism OR reemployment OR "resuming work" OR "return to work" OR return-to-work OR rtw OR "time at work" OR "vocational rehabilitation" OR "work re-entry" OR "work re-entry" OR "work reintegration" OR "work re-integration" OR "job reintegration" OR "job re-integration" OR "work resumption" |
| S5 | (MH "Job Re-Entry") |
| S4 | AB "ankle injur*" OR "arm injur*" OR back OR "carpal tunnel syndrome" OR "cumulative trauma disorder*" OR "foot injur*" OR "hand injur*" OR "hip injur*" OR "knee injur*" OR "leg injur*" OR "lower extremit*" OR msk OR muscle OR musculoskeletal OR myalgia OR neck OR "shoulder pain" OR “shoulder injur*” OR sprain* OR strain* OR tend* OR "tension neck" OR "upper extremit*" OR "whiplash injur*" OR "work msk" OR "wrist injur*" OR "acute stress disorder*" OR "adjustment disorder*" OR agoraphobia OR anxiety OR "anxiety disorders" OR burnout OR "common mental disorder*" OR depression OR depressive OR dysthymia OR "emotional distress" OR "mental disorder*" OR "mental health" OR "mental illness" OR "mood disorder*" OR obsessive-compulsive OR panic OR phobia OR phobic OR "phobic disorders" OR "posttraumatic stress disorder" OR "premenstrual dysphoric disorder" OR ptsd OR "psychological distress" OR "psychological disturbance" OR "psychological health" OR "seasonal affective disorder" OR "seasonal depression" OR "selective mutism" OR "trauma-and stressor-related disorders" OR "winter blues" |
| S3 | TI "ankle injur*" OR "arm injur*" OR back OR "carpal tunnel syndrome" OR "cumulative trauma disorder*" OR "foot injur*" OR "hand injur*" OR "hip injur*" OR "knee injur*" OR "leg injur*" OR "lower extremit*" OR msk OR muscle OR musculoskeletal OR myalgia OR neck OR "shoulder pain" OR “shoulder injur*” OR sprain* OR strain* OR tend* OR "tension neck" OR "upper extremit*" OR "whiplash injur*" OR "work msk" OR "wrist injur*" OR "acute stress disorder*" OR "adjustment disorder*" OR agoraphobia OR anxiety OR "anxiety disorders" OR burnout OR "common mental disorder*" OR depression OR depressive OR dysthymia OR "emotional distress" OR "mental disorder*" OR "mental health" OR "mental illness" OR "mood disorder*" OR obsessive-compulsive OR panic OR phobia OR phobic OR "phobic disorders" OR "posttraumatic stress disorder" OR "premenstrual dysphoric disorder" OR ptsd OR "psychological distress" OR "psychological disturbance" OR "psychological health" OR "seasonal affective disorder" OR "seasonal depression" OR "selective mutism" OR "trauma-and stressor-related disorders" OR "winter blues" |
| S2 | (MH "Stress Disorders, Post-Traumatic") OR (MH "Anxiety Disorders+") OR (MH "Obsessive-Compulsive Disorder") OR (MH "Panic Disorder") OR (MH "Agoraphobia") OR (MH "Phobic Disorders+") OR (MH "Adjustment Disorders+") OR (MH "Anxiety") OR (MH "Depression") OR (MH "Burnout, Professional") OR (MH "Stress, Occupational") OR (MH "Mental Health") OR (MH "Affective Disorders+") OR (MH "Premenstrual Dysphoric Disorder") OR (MH "Seasonal Affective Disorder") OR (MH "Mutism") |
| S1 | (MH "Ankle Injuries+") OR (MH "Arm Injuries+") OR (MH "Back") OR (MH "Back Injuries") OR (MH "Low Back Pain") OR (MH "Carpal Tunnel Syndrome") OR (MH "Cumulative Trauma Disorders+") OR (MH "Foot Injuries+") OR (MH "Hand Injuries+") OR (MH "Hip Injuries+") OR (MH "Knee Injuries+") OR (MH "Leg Injuries+") OR (MH "Lower Extremity+") OR (MH "Muscle, Skeletal+") OR (MH "Tendons+") OR (MH "Musculoskeletal System+") OR (MH "Muscle Pain") OR (MH "Neck") OR (MH "Neck Pain") OR (MH "Neck Injuries+") OR (MH "Whiplash Injuries") OR (MH "Shoulder Pain") OR (MH "Shoulder Injuries+") OR (MH "Sprains and Strains+") OR (MH "Tendon Injuries+") OR (MH "Upper Extremity+") OR (MH "Wrist Injuries+") |

**PsycINFO database**

| 1 | exp ankle/ |
| --- | --- |
| 2 | "arm (anatomy)"/ |
| 3 | "back (anatomy)"/ or back pain/ |
| 4 | exp "feet (anatomy)"/ |
| 5 | exp "hand (anatomy)"/ |
| 6 | hips/ |
| 7 | knee/ |
| 8 | exp "leg (anatomy)"/ |
| 9 | muscles/ |
| 10 | Musculoskeletal System/ |
| 11 | "neck (anatomy)"/ |
| 12 | "shoulder (anatomy)"/ |
| 13 | exp tendons/ |
| 14 | whiplash/ |
| 15 | wrist/ |
| 16 | acute stress disorder/ |
| 17 | Adjustment Disorders/ |
| 18 | agoraphobia/ |
| 19 | Anxiety Disorders/ or Anxiety/ |
| 20 | occupational stress/ |
| 21 | affective disorders/ or seasonal affective disorder/ or premenstrual dysphoric disorder/ |
| 22 | Dysthymic Disorder/ |
| 23 | Mental Health/ |
| 24 | Obsessive Compulsive Disorder/ |
| 25 | panic disorder/ or panic/ |
| 26 | exp phobias/ |
| 27 | Posttraumatic Stress Disorder/ |
| 28 | elective mutism/ |
| 29 | emotional trauma/ |
| 30 | 1 or 2 or 3 or 4 or 5 or 6 or 7 or 8 or 9 or 10 or 11 or 12 or 13 or 14 or 15 or 16 or 17 or 18 or 19 or 20 or 21 or 22 or 23 or 24 or 25 or 26 or 27 or 28 or 29 |
| 31 | ("ankle injur*" or "arm injur*" or back or "carpal tunnel syndrome" or "cumulative trauma disorder*" or "foot injur*" or "hand injur*" or "hip injur*" or "knee injur*" or "leg injur*" or "lower extremit*" or msk or muscle or musculoskeletal or myalgia or neck or "shoulder pain" or "shoulder injur*" or sprain* or strain* or tend* or "tension neck" or "upper extremit*" or "whiplash injur*" or "work msk" or "wrist injur*" or "acute stress disorder*" or "adjustment disorder*" or agoraphobia or anxiety or "anxiety disorders" or burnout or "common mental disorder*" or depression or depressive or dysthymia or "emotional distress" or "mental disorder*" or "mental health" or "mental illness" or "mood disorder*" or obsessive-compulsive or panic or phobia or phobic or "phobic disorders" or "posttraumatic stress disorder" or "premenstrual dysphoric disorder" or ptsd or "psychological distress" or "psychological disturbance" or "psychological health" or "seasonal affective disorder" or "seasonal depression" or "selective mutism" or "trauma-and stressor-related disorders" or "winter blues").ab. or ("ankle injur*" or "arm injur*" or back or "carpal tunnel syndrome" or "cumulative trauma disorder*" or "foot injur*" or "hand injur*" or "hip injur*" or "knee injur*" or "leg injur*" or "lower extremit*" or msk or muscle or musculoskeletal or myalgia or neck or "shoulder pain" or "shoulder injur*" or sprain* or strain* or tend* or "tension neck" or "upper extremit*" or "whiplash injur*" or "work msk" or "wrist injur*" or "acute stress disorder*" or "adjustment disorder*" or agoraphobia or anxiety or "anxiety disorders" or burnout or "common mental disorder*" or depression or depressive or dysthymia or "emotional distress" or "mental disorder*" or "mental health" or "mental illness" or "mood disorder*" or obsessive-compulsive or panic or phobia or phobic or "phobic disorders" or "posttraumatic stress disorder" or "premenstrual dysphoric disorder" or ptsd or "psychological distress" or "psychological disturbance" or "psychological health" or "seasonal affective disorder" or "seasonal depression" or "selective mutism" or "trauma-and stressor-related disorders" or "winter blues").ti. |
| 32 | 30 or 31 |
| 33 | work related illnesses/ |
| 34 | Employee Absenteeism/ |
| 35 | Employee Leave Benefits/ |
| 36 | (absence or absenteeism or disability or "off work" or off-work or sick or "sick leave" or sick-listed or sickness or "sickness absence" or "work interruption" or "work-related injur*" or "work injur*" or "occupational diseas*" or "occupational injur*" or "occupational health").ab. or (absence or absenteeism or disability or "off work" or off-work or sick or "sick leave" or sick-listed or sickness or "sickness absence" or "work interruption" or "work-related injur*" or "work injur*" or "occupational diseas*" or "occupational injur*" or "occupational health").ti. |
| 37 | 33 or 34 or 35 or 36 |
| 38 | reemployment/ |
| 39 | ("back to work" or "job maintenance" or "job reentry" or "job re-entry" or "job retention" or "labor force participation" or presenteeism or reemployment or "resuming work" or "return to work" or return-to-work or rtw or "time at work" or "vocational rehabilitation" or "work re-entry" or "work re-entry" or "work reintegration" or "work re-integration" or "job reintegration" or "job re-integration" or "work resumption").ab. or ("back to work" or "job maintenance" or "job reentry" or "job re-entry" or "job retention" or "labor force participation" or presenteeism or reemployment or "resuming work" or "return to work" or return-to-work or rtw or "time at work" or "vocational rehabilitation" or "work re-entry" or "work re-entry" or "work reintegration" or "work re-integration" or "job reintegration" or "job re-integration" or "work resumption").ti. |
| 40 | 38 or 39 |
| 41 | 32 and 37 and 40 |

**PubMed database**

((("back to work"[Title/Abstract] OR "job maintenance"[Title/Abstract] OR "job reentry"[Title/Abstract] OR "job re-entry"[Title/Abstract] OR "job retention"[Title/Abstract] OR "labor force participation"[Title/Abstract] OR presenteeism[Title/Abstract] OR reemployment[Title/Abstract] OR "resuming work"[Title/Abstract] OR "return to work"[Title/Abstract] OR return-to-work[Title/Abstract] OR rtw[Title/Abstract] OR "time at work"[Title/Abstract] OR "vocational rehabilitation"[Title/Abstract] OR "work re-entry"[Title/Abstract] OR "work re-entry" [Title/Abstract] OR "work reintegration"[Title/Abstract] OR "work re-integration"[Title/Abstract] OR "job reintegration"[Title/Abstract] OR "job re-integration"[Title/Abstract] OR "work resumption"[Title/Abstract]) OR ("Return to Work"[Mesh])) AND ((absence[Title/Abstract] OR absenteeism[Title/Abstract] OR disability[Title/Abstract] OR "off work"[Title/Abstract] OR off-work[Title/Abstract] OR sick[Title/Abstract] OR "sick leave"[Title/Abstract] OR sick-listed[Title/Abstract] OR sickness[Title/Abstract] OR "sickness absence"[Title/Abstract] OR "work interruption"[Title/Abstract] OR "work-related injur*"[Title/Abstract] OR "work injur*"[Title/Abstract] OR "occupational diseas*"[Title/Abstract] OR "occupational injur*"[Title/Abstract] OR "occupational health"[Title/Abstract]) OR ((((("Sick Leave"[Mesh]) OR "Absenteeism"[Mesh]) OR "Occupational Injuries"[Mesh]) OR "Accidents, Occupational"[Mesh]) OR "Occupational Diseases"[Mesh]))) AND ((("ankle injur*"[Title/Abstract] OR "arm injur*"[Title/Abstract] OR back[Title/Abstract] OR "carpal tunnel syndrome"[Title/Abstract] OR "cumulative trauma disorder*"[Title/Abstract] OR "foot injur*"[Title/Abstract] OR "hand injur*"[Title/Abstract] OR "hip injur*"[Title/Abstract] OR "knee injur*"[Title/Abstract] OR "leg injur*"[Title/Abstract] OR "lower extremit*"[Title/Abstract] OR msk[Title/Abstract] OR muscle[Title/Abstract] OR musculoskeletal[Title/Abstract] OR myalgia[Title/Abstract] OR neck[Title/Abstract] OR "shoulder pain"[Title/Abstract] OR “shoulder injur*”[Title/Abstract] OR sprain*[Title/Abstract] OR strain*[Title/Abstract] OR tend*[Title/Abstract] OR "tension neck"[Title/Abstract] OR "upper extremit*"[Title/Abstract] OR "whiplash injur*"[Title/Abstract] OR "work msk"[Title/Abstract] OR "wrist injur*"[Title/Abstract] OR "acute stress disorder*"[Title/Abstract] OR "adjustment disorder*"[Title/Abstract] OR agoraphobia[Title/Abstract] OR anxiety[Title/Abstract] OR "anxiety disorders"[Title/Abstract] OR burnout[Title/Abstract] OR "common mental disorder*"[Title/Abstract] OR depression[Title/Abstract] OR depressive[Title/Abstract] OR dysthymia[Title/Abstract] OR "emotional distress"[Title/Abstract] OR "mental disorder*"[Title/Abstract] OR "mental health"[Title/Abstract] OR "mental illness"[Title/Abstract] OR "mood disorder*"[Title/Abstract] OR obsessive-compulsive[Title/Abstract] OR panic[Title/Abstract] OR phobia[Title/Abstract] OR phobic[Title/Abstract] OR "phobic disorders"[Title/Abstract] OR "posttraumatic stress disorder"[Title/Abstract] OR "premenstrual dysphoric disorder"[Title/Abstract] OR ptsd[Title/Abstract] OR "psychological distress"[Title/Abstract] OR "psychological disturbance"[Title/Abstract] OR "psychological health"[Title/Abstract] OR "seasonal affective disorder"[Title/Abstract] OR "seasonal depression"[Title/Abstract] OR "selective mutism"[Title/Abstract] OR "trauma-and stressor-related disorders"[Title/Abstract] OR "winter blues"[Title/Abstract]) OR ((((((((((((((((((((("Stress Disorders, Traumatic, Acute"[Mesh]) OR "Adjustment Disorders"[Mesh]) OR "Agoraphobia"[Mesh]) OR "Anxiety"[Mesh]) OR "Anxiety Disorders"[Mesh]) OR "Burnout, Psychological"[Mesh]) OR "Burnout, Professional"[Mesh]) OR "Depression"[Mesh]) OR "Depressive Disorder"[Mesh]) OR "Dysthymic Disorder"[Mesh]) OR "Psychological Distress"[Mesh]) OR "Mental Health"[Mesh]) OR "Mood Disorders"[Mesh]) OR "Obsessive-Compulsive Disorder"[Mesh]) OR "Panic Disorder"[Mesh]) OR "Phobic Disorders"[Mesh]) OR "Stress Disorders, Post-Traumatic"[Mesh]) OR "Premenstrual Dysphoric Disorder"[Mesh]) OR "Seasonal Affective Disorder"[Mesh]) OR "Mutism"[Mesh]) OR "Trauma and Stressor Related Disorders"[Mesh])) OR (((((((((((((((((((((((("Ankle Injuries"[Mesh]) OR "Arm Injuries"[Mesh]) OR "Low Back Pain"[Mesh]) OR "Carpal Tunnel Syndrome"[Mesh]) OR "Cumulative Trauma Disorders"[Mesh]) OR "Foot Injuries"[Mesh]) OR "Hand Injuries"[Mesh]) OR "Hip Injuries"[Mesh]) OR "Knee Injuries"[Mesh]) OR "Leg Injuries"[Mesh]) OR "Lower Extremity"[Mesh]) OR "Muscle, Skeletal"[Mesh]) OR "Musculoskeletal Pain"[Mesh]) OR "Myalgia"[Mesh]) OR "Neck Pain"[Mesh]) OR "Shoulder Pain"[Mesh]) OR "Shoulder Injuries"[Mesh]) OR "Sprains and Strains"[Mesh]) OR "Tendon Injuries"[Mesh]) OR "Neck Injuries"[Mesh]) OR "Back Injuries"[Mesh]) OR "Upper Extremity"[Mesh]) OR "Whiplash Injuries"[Mesh]) OR "Wrist Injuries"[Mesh]))
