# Supplementary material for: Systematic review and tools appraisal of prognostic factors of return to work in workers on sick leave due to musculoskeletal and common mental disorders
Source: PLoS One. 2024 Jul 17;19(7):e0307284. doi: 10.1371/journal.pone.0307284 (PMC11253986; doi:10.1371/journal.pone.0307284)
Supplement: S2 File — (DOCX) [file pone.0307284.s002.docx]

**Supplementary material 2**

**Table S1**. Key characteristics of included studies

| **Reference** | **Exact RTW Outcome** | **Follow-up time (months)** | **Study design (observational, interventional)** | **Number of Participants (MSD/CMD)** | **Study population (MSD/CMD)** | **Study population/Sector (when available)** |
| --- | --- | --- | --- | --- | --- | --- |
| Abásolo et al., 2008[1] | Duration of temporary work disability (TWD), defined as days of sick leave per episode | 2 years | Interventional | 3,311 | MSD | Patients from health areas 4, 7, and 9 of Madrid, Spain who received a temporary work disability initiation due to MSD  Management/intellectual 7.6%; Services 22.3%; Secretarial 25.7%; Specialized or non-specialized manual work 44.4% |
| Adams et al., 2017[2] | Partial or full RTW after the termination of rehabilitation program | 1 month | Interventional | 80 | CMD | Participants were referred to an occupational rehabilitation service in Ontario, Canada |
| Amick et al., 2017 [3] | Return to work (RTW) status | 6 and 12 months | Observational | 577 | MSD | R-RTW Cohort (prospective study of Ontario workers filing a new Workplace Safety and Insurance Board (WSIB) lost-time claim for a work-related back or upper extremity (UE) MSK disorder) |
| Asher et al., 2017 [4] | Time to RTW (period between operation and date of returning to work) | 3 months | Observational | 4,694 | MSD | Patients undergoing elective spine surgery for degenerative lumbar disease in a prospective multicenter Quality Outcomes Database (QOD) registry (74 participating centers across 26 US states) |
| Baltov et al., 2008 [5] | RTW (dichotomized: RTW or not) | 1,5 months and 3 months | Interventional | 28 | MSD | Patients with chronic whiplash associated disorders (WAD) participating in the Evaluation, Development and Professional Reinte-gration Program of the Jewish Rehabilitation Hospital in Laval  Occupation: Manual 28.6%; Mixed 28.6%; Non-manual 42.8% |
| Bontoux et al., 2004 [6] | RTW (presence at work; duration and nature of the job such as adapted job or changed job) | 12 months | Interventional | 87 | MSD | Patients with chronic low back pain participating in a multidisciplinary disability management program. |
| Bosman et al., 2019 [7] | Registered sick leave data | 180 days | Observational | 103 | MSD | Employees with low back pain (LBP) sick leave after visiting an occupational physician |
| Brouwer et al., 2015 [8] | RTW status groups (RTW, Not-RTW) | 6 months and 12 months | Observational | 446 (6-month interview) and 383 (12-month interview) | MSD | Prospective cohort study of lost-time claimants with work-related low back or upper extremity musculoskeletal pain, who were recruited in cooperation with the Workplace Safety and Insurance Board (WSIB) of Ontario, Canada |
| Brouwer et al., 2010 [9] | Time to RTW | 10 months | Observational | 342–352/238–245 | MSD/CMD | Prospective 1-year cohort of workers on sick leave with different types of symptoms  were recruited from Occupational Health Services (OHSs)  covering three large regions in the Netherlands |
| Carriere et al., 2015 [10] | Return to full time work (yes/no) | 1 year | Observational | 109 | MSD | Patients with work-related musculoskeletal disorders from primary care physiotherapy clinics |
| Cole et al., 2002 [11] | Cumulative number of calendar days during which the worker received 100% benefits within the year after the injury came from the OWCB benefits database | 52 weeks | Observational | 1566 | MSD | Ontario-wide cohort of injured workers with lost-time claims for soft-tissue disorders in conjunction with the Ontario Workers’ Compensation Board (OWCB)  Agriculture, forestry, mining 0.8%; Construction 4.8%; Public administration, defence 8.0%; Transportation, communications 8.9%; Trade, finance, insurance 19.4%; Manufacturing 24.6%; Community, business, service 30.3% |
| Corbière et al., 2017 [12] | RTW | 6 months | Observational | 206/157 | MSD/CMD | Workers on sick leave due to MSDs and CMDs from Quebec, Canada recruited from work rehabilitation clinics  CMD and MSD patients: Public sector 59% and 30%; Private companies MSD patients 70% |
| Cougot et al., 2015 [13] | RTW | 24 months | Interventional | 120 | MSD | Chronic low back pain healthcare workers and other workers after rehabilitation coupled with an occupational intervention |
| Dersh et al., [14] | RTW (any period of work during the post-treatment year) | 1 year | Interventional | 1,200 | MSD | Patients with chronic disabling occupational spinal disorders, who entered treatment in an interdisciplinary functional restoration program at a regional referral centre |
| Du Bois & Donceel, 2008 [15] | Return to the previous occupational level after the first day of sick leave | 3 months | Observational | 186 | MSD | Patients entitled to sickness allowance by the Alliance of Christian Sickness Funds because of low back pain |
| Du Bois et al., 2009 [16] | Time to return to the same or any other job | 6 months | Observational | 346 | MSD | Disabled workers applying for compensation benefit because of low back pain at the Alliance of Christian Sickness Funds |
| Faber et al., 2006 [17] | Time to RTW (period from inclusion in the study to RTW in the original job or another job without reduced time or duties. Not regarded as RTW when shorter than 1 week) | 6 months | Observational | 103 | MSD | Employees on sickness absence due to nonspecific low back pain recruited in the Dutch province Zuid Holland |
| Fishbain et al., 1997[18] | Final employment status | 30 months | Observational | 128 | MSD | Chronic pain patients' (CPPs) after pain facility treatment |
| Franche et al., 2007 [19] | Work absence duration as a continuous variable (total number of full days of work they missed due to their original workplace injury) | 6 months | Observational | 431 | MSD | Lost-time claimants with work-related back or upper extremity musculoskeletal disorders were recruited in cooperation with the Workplace Safety and Insurance Board (WSIB) of Ontario, Canada  Automotive, manufacturing, steel 19.0%; Service 22.3%; Education, municipal, schedule 2 23.9%; Healthcare 14.6%; Transpiration 9.3%; Chemical/processing, electrical, food 5.5%; Construction 2.5%; Agriculture, forest, pulp and paper, mining 2.7%; Unknown 0.2% |
| Gaines et al., 1999 [20] | Time for return to regular work without restrictions | every 7-10 calendar days by the occupational medicine physician | Observational | 55 | MDS | Patients with work-related, acute low back pain within a physician multispecialty clinic |
| Gauthier et al., 2006 [21] | Return-to-work information and claim status obtained from WCB files | 4 weeks | Interventional | 255 | MSD | Claimants of the Workers Compensation Board (WCB) of Nova Scotia who were referred to a community-based secondary prevention program  Labourer 32.5%; Nursing 18.0%; Fishing 10.2%; Driving 4.7%; Retail 8.2%; Trade 20%; Clerical 2.0%; Restaurant 4.3% |
| Gross & Battie, 2010 [22] | Days until suspension of time-loss benefits | 1 year | Observational | 164 | MSD | Workers' compensation claimants receiving time-loss benefits undergoing return-to-work assessment at the Alberta Workers' Compensation Board (WCB) rehabilitation facility in Canada |
| Gross & Battie, 2005 [23] | Time to Total Temporary Disability Benefit Suspension | 1 year | Observational | 45 | MSD | Injured workers receiving extended time-loss benefits for low back injuries from the main Alberta Workers’ Compensation Board (WCB) administrative database |
| Grøvle et al., 2013 [24] | Return to full-time work | 2 years | Observational | 246 | MSD | Patients with sciatica and disc herniation referred to back clinics at four hospitals in South eastern Norway |
| Gustafsson et al., 2013 [25] | Full RTW (no sickness absence or disability pension after multidisciplinary medical assessment); Partial RTW (part-time sickness absence or disability pension after multidisciplinary medical assessment) | 3 years | Observational | 905 | CMD | Long-term sickness absentees who had undergone a multidisciplinary medical assessment |
| Hagen et al., 2005 [26] | Sick leave (total length of leave) | 3 and 12 months | Interventional | 457 | MSD | Patients sick-listed for lower back pain with or without radiating pain referred to a spine clinic group or a primary healthcare group |
| Haldorsen et al., 1998 [27] | Return to work status | 12 months | Interventional | 260 | MSD | Patients with back pain (with or without radiation to the legs) on sick leave under evaluation for continued leave (Norwegian Sickness Certificate II) |
| Hara et al., 2018 [28] | (Re)entry to the ordinary work force (dichotomous as participation in competitive work ≥ 1 day (7.5 h) per week on average over 8 weeks) | RTW 1 day per week for 8 weeks | Interventional | 212 | MSD/CMD | Sick listed individuals on temporary work-disability benefits participated in an occupational rehabilitation program |
| Haveraaen et al., 2017 [29] | Days until first RTW, from the start of the treatment until the first day the employee re-entered employment (partially or fully) | 18 months | Interventional | 543 | MSD/CMD | Sick listed employees participating in different Rapid-RTW programmes in Norway  Private sector 36.2%; Public-Municipal level 24.2%; Public-Regional and governmental level 9.3%; Private-Publicly financed sector 3.4%; Self-employed 1.2% |
| Haveraaen et al., 2015 [30] | Sickness absence retrieved from the Norwegian Social Insurance Register (FD-Trygd) | 3 months | Interventional | 251 | MSD/CMD | Sick listed employees participating in different Rapid-RTW programmes in Norway  Private sector 45.4%; Public-Municipal level 33.9%; Public-Region and governmental level 13.1%; Private-public financed sector; 4.4%; Self-employment 1.2% |
| Hedlund et al., 2022 [31] | Return to work was measured using the question ‘Enter your per-centage of work’ and was defined in two different ways, depending on whether a woman was on full- or part-time sick leave at baseline. If she was on full-time sick leave at baseline, RTW was defined as having begun working. If she worked part-time at baseline, RTW was defined as having increased the percentage of work. | RTW 1 year | Observational | 45 | CMD | Women from two regions in central Sweden having either a physician's diagnosis within mood disorders) and/or neurotic, stress-related and somatoform syndromes, on sick leave for more than 2 months but less than 2 years  Health care, schools and social service, 48%; Administrative work, 20%; Sales and service, 8%; Industry workers and engineers, 12% |
| Hogg-Johnson & Cole, 2003 [32] | Duration on total temporary wage replacement benefits | 4 weeks, 10 weeks, 16 weeks, and 52 weeks | Observational | 907 | MSD | Injured workers with lost-time claims for soft tissue disorders as part of a larger programme evaluation in conjunction with the Ontario Workers’ Compensation Board (now Ontario Workplace Safety & Insurance Board or OWSIB) |
| Huijs et al., 2012 [33] | Length of time in calendar days from the start of sickness absence until full RTW (working at least the number of hours specified in their employment contract) | 2 years | Observational | 359/159 | MSD/CMD | Dutch employees on long-term sick leave recruited from the register of the Dutch Social Security Agency |
| Hunter et al., 1998 [34] | Employment status and long-term impact of treatment on patients obtained from employment records | 3.9 to 6.8 years after completion of rehabilitation | Interventional | 178 | MSD | Consecutive railroad employees who were treated at a free-standing orthopaedic rehabilitation centre |
| Iles et al., 2020 [35] | Number of days a worker received payments | 1 and 3 months | Observational | 524 | MSD | Workers’ compensation claimants in the state of New South Wales, Australia |
| Jacobsen et al., 2020 [36] | (Re)entry to the ordinary work force (dichotomous as participation in competitive work ≥2.5 day (18.75 h) per week, using four different time periods with 14 weeks between each time point) | Up to 1 year | Interventional | 137 | MSD/CMD | Participants with temporary medical benefits due to work incapacity, persistent pain, fatigue, depression, or anxiety referred to inpatient rehabilitation wanting to RTW |
| Katz et al., 2005 [37] | Work status, (dichotomous indicator, whether the patient was working (full and part time) or not) | 6 and 12 months | Observational | 181 | MSD | Workers scheduled for carpal tunnel release in a community-based cohort  Managerial, professional, technician 26%; Sales, service, administrative 20%; Manual labor 54% |
| Koopman et al., 2004 [38] | Number of working hours per week compared with the number of hours worked per week at appointment (a categorical variable was used divided into “resumers”, “partly resumers”, and “non-resumers”) | 1 year | Interventional | 42 | MSD | Participants with chronic low back pain for more than six months admitted to the training program by occupational physicians or medical advisors of an insurance company |
| Kvam et al., 2015 [39] | Full-time work, part-time work, full work assessment allowance (WAA) and full disability pension were computed into 1 for full-time work, 2 for part-time work and 3 for full WAA and full disability pension | 6–12 months after the rehabilitation program | Interventional | 187 | MSD | People with complex health conditions such as musculoskeletal pain sometimes in combination with mild psychological problems enrolled in one of two vocational rehabilitation institutions with a combination of rural and small-town characteristics in Mid-Norway |
| Lagerveld et al., 2017 [40] | Duration until full RTW in calendar days from the baseline measurement until full RTW (working the number of hours specified in the labour contract, except in the case of adjusted tasks and/or reduced responsibilities) | 9 months | Observational | 168 | CMD | Employees on sick leave due to CMD recruited via a mental health centre where they would receive cognitive–behavioural therapy (CBT) |
| Lagerveld et al., 2010 [41] | RTW as the current work status compared to regular working hours defined by contract: no RTW, partial RTW (temporarily working fewer than full contract hours) or full RTW | 3 months | Observational | 245 | MSD/CMD | Sick-listed Dutch employees with health problems, either mental or physical |
| Laukkala, et al., 2018 [42] | Return to work | 1 year | Observational | 115-116 | CMD | Psychiatric patients who were referred for a thorough psychiatric examination and assessment of function and work ability  Manual workers 9%; Lower-level non-manual workers 24%; Upper-level non-manual workers 18%, Managers 1%; Entrepreneurs 8% |
| Lepièce et al., 2017 [43] | Effective return to work (RTW) | 6 months | Observational | 134 | CMD | Patients hospitalized for a period of 10 days in the Psychosomatic Rehabilitation Day Centre of CHU UCL Godinne, Belgium  Public administration 21.5%; Health 16.2%; Education 15.4%; Other 46.9% |
| Lötters & Burdorf, 2006 [44] | Duration of sickness absence | 12 months | Observational | 253 | MSD | Patients on sick leave due to non-specific musculoskeletal disorders recruited by occupational health physicians during their consults or selected from the absenteeism register of a large Dutch occupational health service |
| Løvvik et al., 2014 [45] | Benefit recipiency (dichotomised in any health-related benefits (disability pension, work assessment allowance, unemployment benefit or sickness benefits) from the national welfare service and those who did not receive any such benefits) | 6 months | Interventional | 1193 | CMD | Persons at risk of sick leave, currently on sick leave or on long-term benefits due to CMD  Blue-collar workers 33.9% |
| Lydell et al., 2011 [46] | Certified sick leave | 10 years | Interventional | 186 | MSD | Working-aged sick-listed people due to MSD referred from  either a physician or the Regional Social Insurance  Office to participate in the rehabilitation program |
| Lydell et al. 2009 [47] | Certified sick leave | 5 and 10 years | Interventional | 126-131 | MSD | Working-age people sick-listed due to MSD after a referral from either a physician or the Regional Social Insurance Office  Blue-collar worker 50.3%; White-collar worker 31.1%;  Farmer 8.7%; Company owner 1.6% |
| Momsen et al., 2014 [48] | Return to work defined as receiving no social transfer payment (except unemploy-  ment benefits) during a specific week | 1 and 2 years | Interventional | 282 | MSD | Sick-listed employees with nonspecific LBP referred from general practices to the Spine Center, Regional Hospital Silkeborg, Denmark |
| Netterstrøm et al., 2015 [49] | No economic compensation of any type at the time of census | 1 year | Interventional | 223 | CMD | People on sick leave due to work-related CMDs  Intervention and Exclusion group: Blue collar workers 34.3% and 30.8% |
| Nicholas et al., 2019 [50] | Lost time from work (days to return to pre-injury duties) | 12 months | Interventional | 163 | MSD | Health care workers (nurses, security staff, orderlies, technicians, managers, administrative staff, and paramedics) from participating hospitals who reported soft tissue injuries accepted (by the insurer) as work-related, made a claim for workers compensation insurance coverage, and had their injury confirmed by their Nominated Treating Doctor (NTD) |
| Nielsen et al., 2012 [51] | Time to RTW  defined as no longer receiving sickness absence or unemployment benefits | 1 year | Observational | 205 | CMD | Sick-listed employees due to CMD, such as, stress, depression,  burnout, and anxiety  Governmental 18.5%; Private 48.8%; Municipal 32.7% |
| Nielsen et al., 2011 [52] | Time to RTW defined as when their weekly entry in National Register for Social Transfer Payments (DREAM) became a blank | 1 year | Observational | 644 | CMD | Sick-listed participants from the Job Centre Copenhagen, a sub-unit of the municipality with mental health problems  Management 1.4%; Administration 20.1%; Trade 8.2%; Service 12.1%; Manual workers 9.6% Health care 11.7%; Social work 20.1%; Education 4.7% |
| Nieuwenhuijsen et al., 2006 [53] | Duration of sickness absence measured by calculating the time to full return to work during the follow-up period, regardless of  the timing of any partial return to work (working the same number  of hours as prior to the sickness absence episode, for at  least 1 week) | 1 year | Observational | 168 | CMD | Teachers on sick leave with CMD |
| Nieuwenhuijsen et al., 2005 [54] | Time to full return to work during the follow-up period (same number of hours as before the sickness absence) | 1 year | Observational | 174 | CMD | Sick-listed employees due to CMD  Woking in education sector 57% |
| Nieuwenhuijsen et al., 2004 [55] | Time to full return to work as the number of days between the first day of sickness absence and the first day of full return (same number of hours as prior to the sickness absence) | 1 year | Observational | 83 | CMD | Sick-listed employees with mental health problems  Education 63%; Public administration 11%; Real estate, renting and business activities 10%; Transport, storage and communication 8%; Health and social work 5%; Manufacturing 2%; Wholesale and retail 1% |
| Norder et al., 2017 [56] | Sickness absence  from the day of reporting sick to the day of full return to work (i.e. working the same number of hours per week as before CMD sickness absence) | 1 year | Observational | 211 | CMD | Employees working in companies with a sickness absence insurance and who reported sick with CMDs  Administrator 20 %; Manager 10 %; Healthcare professional 10 %; Consultant 10 %; Project leader and supervisor 8 %; or teacher 7 % |
| Nordin et al., 2002 [57] | Return to work | 6 months | Observational | 1288 | MSD | Workers of a privately held utility company (approximately 14,000 workers) and a public transportation authority (approximately 48,000 employees) experiencing work-limiting episodes of low back pain |
| Okurowski et al., 2003 [58] | Work status at determined by searching the computerized claims file for an indemnity payment transaction | 6 months | Observational | 986 | MSD | Cases who were out of work at 3 months postinjury as the result of uncomplicated occupational low back pain |
| Opsahl et al., 2016 [59] | Return to work | 1 year | Interventional | 569 | MSD | Employees with sick leave due to lower back pain from the Cognitive interventions and nutritional supplements trial (CINS Trial) |
| Oyeflaten et al., 2008 [60] | RTW defined as return to work-related activity (return to ordinary work, return with adjusted work tasks, new work tasks/same employer, new employer and “work related re-employment” (paid by the public health insurance or labour-agency)) | 3 months and 1 year | Interventional | 95-113 | MSD | Long-term sick-listed individuals admitted to the rehabilitation centre based on referrals from their general practitioners (GP), National Health Insurance offices or labour marked agencies  Blue-collar workers 18%; White-collar workers 22%; School or kindergarten 16%; Healthcare sector 26%; Service field 16% |
| Post et al., 2006 [61] | Full RTW was defined as working according to the number of hours of the initial work contract | 10 months | Observational | 335/239 | MSD/CMD | Employees on sick leave recruited from local offices of a nationwide occupational health service (OHS) in three large regions in the Netherlands |
| Rashid et al., 2021 [62] | Two questions: “Are you working right now?” and “To what extent are you working?”. If participants worked > 50% of their extent of employment at baseline, they were categorized as RTW; otherwise, they were categorized as not RTW (NRTW). | RTW 1 year | Observational | 141 | MSD | Women selected by the Swedish Social Insurance Agency based on their medical certificate, on ≥ 50% sick leave from her usual employment and on sick leave for ≥ 1 month due to long-term neck/shoulder and/or back pain that had lasted for ≥ 3 months.  White-collar, 37%; Blue-collar 63% |
| Reme et al., 2009 [63] | Sick leave based on register data from local insurance offices and self-report (Non RTW included sick leave as well as rehabilitation and disability benefit) | 3 months and 1 year | Interventional | 176 | MSD | Sick-listed low back pain patients invited by the Norwegian Labour and Welfare Administration (NAV) in 5 neighbour communities of a spine clinic |
| Sampere et al., 2012 [64] | Time to return to work calculated from the difference in days between the date of the end and the start of sick leave episode, plus one (end of the sick leave due to recovery or improvement and if they were still employed by the same company) | 24 months | Observational | 314/119 | MSD/CMD | Workers from companies in the Spanish provinces of Barcelona and Madrid with current non-work-related sick leave episode |
| Schultz et al., 2005 [65] | RTW status | 3 months | Observational | 100 | MSD | Participants off work due to subacute low back injury with a low back injury claim with the Workers’ Compensation Board of British Columbia (WCB) and the reception of WCB benefits |
| Schultz et al., 2002 [66] | RTW status | 3 months | Observational | 253 | MSD | Compensated low back–injured workers with a low back injury claim with the Workers’ Compensation Board of British Columbia (WCB-BC), being on active wage loss and receiving WCB benefits |
| Schultz et al., 2004 [67] | Return-to-work status | 3 months | Observational | 253 | MSD | Workers' Compensation Board of British Columbia (WCB-BC) claimants with non-specific low back injuries |
| Selander et al., 2007 [68] | Successful rehabilitation: a lower degree of sickness allowance, or none compared to the start of rehabilitation, unsuccessful rehabilitation: sickness allowance to the same extent or more compared to the start of rehabilitation | 6 months | Interventional | 347 | MSD | Clients on long-term sick leave due to back pain problems participating in a rehabilitation programme in Sweden |
| Skarpaas et al., 2019 [69] | Time to first RTW and first full RTW (days from when the employee started treatment until the first day back at work, either partial or full job size (first RTW), and until the employee for the first time returned to work in the same job size they had before (first RTW or full RTW)) | 360 days | Interventional | 185/45 | MSD/CMD | Employees on full-time sick leave, from 43 different rapid-RTW programmes in Norway  Public 48%; Private 52% |
| Soucy et al., 2006 [70] | Work status (back to work or not) | 6 months | Observational | 258 | MSD | Workers with subacute low back pain who were on sick leave and receiving compensation from the CSST (Quebec Workers’ Compensation Board) |
| Steenstra et al., 2016 [71] | Time on benefits during a first claim for back pain as the length in calendar days of the first continuous episode of any wage replacement | 2 years | Observational | 113 | MSD | Workers who had a lost-time claim (LTC) for an uncomplicated back injury (strain or sprain) approved by the Workplace Safety and Insurance Board (WSIB) of Ontario  Non-manual 8.8%; Mixed manual 38.9% and Manual 50.4%; Missing 1.8% |
| Steenstra et al., 2005 [72] | Duration of work absenteeism due to non-specific low back pain in calendar days from the first day of sick leave to full RTW for at least 1 day | 26 weeks | Observational | 515 | MSD | Workers calling in sick from work due to low back pain at an occupational health service  IT department 2.8%; Maintenance 5.7%; Laboratory 10.9%; Management 16.3; Medical 5.5%; Paramedical 10.9%; Technical 4.7%; Nursing 34.6%; Other 8.6% |
| Storheim et al., 2005 [73] | Number of days on sick-leave and whether patients returned to full-time work | 1 year | Interventional | 93 | MSD | Patients sick-listed for non-specific sub-acute low back pain  Patients returning to work vs patients not returning to work: Office working/sedentary 42.5% and 20.0%; Light manual handling 28.8% and 50.0%; Heavy manual handling 28.8% and 30.0% |
| Turner et al., 2006 [74] | Replacement compensation for temporary total disability and number of days of wage replacement receipt | 6 months | Observational | 1068 | MSD | Workers who submitted Workers’ Compensation claims for work-related back pain and received at least 1 day of temporary total disability wage replacement |
| van Duijn et al., 2005 [75] | Time until return-to-work on full duty in the regular job | 12 months | Observational | 164 | MSD | Employees on sick leave due to MSD enrolled by occupational health physicians during their consultations or selected from the absenteeism register of a large Dutch occupational health service |
| Victor et al., 2018 [76] | Full or partial RTW clustered together with those still working fully or partly and defined as successful RTW, those working less were clustered together with those still not working as failed RTW | 6 months | Observational | 164 | CMD | Patients treated at an RTW outpatient clinic |
| Wåhlin et al., 2012 [77] | RTW together with an indication of possible part-time RTW | 3 months | Interventional | 432 | MSD/CMD | Sick-listed subjects with musculoskeletal or mental disorders who sought primary healthcare or occupational health service |
| Westman et al., 2008 [78] | Sick leave | 3 years | Interventional | 94 | MSD | Patients with non-acute pain problems in Primary Health Care settings in the county of Västmanland, Sweden |

# **References**

1. Abásolo L, Carmona L, Lajas C, Candelas G, Blanco M, Loza E, et al. Prognostic factors in short-term disability due to musculoskeletal disorders. Arthritis Rheum. 2008;59(4):489-96. Epub 2008/04/03. doi: 10.1002/art.23537. PubMed PMID: 18383421.

2. Adams H, Thibault P, Ellis T, Moore E, Sullivan M. The Relation Between Catastrophizing and Occupational Disability in Individuals with Major Depression: Concurrent and Prospective Associations. J Occup Rehabil. 2017;27(3):405-12. Epub 2016/10/23. doi: 10.1007/s10926-016-9669-7. PubMed PMID: 27770242.

3. Amick BC, 3rd, Lee H, Hogg-Johnson S, Katz JN, Brouwer S, Franche RL, et al. How Do Organizational Policies and Practices Affect Return to Work and Work Role Functioning Following a Musculoskeletal Injury? J Occup Rehabil. 2017;27(3):393-404. Epub 2016/09/23. doi: 10.1007/s10926-016-9668-8. PubMed PMID: 27654622.

4. Asher AL, Devin CJ, Archer KR, Chotai S, Parker SL, Bydon M, et al. An analysis from the Quality Outcomes Database, Part 2. Predictive model for return to work after elective surgery for lumbar degenerative disease. J Neurosurg Spine. 2017;27(4):370-81. Epub 2017/05/13. doi: 10.3171/2016.8.Spine16527. PubMed PMID: 28498069.

5. Baltov P, Côte J, Truchon M, Feldman DE. Psychosocial and socio-demographic factors associated with outcomes for patients undergoing rehabilitation for chronic whiplash associated disorders: a pilot study. Disabil Rehabil. 2008;30(25):1947-55. Epub 2008/07/09. doi: 10.1080/09638280701791245. PubMed PMID: 18608396.

6. Bontoux L, Roquelaure Y, Billabert C, Dubus V, Sancho PO, Colin D, et al. Étude du devenir à un an de lombalgiques chroniques inclus dans un programme associant reconditionnement à l'effort et action ergonomique. Recherche de facteurs prédictifs de retour et de maintien au travail. Annales de Réadaptation et de Médecine Physique. 2004;47(8):563-72. doi: <https://doi.org/10.1016/j.annrmp.2004.03.006>.

7. Bosman LC, Twisk JWR, Geraedts AS, Heymans MW. Development of Prediction Model for the Prognosis of Sick Leave Due to Low Back Pain. J Occup Environ Med. 2019;61(12):1065-71. Epub 2019/10/28. doi: 10.1097/jom.0000000000001749. PubMed PMID: 31651601.

8. Brouwer S, Amick BC, 3rd, Lee H, Franche RL, Hogg-Johnson S. The Predictive Validity of the Return-to-Work Self-Efficacy Scale for Return-to-Work Outcomes in Claimants with Musculoskeletal Disorders. J Occup Rehabil. 2015;25(4):725-32. doi: 10.1007/s10926-015-9580-7. PubMed PMID: 25990375; PubMed Central PMCID: PMCPMC4636988.

9. Brouwer S, Reneman MF, Bültmann U, van der Klink JJ, Groothoff JW. A prospective study of return to work across health conditions: perceived work attitude, self-efficacy and perceived social support. J Occup Rehabil. 2010;20(1):104-12. Epub 2009/11/07. doi: 10.1007/s10926-009-9214-z. PubMed PMID: 19894106; PubMed Central PMCID: PMC2832875.

10. Carriere JS, Thibault P, Sullivan MJ. The Mediating Role of Recovery Expectancies on the Relation Between Depression and Return-to-Work. J Occup Rehabil. 2015;25(2):348-56. doi: 10.1007/s10926-014-9543-4. PubMed PMID: 25252609.

11. Cole DC, Mondloch MV, Hogg-Johnson S. Listening to injured workers: how recovery expectations predict outcomes--a prospective study. CMAJ. 2002;166(6):749-54. Epub 2002/04/12. PubMed PMID: 11944761; PubMed Central PMCID: PMC99453.

12. Corbiere M, Negrini A, Durand MJ, St-Arnaud L, Briand C, Fassier JB, et al. Development of the Return-to-Work Obstacles and Self-Efficacy Scale (ROSES) and Validation with Workers Suffering from a Common Mental Disorder or Musculoskeletal Disorder. J Occup Rehabil. 2017;27(3):329-41. doi: 10.1007/s10926-016-9661-2. PubMed PMID: 27562583.

13. Cougot B, Petit A, Paget C, Roedlich C, Fleury-Bahi G, Fouquet M, et al. Chronic low back pain among French healthcare workers and prognostic factors of return to work (RTW): a non-randomized controlled trial. J Occup Med Toxicol. 2015;10:40. doi: 10.1186/s12995-015-0082-5. PubMed PMID: 26516339; PubMed Central PMCID: PMCPMC4625968.

14. Dersh J, Mayer TG, Gatchel RJ, Polatin PB, Theodore BR, Mayer EA. Prescription opioid dependence is associated with poorer outcomes in disabling spinal disorders. Spine (Phila Pa 1976). 2008;33(20):2219-27. Epub 2008/08/30. doi: 10.1097/BRS.0b013e31818096d1. PubMed PMID: 18725868.

15. Du Bois M, Donceel P. A screening questionnaire to predict no return to work within 3 months for low back pain claimants. Eur Spine J. 2008;17(3):380-5. Epub 2008/01/04. doi: 10.1007/s00586-007-0567-8. PubMed PMID: 18172698; PubMed Central PMCID: PMC2270393.

16. Du Bois M, Szpalski M, Donceel P. Patients at risk for long-term sick leave because of low back pain. Spine J. 2009;9(5):350-9. doi: 10.1016/j.spinee.2008.07.003. PubMed PMID: 18790677.

17. Faber E, Burdorf A, Bierma-Zeinstra SM, Miedema HS, Koes BW. Determinants for improvement in different back pain measures and their influence on the duration of sickness absence. Spine (Phila Pa 1976). 2006;31(13):1477-83. Epub 2006/06/03. doi: 10.1097/01.brs.0000219873.84232.26. PubMed PMID: 16741458.

18. Fishbain DA, Cutler RB, Rosomoff HL, Khalil T, Steele-Rosomoff R. Impact of chronic pain patients' job perception variables on actual return to work. Clin J Pain. 1997;13(3):197-206. Epub 1997/09/26. doi: 10.1097/00002508-199709000-00004. PubMed PMID: 9303251.

19. Franche R-L, Severin CN, Hogg-Johnson S, Côté P, Vidmar M, Lee H. The impact of early workplace-based return-to-work strategies on work absence duration: a 6-month longitudinal study following an occupational musculoskeletal injury. Journal of occupational and environmental medicine. 2007;49(9):960-74. doi: 10.1097/jom.0b013e31814b2e9f. PubMed PMID: 17848852.

20. Gaines WG, Jr., Hegmann KT. Effectiveness of Waddell's nonorganic signs in predicting a delayed return to regular work in patients experiencing acute occupational low back pain. Spine (Phila Pa 1976). 1999;24(4):396-400; discussion 1. Epub 1999/03/05. doi: 10.1097/00007632-199902150-00021. PubMed PMID: 10065525.

21. Gauthier N, Sullivan MJ, Adams H, Stanish WD, Thibault P. Investigating risk factors for chronicity: the importance of distinguishing between return-to-work status and self-report measures of disability. J Occup Environ Med. 2006;48(3):312-8. Epub 2006/03/15. doi: 10.1097/01.jom.0000184870.81120.49. PubMed PMID: 16531836.

22. Gross DP, Battié MC. Recovery expectations predict recovery in workers with back pain but not other musculoskeletal conditions. J Spinal Disord Tech. 2010;23(7):451-6. Epub 2010/04/24. doi: 10.1097/BSD.0b013e3181d1e633. PubMed PMID: 20414134.

23. Gross DP, Battié MC. Work-related recovery expectations and the prognosis of chronic low back pain within a workers' compensation setting. J Occup Environ Med. 2005;47(4):428-33. Epub 2005/04/13. doi: 10.1097/01.jom.0000158706.96994.a5. PubMed PMID: 15824635.

24. Grøvle L, Haugen AJ, Keller A, Ntvig B, Brox JI, Grotle M. Prognostic factors for return to work in patients with sciatica. Spine J. 2013;13(12):1849-57. Epub 2013/09/26. doi: 10.1016/j.spinee.2013.07.433. PubMed PMID: 24060231.

25. Gustafsson K, Lundh G, Svedberg P, Linder J, Alexanderson K, Marklund S. Psychological factors are related to return to work among long-term sickness absentees who have undergone a multidisciplinary medical assessment. J Rehabil Med. 2013;45(2):186-91. doi: 10.2340/16501977-1077. PubMed PMID: 23138390.

26. Hagen EM, Svensen E, Eriksen HR. Predictors and modifiers of treatment effect influencing sick leave in subacute low back pain patients. Spine (Phila Pa 1976). 2005;30(24):2717-23. PubMed PMID: 16371893.

27. Haldorsen EM, Indahl A, Ursin H. Patients with low back pain not returning to work. A 12-month follow-up study. Spine (Phila Pa 1976). 1998;23(11):1202-7; discussion 8. Epub 1998/06/24. doi: 10.1097/00007632-199806010-00004. PubMed PMID: 9636972.

28. Hara KW, Bjørngaard JH, Jacobsen HB, Borchgrevink PC, Johnsen R, Stiles TC, et al. Biopsychosocial predictors and trajectories of work participation after transdiagnostic occupational rehabilitation of participants with mental and somatic disorders: a cohort study. BMC Public Health. 2018;18(1):1014. doi: 10.1186/s12889-018-5803-0.

29. Haveraaen LA, Skarpaas LS, Aas RW. Job demands and decision control predicted return to work: the rapid-RTW cohort study. BMC Public Health. 2017;17(1):154. doi: 10.1186/s12889-016-3942-8.

30. Haveraaen LA, Skarpaas LS, Berg JE, Aas RW. Do psychological job demands, decision control and social support predictreturn to work three months after a return-to-work (RTW) programme? The rapid-RTW cohort study. Work. 2015;53 1:61-71.

31. Hedlund Å, Nilsson A, Boman E, Kristofferzon ML. Predictors of return to work and psychological well-being among women during/after long-term sick leave due to common mental disorders - a prospective cohort study based on the theory of planned behaviour. Health Soc Care Community. 2022;30(6):e5245-e58. Epub 2022/07/28. doi: 10.1111/hsc.13943. PubMed PMID: 35894151; PubMed Central PMCID: PMC10087653.

32. Hogg-Johnson S, Cole DC. Early prognostic factors for duration on temporary total benefits in the first year among workers with compensated occupational soft tissue injuries. Occup Environ Med. 2003;60(4):244-53. Epub 2003/03/28. doi: 10.1136/oem.60.4.244. PubMed PMID: 12660372; PubMed Central PMCID: PMC1740514.

33. Huijs JJ, Koppes LL, Taris TW, Blonk RW. Differences in predictors of return to work among long-term sick-listed employees with different self-reported reasons for sick leave. J Occup Rehabil. 2012;22(3):301-11. Epub 2012/02/04. doi: 10.1007/s10926-011-9351-z. PubMed PMID: 22302668.

34. Hunter SJ, Shaha S, Flint D, Tracy DM. Predicting return to work. A long-term follow-up study of railroad workers after low back injuries. Spine (Phila Pa 1976). 1998;23(21):2319-28. Epub 1998/11/20. doi: 10.1097/00007632-199811010-00014. PubMed PMID: 9820913.

35. Iles RA, Sheehan LR, Gosling CM. Assessment of a new tool to improve case manager identification of delayed return to work in the first two weeks of a workers' compensation claim. Clin Rehabil. 2020;34(5):656-66. Epub 2020/03/19. doi: 10.1177/0269215520911417. PubMed PMID: 32183561.

36. Jacobsen HB, Glette M, Hara KW, Stiles TC. Metacognitive Beliefs as Predictors of Return to Work After Intensive Return-to-Work Rehabilitation in Patients With Chronic Pain, Chronic Fatigue and Common Psychological Disorders: Results From a Prospective Trial. Front Psychol. 2020;11:70. Epub 2020/03/03. doi: 10.3389/fpsyg.2020.00070. PubMed PMID: 32116900; PubMed Central PMCID: PMC7025452.

37. Katz JN, Amick BC, 3rd, Keller R, Fossel AH, Ossman J, Soucie V, et al. Determinants of work absence following surgery for carpal tunnel syndrome. Am J Ind Med. 2005;47(2):120-30. Epub 2005/01/22. doi: 10.1002/ajim.20127. PubMed PMID: 15662641.

38. Koopman FS, Edelaar M, Slikker R, Reynders K, van der Woude LH, Hoozemans MJ. Effectiveness of a multidisciplinary occupational training program for chronic low back pain: a prospective cohort study. Am J Phys Med Rehabil. 2004;83(2):94-103. doi: 10.1097/01.PHM.0000107482.35803.11. PubMed PMID: 14758295.

39. Kvam L, Vik K, Eide AH. Importance of Participation in Major Life Areas Matters for Return to Work. Journal of Occupational Rehabilitation. 2015;25(2):368-77. doi: 10.1007/s10926-014-9545-2.

40. Lagerveld SE, Brenninkmeijer V, Blonk RW, Twisk J, Schaufeli WB. Predictive value of work-related self-efficacy change on RTW for employees with common mental disorders. Occup Environ Med. 2017;74(5):381-3. Epub 2016/12/23. doi: 10.1136/oemed-2016-104039. PubMed PMID: 28007760.

41. Lagerveld SE, Blonk RWB, Brenninkmeijer V, Schaufeli WB. Return to work among employees with mental health problems: Development and validation of a self-efficacy questionnaire. Work & Stress. 2010;24(4):359-75. doi: 10.1080/02678373.2010.532644.

42. Laukkala T, Heikinheimo S, Vuokko A, Junttila IS, Tuisku K. Subjective and objective measures of function and return to work: an observational study with a clinical psychiatric cohort. Soc Psychiatry Psychiatr Epidemiol. 2018;53(5):537-40. Epub 2017/12/25. doi: 10.1007/s00127-017-1479-5. PubMed PMID: 29275503.

43. Lepiece B, Reynaert C, Jacques D, Zdanowicz N. Returning to Work after a Common Mental Health Disorder: a New Preoccupation for Mental Health Professionals? Psychiatr Danub. 2017;29(Suppl 3):262-6. Epub 2017/09/28. PubMed PMID: 28953774.

44. Lötters F, Burdorf A. Prognostic factors for duration of sickness absence due to musculoskeletal disorders. Clin J Pain. 2006;22(2):212-21. Epub 2006/01/24. doi: 10.1097/01.ajp.0000154047.30155.72. PubMed PMID: 16428958.

45. Løvvik C, Shaw W, Overland S, Reme SE. Expectations and illness perceptions as predictors of benefit recipiency among workers with common mental disorders: secondary analysis from a randomised controlled trial. BMJ Open. 2014;4(3):e004321. Epub 2014/03/05. doi: 10.1136/bmjopen-2013-004321. PubMed PMID: 24589824; PubMed Central PMCID: PMC3948454.

46. Lydell M, Marklund B, Baigi A, Mattsson B, Mansson J. Return or no return--psychosocial factors related to sick leave in persons with musculoskeletal disorders: a prospective cohort study. Disabil Rehabil. 2011;33(8):661-6. doi: 10.3109/09638288.2010.506237. PubMed PMID: 20690859.

47. Lydell M, Grahn B, Månsson J, Baigi A, Marklund B. Predictive factors of sustained return to work for persons with musculoskeletal disorders who participated in rehabilitation. Work. 2009;33(3):317-28. Epub 2009/09/18. doi: 10.3233/wor-2009-0879. PubMed PMID: 19759430.

48. Momsen AM, Jensen OK, Nielsen CV, Jensen C. Multiple somatic symptoms in employees participating in a randomized controlled trial associated with sickness absence because of nonspecific low back pain. Spine J. 2014;14(12):2868-76. Epub 2014/04/20. doi: 10.1016/j.spinee.2014.01.062. PubMed PMID: 24743062.

49. Netterstrom B, Eller NH, Borritz M. Prognostic Factors of Returning to Work after Sick Leave due to Work-Related Common Mental Disorders: A One- and Three-Year Follow-Up Study. Biomed Res Int. 2015;2015:596572. doi: 10.1155/2015/596572. PubMed PMID: 26557678; PubMed Central PMCID: PMCPMC4628746.

50. Nicholas MK, Costa DSJ, Linton SJ, Main CJ, Shaw WS, Pearce R, et al. Predicting Return to Work in a Heterogeneous Sample of Recently Injured Workers Using the Brief ÖMPSQ-SF. J Occup Rehabil. 2019;29(2):295-302. Epub 2018/05/26. doi: 10.1007/s10926-018-9784-8. PubMed PMID: 29796980.

51. Nielsen MB, Bultmann U, Madsen IE, Martin M, Christensen U, Diderichsen F, et al. Health, work, and personal-related predictors of time to return to work among employees with mental health problems. Disabil Rehabil. 2012;34(15):1311-6. Epub 2011/12/28. doi: 10.3109/09638288.2011.641664. PubMed PMID: 22200251.

52. Nielsen MB, Madsen IE, Bultmann U, Christensen U, Diderichsen F, Rugulies R. Predictors of return to work in employees sick-listed with mental health problems: findings from a longitudinal study. Eur J Public Health. 2011;21(6):806-11. Epub 2010/12/04. doi: 10.1093/eurpub/ckq171. PubMed PMID: 21126986.

53. Nieuwenhuijsen K, Verbeek JH, de Boer AG, Blonk RW, van Dijk FJ. Predicting the duration of sickness absence for patients with common mental disorders in occupational health care. Scand J Work Environ Health. 2006;32(1):67-74. PubMed PMID: 16539174.

54. Nieuwenhuijsen K, Verbeek JH, de Boer AG, Blonk RW, van Dijk FJ. Validation of performance indicators for rehabilitation of workers with mental health problems. Med Care. 2005;43(10):1034-42.

55. Nieuwenhuijsen K, Verbeek JH, de Boer AG, Blonk RW, van Dijk FJ. Supervisory behaviour as a predictor of return to work in employees absent from work due to mental health problems. Occup Environ Med. 2004;61(10):817-23. Epub 2004/09/21. doi: 10.1136/oem.2003.009688. PubMed PMID: 15377767; PubMed Central PMCID: PMC1740675.

56. Norder G, Roelen CAM, van der Klink JJL, Bültmann U, Sluiter JK, Nieuwenhuijsen K. External Validation and Update of a Prediction Rule for the Duration of Sickness Absence Due to Common Mental Disorders. J Occup Rehabil. 2017;27(2):202-9. Epub 2016/06/05. doi: 10.1007/s10926-016-9646-1. PubMed PMID: 27260170; PubMed Central PMCID: PMC5405096.

57. Nordin M, Hiebert R, Pietrek M, Alexander M, Crane M, Lewis S. Association of comorbidity and outcome in episodes of nonspecific low back pain in occupational populations. J Occup Environ Med. 2002;44(7):677-84. Epub 2002/07/24. doi: 10.1097/00043764-200207000-00015. PubMed PMID: 12134532.

58. Okurowski L, Pransky G, Webster B, Shaw WS, Verma S. Prediction of prolonged work disability in occupational low-back pain based on nurse case management data. J Occup Environ Med. 2003;45(7):763-70. Epub 2003/07/12. doi: 10.1097/01.jom.0000079086.95532.e9. PubMed PMID: 12855916.

59. Opsahl J, Eriksen HR, Tveito TH. Do expectancies of return to work and Job satisfaction predict actual return to work in workers with long lasting LBP? BMC Musculoskelet Disord. 2016;17(1):481. Epub 2016/11/20. doi: 10.1186/s12891-016-1314-2. PubMed PMID: 27855684; PubMed Central PMCID: PMC5114779.

60. Oyeflaten I, Hysing M, Eriksen HR. Prognostic factors associated with return to work following multidisciplinary vocational rehabilitation. J Rehabil Med. 2008;40(7):548-54. doi: 10.2340/16501977-0202. PubMed PMID: 18758672.

61. Post M, Krol B, Groothoff JW. Self-rated health as a predictor of return to work among employees on long-term sickness absence. Disabil Rehabil. 2006;28(5):289-97. Epub 2006/02/24. doi: 10.1080/09638280500160303. PubMed PMID: 16492623.

62. Rashid M, Kristofferzon ML, Nilsson A. Predictors of return to work among women with long-term neck/shoulder and/or back pain: A 1-year prospective study. PLoS One. 2021;16(11):e0260490. Epub 2021/11/24. doi: 10.1371/journal.pone.0260490. PubMed PMID: 34813601; PubMed Central PMCID: PMC8610267.

63. Reme SE, Hagen EM, Eriksen HR. Expectations, perceptions, and physiotherapy predict prolonged sick leave in subacute low back pain. BMC Musculoskelet Disord. 2009;10:139. Epub 2009/11/17. doi: 10.1186/1471-2474-10-139. PubMed PMID: 19912626; PubMed Central PMCID: PMC2780378.

64. Sampere M, Gimeno D, Serra C, Plana M, López JC, Martínez JM, et al. Return to work expectations of workers on long-term non-work-related sick leave. J Occup Rehabil. 2012;22(1):15-26. Epub 2011/06/28. doi: 10.1007/s10926-011-9313-5. PubMed PMID: 21701951.

65. Schultz IZ, Crook J, Berkowitz J, Milner R, Meloche GR. Predicting return to work after low back injury using the Psychosocial Risk for Occupational Disability Instrument: a validation study. J Occup Rehabil. 2005;15(3):365-76. Epub 2005/08/27. doi: 10.1007/s10926-005-5943-9. PubMed PMID: 16119227.

66. Schultz IZ, Crook JM, Berkowitz J, Meloche GR, Milner R, Zuberbier OA, et al. Biopsychosocial multivariate predictive model of occupational low back disability. Spine (Phila Pa 1976). 2002;27(23):2720-5. Epub 2002/12/04. doi: 10.1097/00007632-200212010-00012. PubMed PMID: 12461399.

67. Schultz IZ, Crook J, Meloche GR, Berkowitz J, Milner R, Zuberbier OA, et al. Psychosocial factors predictive of occupational low back disability: towards development of a return-to-work model. Pain. 2004;107(1-2):77-85. Epub 2004/01/13. doi: 10.1016/j.pain.2003.09.019. PubMed PMID: 14715392.

68. Selander J, Marnetoft SU, Asell M. Predictors for successful vocational rehabilitation for clients with back pain problems. Disabil Rehabil. 2007;29(3):215-20. doi: 10.1080/09638280600756208. PubMed PMID: 17364772.

69. Skarpaas LS, Haveraaen LA, Småstuen MC, Shaw WS, Aas RW. The association between having a coordinator and return to work: the rapid-return-to-work cohort study. BMJ Open. 2019;9(2):e024597. doi: 10.1136/bmjopen-2018-024597.

70. Soucy I, Truchon M, Côté D. Work-related factors contributing to chronic disability in low back pain. Work. 2006;26(3):313-26. Epub 2006/05/25. PubMed PMID: 16720972.

71. Steenstra IA, Franche RL, Furlan AD, Amick B, 3rd, Hogg-Johnson S. The Added Value of Collecting Information on Pain Experience When Predicting Time on Benefits for Injured Workers with Back Pain. J Occup Rehabil. 2016;26(2):117-24. doi: 10.1007/s10926-015-9592-3. PubMed PMID: 26152837.

72. Steenstra IA, Koopman FS, Knol DL, Kat E, Bongers PM, de Vet HC, et al. Prognostic factors for duration of sick leave due to low-back pain in dutch health care professionals. J Occup Rehabil. 2005;15(4):591-605. doi: 10.1007/s10926-005-8037-9. PubMed PMID: 16254758.

73. Storheim K, Brox, J. I., Holm, I., & Bo, K. . Predictors of return to work in patients sick listed for sub-acute low back pain: a 12-month follow-up study. Journal of rehabilitation medicine. 2005;37(6):365-71. doi: <https://doi.org/10.1080/16501970510040344>.

74. Turner JA, Franklin G, Fulton-Kehoe D, Sheppard L, Wickizer TM, Wu R, et al. Worker recovery expectations and fear-avoidance predict work disability in a population-based workers' compensation back pain sample. Spine (Phila Pa 1976). 2006;31(6):682-9. Epub 2006/03/17. doi: 10.1097/01.brs.0000202762.88787.af. PubMed PMID: 16540874.

75. van Duijn M, Lotters F, Burdorf A. Influence of modified work on return to work for employees on sick leave due to musculoskeletal complaints. J Rehabil Med. 2005;37(3):172-9. doi: 10.1080/16501970410023434.

76. Victor M, Lau B, Ruud T. Predictors of Return to Work 6 Months After the End of Treatment in Patients with Common Mental Disorders: A Cohort Study. J Occup Rehabil. 2018;28(3):548-58. Epub 2017/12/14. doi: 10.1007/s10926-017-9747-5. PubMed PMID: 29234955; PubMed Central PMCID: PMC6096513.

77. Wåhlin C, Ekberg K, Persson J, Bernfort L, Oberg B. Association between clinical and work-related interventions and return-to-work for patients with musculoskeletal or mental disorders. J Rehabil Med. 2012;44(4):355-62. Epub 2012/03/22. doi: 10.2340/16501977-0951. PubMed PMID: 22434378.

78. Westman A, Linton SJ, Ohrvik J, Wahlen P, Leppert J. Do psychosocial factors predict disability and health at a 3-year follow-up for patients with non-acute musculoskeletal pain? A validation of the Orebro Musculoskeletal Pain Screening Questionnaire. Eur J Pain. 2008;12(5):641-9. doi: 10.1016/j.ejpain.2007.10.007. PubMed PMID: 18086541.
