# Supplementary material for: Systematic review and tools appraisal of prognostic factors of return to work in workers on sick leave due to musculoskeletal and common mental disorders
Source: PLoS One. 2024 Jul 17;19(7):e0307284. doi: 10.1371/journal.pone.0307284 (PMC11253986; doi:10.1371/journal.pone.0307284)
Supplement: S3 File — (DOCX) [file pone.0307284.s003.docx]

**Supplementary file 3**: Detailed evidentiary tables (prognostic factors of RTW)

**Table S2**. Evidentiary support for *Work accommodations (offer/availability/feasibility) -* Strong evidence for predicting RTW after a MSD

| **Number of studies and significant results^a^** | **Measurement tool used in each study showing a statistically significant predictive value**  **(Tools appraisal using psychometric (n = 6) and usability (n = 4) criteria is provided in Table 4)** | |
| --- | --- | --- |
|  | **Authors** | **Description, scoring and accessibility information** |
| 4 articles   1. Hogg-Johnson & Cole, 2003 [1] (+) 2. Franche et al., 2007 [2] (+) 3. Iles et al., 2020 [3] (+) 4. Turner et al., 2008 [4](+)   Level of evidence (all positive): STRONG | Hogg-Johnson & Cole, 2003[1] | **Tool 1 (T1)**  **Description:** This is a single question. Respondents were asked whether their workplace had made any offers of special arrangements to help them return to work.  **Scoring:** Yes/No  **Interpretation:** The availability of arrangements to help the person return to work is associated with more rapid termination of benefits.  **Accessibility:** Available in [5] |
|  | Franche et al., 2007[2] | **Tool 2 (T2)**  **Description:** Two items: 1) *Have you been offered work accommodation?* and 2) *Did you accept the work accommodation offer?*  **Scoring:** Yes/No  **Interpretation:** Work accommodation offer and acceptance are associated with a shorter work absence duration.  **Accessibility:** Available in [2] |
|  | Iles et al., 2020 [3] | **Tool 3 (T3)**  **Description:** One item (item S5) from the Plan of Action for a Case (PACE) tool: *Is the employer able to provide suitable duties?*  **Scoring:** Yes/No  **Interpretation:** No is considered as a high-risk response.  **Accessibility:** Available in [3, 6] |
|  | Turner et al., 2008 [4] | **Tool 4 (T4)**  **Description:** This is a single question. Respondents were asked if the employer offered job accommodation.  **Scoring:** Yes/No  **Interpretation:** No offer of a job accommodation (e.g., light duty) is associated with longer work disability.  **Accessibility:** Available in [4] |
|  | **Summary** | 2 studies used a single item  1 study used a single item from a standardized questionnaire  1 study used two items |

^a^ (+): statistically significant positive association; (-) statistically significant negative association; (NS) non statistically significant association. Rules to determine the level of evidence are explained in Fig. 2. Tools in S3 and S5 files have the same numbers (e.g., T4 correspond to the same tool in both files).

**Table S3.** Evidentiary support for *All work accommodations* *-* Strong evidence for predicting RTW after a MSD

| **Number of studies and significant results^a^** | **Measurement tool used in each study showing a statistically significant predictive value**  **(Tools appraisal using psychometric (n = 6) and usability (n = 4) criteria is provided in Table 4)** | |
| --- | --- | --- |
|  | **Authors** |  |
| 6 articles   1. Hogg-Johnson & Cole, 2003[1] (+) 2. Franche et al., 2007 [2] (+) 3. Franche et al., 2007 [2] (+) 4. Iles et al., 2020 [3](+) 5. Turner et al., 2008 [4] (+) 6. Steenstra et al., 2015 [7] (+)   Level of evidence (all positive): STRONG | Hogg-Johnson & Cole, 2003 [1] | ***Work accommodations (offer/availability/feasibility) -* Tool 1 (T1) as described in Table S2**  **Description:** This is a single question. Respondents were asked whether their workplace had made any offers of special arrangements to help them return to work.  **Scoring:** Yes/No  **Interpretation:** The availability of arrangements to help the person return to work is associated with more rapid termination of benefits.  **Accessibility:** Available in [5] |
|  | Franche et al., 2007 [2] | ***Work accommodations (offer/availability/feasibility) -* Tool 2 (T2) as described in Table S2**  **Description:** Two items: 1) *Have you been offered work accommodation?* and 2) *Did you accept the work accommodation offer?*  **Scoring:** Yes/No  **Interpretation:** Work accommodation offer and acceptance are associated with a shorter work absence duration.  **Accessibility:** Available in [2]  ***Work accommodations (worksite visit) -* Tool 5 (T5)**  **Description:** One item: 1) *Have you received an ergonomic assessment and recommendations at your workplace?*  **Scoring:** Yes/No  **Interpretation:** Work accommodation offer and acceptance are associated with a shorter work absence duration.  **Accessibility:** Available in [2] |
|  | Iles et al., 2020 [3] | ***Work accommodations (offer/availability/feasibility) -* Tool 3 (T3) as described in Table S2**  **Description:** One item (item S5) from the Plan of Action for a Case (PACE) tool: *Is the employer able to provide suitable duties?*  **Scoring:** Yes/No  **Interpretation:** No is considered as a high-risk response.  **Accessibility:** Available in [3, 6] |
|  | Turner et al., 2008 [4] | ***Work accommodations (offer/availability/feasibility) -* Tool 4 (T4) as described in Table S2**  **Description:** This is a single question. Respondents were asked if the employer offered job accommodation.  **Scoring:** Yes/No  **Interpretation:** No offer of a job accommodation (e.g., light duty) is associated with longer work disability.  **Accessibility:** Available in [4] |
|  | Steenstra et al., 2015 [7] | ***Work accommodations (RTW plan)***  **Description:** Health-care billings (Form 8)  **Scoring:** Not appropriate  **Interpretation:** When HCP discusses RTW, the employee is more likely to RTW  **Accessibility:** Unavailable |
|  | **Summary** | 2 studies used a single item  1 study used a single item from a standardized questionnaire  1 study used two items  1 study used a specific form used by the Ontario’s Workplace Safety and Insurance Board |

a (+): statistically significant positive association; (-) statistically significant negative association; (NS) non statistically significant association. Rules to determine the level of evidence are explained in Fig. 2. Tools in S3 and S5 files have the same numbers (e.g., T4 correspond to the same tool in both files).

**Table S4**. Evidentiary support for *Expectations (RTW)* – Strong evidence for predicting RTW after a MSD

| **Number of studies and significant results^a^** | **Measurement tool used in each study showing a statistically significant predictive value**  **(Tools appraisal using psychometric (n = 6) and usability (n = 4) criteria is provided in Table 4)** | |
| --- | --- | --- |
|  | **Authors** | **Description, scoring and accessibility information** |
| 14 articles   1. Reneman et al., 2021[8] (+) 2. Du Bois & Donceel, 2008 [9] (+) 3. Du Bois et al., 2009 [10] (+) 4. Fishbain et al., 1997 [11] (+) 5. Gross & Battié, 2005 [5] (+) 6. Gross & Battié, 2010 [12] (+) 7. Hara et al., 2018 [13] (+) 8. Nicholas et al., 2019 [14] (+) 9. Reme et al., 2009 [15] (+) 10. Sampere et al., 2012 [16] (+) 11. Steenstra et al., 2005 [17] (+) 12. Turner et al., 2006 [18] (+) 13. Turner et al., 2008 [4] (+) 14. Wåhlin et al., 2012 [19] (+)   Level of evidence (all positive): STRONG | Reneman et al., 2021 [8]  Turner et al., 2006 [18]  Turner et al., 2008 [4] | **Tool 6 (T6)**  **Description:** This is a single item. Respondents were asked to rate the certainty that they will be working in six months.  **Scoring:** 0-10 scale, from 0 (Not at all certain) to 10 (Extremely certain). The score is then dichotomized into negative RTW expectancy (score 0–5) and positive RTW expectancy (score 6–10)  **Interpretation:** Higher scores at this scale positively impacts RTW (increased work participation at 6 months) of workers with a MSD.  **Accessibility:** Available in [4, 8, 18] |
|  | Du Bois & Donceel, 2008 [9]  Du Bois et al., 2009 [10] | **Tool 7 (T7)**  **Description:** One item (item 16) from the Örebro Musculoskeletal Pain questionnaire: *Do you expect to return to work within 6 months?*  **Scoring:** 0-10 scale, from 1 (no chance) to 10 (very large chance)  **Interpretation:** Higher scores at this scale positively impacts RTW (reduce sickness absence duration) of workers with a MSD.  **Accessibility:** Available in [10, 20] |
|  | Fishbain et al, 1997 [11] | **Tool 8 (T8)**  **Description:** This is a single item in the wording: *Are you planning to return to your preinjury type of work if you are rehabilitated?*  **Scoring:** Possible answers are: Yes, No, Not sure, Not applicable  **Interpretation:** Plans to return to the preinjury type of work if rehabilitated positively impacts RTW (reduce sickness absence duration) of a worker with a MSD.  **Accessibility:** Available in [11] |
|  | Gross & Battié, 2005 [5]  Gross & Battié, 2010 [12] | **Tool 9 (T9)**  **Description:** Standardized questionnaire entitled “Work-Related Recovery Expectations Questionnaire” (3 items).  **Scoring:** five-point Likert scale (1 = strongly disagree, 5 = strongly agree).  **Interpretation:** Higher scores at this scale positively impacts RTW (reduce sickness absence duration) of workers with a MSD.  **Accessibility:** Available in [5, 12] |
|  | Hara et al, 2018 [13] | **Tool 10 (T10)**  **Description:** One item from the Fear Avoidance Beliefs Questionnaire (FABQ), work subscale: *I do not think that I will be back in my ordinary work within 3 months*  **Scoring:** seven-point Likert scale (0= completely disagree, 6= completely agree)  **Interpretation:** Higher score at this scale indicates a worse fear of not returning to work. The cut off is set at ≥3 indicating a non-positive expectation defined as an “uncertain or even poorer expectation of RTW  **Accessibility:** Available in [13, 21] |
|  | Nicholas et al, 2019 [14] | **Tool 11 (T11)**  **Description:** One item (item 8) from the standardized questionnaire entitled Örebro Musculoskeletal Pain Screening Questionnaire- short form (ÖMPSQ-SF) : *In your estimation, what are the chances you will be working your normal duties in 3 months?*  **Scoring:** 0-10 Likert scale, from 0 (no risk) to 10 (very large risk)  **Interpretation:** Higher scores at this scale positively impacts RTW (reduce sickness absence duration) of workers with a MSD.  **Accessibility:** Available in [22] |
|  | Reme et al, 2009 [15] | **Tool 12 (T12)**  **Description:** This is a single item. Respondents are asked whether they expect to return to work within the next few weeks or not.  **Scoring:** Yes/No  **Interpretation:** Positive expectations stemming from agreeing with the statement, positively impacts RTW (reduce sickness absence duration) of workers with a MSD.  **Accessibility:** Available in [15] |
|  | Sampere et al, 2012 [16] | **Tool 13 (T13)**  **Description:** This is a single item in the wording: *Approximately how long do you think you will need to return to the job you had before you went on sick leave? (we understand that this question is difficult to answer, please try to give an answer, even if it is only approximate)*  **Scoring:** Possible answers are ‘Less than 1 week’, ‘Between 1 and 4 weeks’, ‘Between 1 and 3 months’, ‘Between 4 and 6 months’, ‘Over 6 months’, ‘I will never be able to perform the job I used to before’, ‘I do not know’, ‘I have no idea how long I will take to recover’. Response options are then collapsed into <1, 1–3, >3 months, I will never be and I don’t know.  **Interpretation:** Time (lower) estimated by the worker to be able to perform the same job is related to a quicker time to RTW.  **Accessibility:** Available in [16] |
|  | Steenstra et al, 2005 [17] | **Tool 14 (T14)**  **Description:** Single item about the expected duration of sick leave.  **Scoring:** from 1 to 10 days or 10 days or more  **Interpretation:** Lower scores at this scale positively impacts RTW (reduce sickness absence duration) of workers with a MSD.  **Accessibility:** Available in [17] |
|  | Wåhlin et al, 2012 [19] | **Tool 15 (T15)**  **Description:** This is a single item in the wording: *In your estimation, what are the chances that you will be working in 6 months?*  **Scoring:** 5-point scale, where 1 was “very good chance” and 5 was “very little chance”, dichotomized in high and low  **Interpretation:** Lower scores at this scale positively impacts RTW (reduce sickness absence duration) of workers with a MSD.  **Accessibility:** Available in [19] |
|  | **Summary** | 8 studies used a single item (ah hoc)  4 studies used a single item from a standardized questionnaire  2 studies used a standardized questionnaire |

^a^ (+): statistically significant positive association; (-) statistically significant negative association; (NS) non statistically significant association. Rules to determine the level of evidence are explained in Fig. 2. Tools in S3 and S5 files have the same numbers (e.g., T4 correspond to the same tool in both files).

**Table S5.** Evidentiary support for *Fear (FABQ-W)* – Strong evidence for predicting RTW after a MSD

| **Number of studies and significant results *** | **Measurement tool used in each study showing a statistically significant predictive value**  **(Tools appraisal using psychometric (n = 6) and usability (n = 4) criteria is provided in Table 4)** | |
| --- | --- | --- |
|  | **Authors** | **Description, scoring and accessibility information** |
| 7 articles   1. Grøvle et al., 2013 [23] (-) 2. Opsahl et al., 2016 [24] (-) 3. Oyeflaten et al., 2008 [25] (-) 4. Soucy et al., 2006 [26](-) 5. Storheim et al., 2005 [27] (-) 6. Turner et al., 2006 [18] (-) 7. Turner et al., 2008 [4] (-)   Level of evidence (all negative): STRONG | Grøvle et al., 2013 [23]  Opsahl et al., 2016 [24]  Oyeflaten et al., 2008 [25]  Soucy et al., (2006) [26]  Storheim et al., 2005 [27] | **Tool 16 (T16)**  **Description:** Work subscale (7 items) of the Fear-Avoidance Beliefs Questionnaire (FABQ)  **Scoring:** 7-point scale: Completely disagree = 0, completely agree = 6; Subscale score range: 0-42  **Interpretation:** Higher scores on this subscale means more fear-avoidance of work activities, which negatively impacts RTW (increase sickness absence duration) of workers with a MSD.  **Accessibility:** Available in [21] |
|  | Turner et al., 2006 [18]  Turner et al., 2008 [4] | **Tool 17 (T17)**  **Description:** Two items (my work might harm my back; my work makes, or might make, my pain worse) from the Work subscale (7 items) of the Fear-Avoidance Beliefs Questionnaire (FABQ)  **Scoring:** 7-point scale: Completely disagree = 0, completely agree = 6; Subscale score range (mean): 0-6  **Interpretation:** Higher scores on these items means more fear-avoidance of work activities, which negatively impacts RTW (increase sickness absence duration) of workers with a MSD.  **Accessibility:** Available in [21] |
|  | **Summary** | 4 studies used a subscale of a standardized questionnaire  2 studies used two items of a subscale of a standardized questionnaire  1 study used a French version of the same subscale |

^a^ (+): statistically significant positive association; (-) statistically significant negative association; (NS) non statistically significant association. Rules to determine the level of evidence are explained in Fig. 2. Tools in S3 and S5 files have the same numbers (e.g., T4 correspond to the same tool in both files).

**Table S6.** Evidentiary support for *All coping strategies factors* – Moderate evidence for predicting RTW after a MSD

| **Number of studies and significant results** ^a^ | **Measurement tool used in each study showing a statistically significant predictive value**  **(Tools appraisal using psychometric (n = 6) and usability (n = 4) criteria is provided in Table 4)** | |
| --- | --- | --- |
|  | **Authors** | **Description, scoring and accessibility information** |
| 3 articles   1. Koopman et al. (2004)[28] (-) 2. Truchon & Côté (2005) [29] (-) 3. Rashid et al. (2021) [30] (-)   Level of evidence (all negative): STRONG | Koopman et al. (2004) [28] | **Tool 18 (T18)**  **Description:** The “Reinterpretations of pain sensations” subscale (6 items) of the Dutch version of the Coping Strategy Questionnaire measures some coping cognitive strategies.  **Scoring:** 7-point Likert scale, from “never” = 0 to “sometimes” = 3 and “always” = 6 (subscale score range (mean of items): 0-6).  **Interpretation:** Lower scores on this subscale means less pain coping in terms of reinterpretations of pain sensations, which negatively impacts RTW (increase sickness absence duration) of workers with a MSD.  **Accessibility:** There is a copyright held by Anne Rosenstiel Gross, but apparently no website to obtain the tool. |
|  | Truchon & Côté (2005) [29] | **Tool 19 (T19)**  **Description:** The “guarding” (9 items) subscale of the Chronic Pain Coping Inventory measures some behavioural coping strategies. The “exercise/stretch” subscale (12 items), which also measures behavioural coping strategies, was also predictive of RTW, but represents a protective factor (+) instead of a risk factor (-). However, although both scales can be used independently, Truchon and Côté showed that the “guarding” subscale was much more predictive of RTW than the “exercise/stretch” subscale.  **Scoring:** A French version was used in this article, using a 4-point Likert scale, from “never” = 0, “sometimes” = 1, “often” = 2, “very often” = 3 (subscale score range – (mean of items): 0-3). However, this is different from the original English 8-category scale format as shown in the generic question that can be applied to all strategies (items): *During the past week, how many days (0 to 7) did you use this strategy* (subscale score range (mean of items): 0-6)  **Interpretation:** Higher scores on the “guarding” subscale is considered as a maladaptive pain coping strategy, which negatively impacts RTW (increase sickness absence duration) of workers with a MSD.  **Accessibility:** The original scale (English version) is available online (fees): <https://www.parinc.com/Products/Pkey/66> |
|  | Rashid et al. (2021) [30] | **Tool 20 (T20)**  **Description:** The “Increasing pain behaviour” (6 items) subscale of the Swedish version of the Coping Strategy Questionnaire measures some coping behavioural strategies.  **Scoring:** 7-point Likert scale, from “never” = 0 to “sometimes” = 3 and “always” = 6 (subscale score range (mean of items): 0-6).  **Interpretation:** Lower scores on this subscale means less pain coping in terms of increasing pain behaviour, which negatively impacts RTW (increase sickness absence duration) of workers with a MSD.  **Accessibility:** There is a copyright held by Anne Rosenstiel Gross, but apparently no website to obtain the tool. |
|  | **Summary** | 2 studies used a subscale of a standardized questionnaire  1 study used a subscale of a modified version of a standardized questionnaire |

^a^ (+): statistically significant positive association; (-) statistically significant negative association; (NS) non statistically significant association. Rules to determine the level of evidence are explained in Fig. 2. Tools in S3 and S5 files have the same numbers (e.g., T4 correspond to the same tool in both files).

**Table S7.** Evidentiary support for *Expectations (recovery)* – Strong evidence for predicting RTW after a MSD

| **Number of studies and significant results** ^a^ | **Measurement tool used in each study showing a statistically significant predictive value**  **(Tools appraisal using psychometric (n = 6) and usability (n = 4) criteria is provided in Table 4)** | |
| --- | --- | --- |
|  | **Authors** | **Description, scoring and accessibility information** |
| 6 articles   1. Cole et al. (2002) [31] (+) 2. Hagen et al. (2005) [32] (+) 3. Hogg-Johnson & Cole (2003) [1] (+) 4. Schultz et al. (2004) [33] (+) 5. Schultz et al. (2005) [34] (+) 6. Nicholas et al. (2019) [14] (+)   Level of evidence (all positive): STRONG | Cole et al. (2002) [31] | **Tool 21 (T21)**  **Description:** Non-standardized questionnaire (3 out of 4 items were predictive: namely items 1, 2 and 4).  **Scoring:** There is no score calculation.  **Interpretation:** Positive recovery expectations positively impacts RTW (reduce sickness absence duration) of workers with a MSD.  **Accessibility:** Available in Cole et al. (2002) [31] |
|  | Hagen et al. (2005) [32] | **Tool 22 (T22)**  **Description:** The exact wording of the question is unknown but here is what was reported: *Belief that their back pain won’t disappear?*  **Scoring:** The 3-point scale was found in a review on recovery expectations tools [35]: ‘‘to a small extent’’, ‘‘some extent’’, and large extent’’, dichotomised into “to a small extent” and “some and large extent”  **Interpretation:** Having no beliefs (or to a small extent) that back pain won’t disappear positively impacts RTW (reduce sickness absence duration) of workers with a MSD.  **Accessibility:** Available in Hagen et al. (2005) [32] but more complete information provided here. |
|  | Hogg-Johnson & Cole (2003) [1] | **Tool 23 (T23)**  **Description:** The exact wording of the question is unknown (*When recover?* [1]) but it was reported in a review on recovery expectations tools [35]: *When s/he thinks s/he will recover?*  **Scoring:** Soon; Slowly; Get worse; Recur. Dichotomised into think they recover soon versus do not think they will recover soon  **Interpretation:** Positive recovery expectations (soon) positively impacts RTW (reduce sickness absence duration) of workers with a MSD.  **Accessibility:** Available in Hogg-Johnson & Cole (2003) [1] and Ebrahim et al. (2015) [35] |
|  | Schultz et al. (2004) [33]  Schultz et al. (2005) [34] | **Tool 24-25 (T24-25)**  **Description:** Expectations of Recovery scale (non-standardized questionnaire; 7 items; T7) based on [31] (4 items) and [36] (3 items). Schultz et al. (2004) [33] used 6 of these 7 questions (T8) as 3 out of 4 items were predictive (items 1, 2 and 4) in [33].  **Scoring:** Unknown score calculation.  **Interpretation:** Positive recovery expectations positively impacts RTW (reduce sickness absence duration) of workers with a MSD.  **Accessibility:** Available in [33] (4 items) and (3 items) [36] |
|  | Nicholas et al. (2019) [14] | **Tool 26 (T26)**  **Description:** One item (item 7 on expecting persisting pain) from the standardized questionnaire Örebro Musculoskeletal Pain Screening Questionnaire- short form (ÖMPSQ-SF) [22]: *In your view, how large is the risk that your current pain may become persistent?*  **Scoring:** 0-10 scale from 0 (no risk) to 10 (very large risk)  **Interpretation:** Positive recovery expectations, namely lower scores on this scale, positively impacts RTW (reduce sickness absence duration) of workers with a MSD.  **Accessibility:** Available in [22] |
|  | **Summary** | 2 studies used a non-standardized questionnaire  3 studies used single items  1 study used a single item from a standardized questionnaire |

^a^ (+): statistically significant positive association; (-) statistically significant negative association; (NS) non statistically significant association. Rules to determine the level of evidence are explained in Fig. 2. Tools in S3 and S5 files have the same numbers (e.g., T4 correspond to the same tool in both files).

**Table S8.** Evidentiary support for *Locus of control* – Strong evidence for predicting RTW after a MSD

| **Number of studies and significant results *** | **Measurement tool used in each study showing a statistically significant predictive value** | |
| --- | --- | --- |
|  | **Authors** | **Description, scoring and accessibility information** |
| 3 articles   1. Hagen et al. (2005) [32](+) 2. Haldorsen et al. (1998) [37](+) 3. Selander et al. (2007) [38] (+)   Level of evidence (all positive): STRONG | Hagen et al. (2005) [32] | **Tool 27 (T27)**  **Description:** Subscale Chance externality (6 items) of the Multidimensional Health Locus of Control questionnaire (18 items) – Form A.  **Scoring:** 6-point Likert scale, from “strongly disagree = 1” to “strongly agree = 6” (6-36).  **Interpretation:** Higher scores on this scale means that the worker believe that health problems are due to chance and luck (not to their work), which positively impacts RTW (reduce sickness absence duration) of workers with a MSD.  **Accessibility:** <https://nursing.vanderbilt.edu/projects/wallstonk/index.php> |
|  | Haldorsen et al. (1998) [37] | **Tool 28 (T28)**  **Description:** Subscale Internality (6 items) of the Multidimensional Health Locus of Control questionnaire (18 items) - Form A.  **Scoring:** 6-point Likert scale, from “strongly disagree = 1” to “strongly agree = 6” (6-36).  **Interpretation:** Higher scores on this scale means that the worker believes that power to affect his state of health lies within his own control, which positively impacts RTW (reduce sickness absence duration) of workers with a MSD.  **Accessibility:** <https://nursing.vanderbilt.edu/projects/wallstonk/index.php> |
|  | Selander et al. (2007) [38] | **Tool 29 (T29)**  **Description:** Subscale Internal locus of control (3 items) of the modified version of Wallston’s Health Locus of Control scale (9 items), with modification of the wording to specify RTW as the salient achievement.  **Scoring:** 6-point Likert scale, from “strongly disagree = 1” to “strongly agree = 6” (3-18).  **Interpretation:** Higher scores on this scale means that the worker believes that power to affect his RTW lies within his own control, which positively impacts RTW (reduce sickness absence duration) of workers with a MSD.  **Accessibility:** Available in Selander et al. (2007) [38] |
|  | **Summary** | 2 studies used a subscale of a standardized questionnaire  1 study used a subscale of a modified version of a standardized questionnaire |

^a^ (+): statistically significant positive association; (-) statistically significant negative association; (NS) non statistically significant association. Rules to determine the level of evidence are explained in Fig. 2. Tools in S3 and S5 files have the same numbers (e.g., T4 correspond to the same tool in both files).

**Table S9.** Evidentiary support for *Job demands (physical)* – Moderate evidence for predicting RTW after a MSD

| **Number of studies and significant results^a^** | **Measurement tool used in each study showing a statistically significant predictive value**  **(Tools appraisal using psychometric (n = 6) and usability (n = 4) criteria is provided in Table 4)** | |
| --- | --- | --- |
|  | **Authors** | **Description, scoring and accessibility information** |
| 11 articles   1. Bosman et al., 2019 [39] (-) 2. Hansson & Hansson, 2000-DNK [40](-) 3. Hansson & Hansson, 2000-GER [40] (-) 4. Hansson & Hansson, 2000-ISR [40] (-) 5. Hansson & Hansson, 2000-NDL [40] (-) 6. Hansson & Hansson, 2000-SWE [40] (-) 7. Hansson & Hansson, 2000-US [40] (-) 8. Hara et al., 2018 [13] (-) 9. Lötters & Burdorf, 2006 [41] (-) 10. Reme et al., 2009 [15] (-) 11. Steenstra et al., 2015 [7] (-) 12. Amick et al., 2017 [42](NS) 13. Huijs et al., 2012 [43] (NS) 14. Storheim et al., 2005 [27] (NS) 15. Abásolo et al., 2008[44] (NS) 16. Gaines et al., 1999 [45](NS)   Level of evidence (all positive): MODERATE | Bosman et al., 2019 [39] | **Tool 30 (T30)**  **Description:** Dutch Musculoskeletal Questionnaire, 17 items  **Scoring:** Answer options ranged from 0 (never) to 3 (very frequently) and resulted in a mean score ranging between 0 and 3.  **Interpretation:** Higher scores at this scale negatively impacts RTW (increase sickness absence duration) of workers with a MSD.  **Accessibility:** The full version (63 items) is available in [46] but we were unable to find (after trying to contact the authors) which 17 items were selected for this abridged version. |
|  | Hansson & Hansson, 2000 [40] | **Tool 31 (T31)**  **Description:** Three ad-hoc items about physical demands (working in twisted positions, working in the same position during a prolonged time, heavy lifts)  **Scoring:**  Answer options ranged from 1 (high physical demands) to 4 (low physical demands) and resulted in a mean score ranging between 1 and 4  **Interpretation:** Higher scores at this scale negatively impacts RTW (increase sickness absence duration) of workers with a MSD.  **Accessibility:** Available in [40]. |
|  | Hara et al., 2018 [13] | **Tool 32 (T32)**  **Description:** One item from the Psychosocial Assessment Instrument (PAI): *Is your work very physically demanding?*  **Scoring:** The response alternatives are “not at all, a little, quite a bit, much”. A cut-off was set at ≥ “quite a bit”.  **Interpretation:** Higher scores at this scale negatively impacts RTW (increase sickness absence duration) of workers with a MSD.  **Accessibility:** Available in [13] |
|  | Lötters & Burdorf, 2006 [41] | **Tool 33 (T33)**  **Description:** 7 items from the Dutch Musculoskeletal Questionnaire: manual material handling, frequent bending twisting of the trunk, whole body vibration, working in awkward postures, working in static postures, and strenuous work with neck/shoulder and the upper limb.  **Scoring:** 4-point scale, from 0 (never) to 3 (always). A sum score across items was calculated indicating that the higher the score, the more physical risk factors were present.  **Interpretation:** Higher scores at this scale negatively impacts RTW (increase sickness absence duration) of workers with a MSD.  **Accessibility:** The abridged 7 items (see description above) are available in [41] and the full version (63 items) is available in [46] but we haven't been able to bridge the gap between the two as we have not received responses from the authors. |
|  | Reme et al., 2009 [15] | **Tool 34 (T34)**  **Description:** This is a single item in the wording: *Do you work in positions involving constant strain to the back?*  **Scoring:**  The original scale was as follows: 1=almost all the time, 2=about ¾ of the time, 3=about half the time, 4=about ¼ of the time, 5=very little, 6=no/never. Then, the score was dichotomized as follows: more than half of the time (scores 1-3) and less than half of the time (scores 4-6).  **Interpretation:** Having constant back strain more than half of the time negatively impacts RTW (increase sickness absence duration) of workers with a MSD.  **Accessibility:** The first author provided the above description and scoring detailed information. |
|  | Steenstra et al., 2015 [7] | **Tool 35 (T35)**  **Description:** Workplace physical demands were classified based on the National Occupational Code (Electronic claim file) as manual (high physical demands), mixed or non-manual work  **Scoring:** manual (high physical demands), mixed or non-manual work  **Interpretation:** High physical demands negatively impacts RTW (increase sickness absence duration) of workers with a MSD.  **Accessibility:** Unavailable |
|  | **Summary** | 2 studies used multiple items from a standardized questionnaire  1 study (counting for 6 studies in one article) used a subscale of a standardized questionnaire  1 study used one item from a standardized questionnaire  1 study used ad-hoc item  1 study used electronic claim file |

^a^ (+): statistically significant positive association; (-) statistically significant negative association; (NS) non statistically significant association. Rules to determine the level of evidence are explained in Fig. 2. Tools in S3 and S5 files have the same numbers (e.g., T4 correspond to the same tool in both files).

**Table S10**. Evidentiary support for *Job strain* – Moderate evidence for predicting RTW after a MSD

| **Number of studies and significant results *** | **Measurement tool used in each study showing a statistically significant predictive value**  **(Tools appraisal using psychometric (n = 6) and usability (n = 4) criteria is provided in Table 4)** | |
| --- | --- | --- |
|  | **Authors** | **Description, scoring and accessibility information** |
| 3 articles   1. Haveraaen et al., 2016 [47](-) 2. Haveraaen et al., 2017 [48] (-) 3. Soucy et al., 2006 [26](-)   Level of evidence (all negative): MODERATE | Haveraaen et al., 2016 [47]  Haveraaen et al., 2017 [48]  Soucy et al., 2006 [26] | **Tool 36 (T36)**  **Description:** Two subscales of the Job Content Questionnaire (5 items for Job demands and 9 items for decision latitude)  **Scoring:** 4-point Likert scale, ‘strongly disagree’ to ‘strongly agree’.  **Interpretation:** High levels of job strain negatively impacts RTW (increase sickness absence duration) of workers with a MSD.  **Accessibility:** This questionnaire is copyright protected. Requests should be made to the JCQ Center in Denmark by telephone (+45 40461000) or e-mail (jcqcenter@oresundsynergy.com). Access is free in the vast majority of cases, but a fee may be charged for research and commercial projects. |
|  | **Summary** | 3 studies used two subscales of a standardized questionnaire |

^a^ (+): statistically significant positive association; (-) statistically significant negative association; (NS) non statistically significant association. Rules to determine the level of evidence are explained in Fig. 2. Tools in S3 and S5 files have the same numbers (e.g., T4 correspond to the same tool in both files).

**Table S11.** Evidentiary support for *Work ability* – Moderate evidence for predicting RTW after a MSD

| **Number of studies and significant results^a^** | **Measurement tool used in each study showing a statistically significant predictive value**  **(Tools appraisal using psychometric (n = 6) and usability (n = 4) criteria is provided in Table 4)** | |
| --- | --- | --- |
|  | **Authors** | **Description, scoring and accessibility information** |
| 3 articles   1. Haldorsen et al., 1998 [37] (+) 2. Sampere et al., 2012 [16](+) 3. Wåhlin et al., 2012 (+)   Level of evidence (all positive): MODERATE | Haldorsen et al., 1998 [37] | **Tool 37 (T37)**  **Description:** Graded Reduced Work Ability scale (5 items)  **Scoring:** 5-point scale, score range 5-25, lower scores indicate greater work ability; A great deal = 1; A lot = 2; Some = 3; Not much = 4; Very little = 5  **Interpretation:** Higher scores on this questionnaire is positively associated with RTW (reduce sickness absence duration) of workers with a MSD.  **Accessibility:** Available in [37] but the score was described in [49]. |
|  | Sampere et al., 2012 [16] | **Tool 38 (T38)**  **Description:** One item developed from the Graded Reduced Work Ability scale: *To what degree is your (the patient’ s) ability to perform your (his or her) ordinary, remunerative work reduced today?*  **Scoring:** numerical scale from 0 to 10; values were recorded into three categories: not at all or slightly reduced work ability (from 0 to 3); moderately reduced (from 4 to 6), and very or extremely reduced (from 7 to 10)  **Interpretation:** Lower scores on this item is positively associated with RTW (reduce sickness absence duration) of workers with a MSD.  **Accessibility:** Available in [16, 50] |
|  | Wåhlin et al., 2012 [19] | **Tool 39 (T39)**  **Description:** One item from the standardized Work Ability Index (10 items): *current work ability compared with the lifetime best*  **Scoring:** The score is graded from 0 to 10, where 0 stands for completely unable to work and 10 stands for work ability at its best  **Interpretation:** Higher scores on this questionnaire is positively associated with RTW (reduce sickness absence duration) of workers with a MSD.  **Accessibility:** Available in [19, 51] |
|  | **Summary** | 1 study used a standardized questionnaire  1 study used one item of a standardized questionnaire  1 study used a single item adapted from a standardized questionnaire |

^a^ (+): statistically significant positive association; (-) statistically significant negative association; (NS) non statistically significant association. Rules to determine the level of evidence are explained in Fig. 2. Tools in S3 and S5 files have the same numbers (e.g., T4 correspond to the same tool in both files).

**Table S12.** Evidentiary support for *Self-efficacy (RTW)* – Moderate evidence for predicting RTW after a MSD

| **Number of studies and significant results^a^** | **Measurement tool used in each study showing a statistically significant predictive value**  **(Tools appraisal using psychometric (n = 6) and usability (n = 4) criteria is provided in Table 4)** | |
| --- | --- | --- |
|  | **Authors** | **Description, scoring and accessibility information** |
| 2 articles   1. Corbière et al., 2017[52] (+) 2. Huijs et al., 2012 [43](+)   Level of evidence (all positive): MODERATE | Corbière et al., 2017 [52] | **Tool 40 (T40)**  **Description:** Return-to-Work Obstacles and Self-Efficacy Scale (ROSES)  **Scoring:** The ROSES questionnaire is composed of two parts: perceived obstacles to RTW (Part A) and self-efficacy beliefs about overcoming them (Part B). First, the participant is asked to answer the following question in Part A: ‘‘Do you see the item below [97 items in total] as an obstacle to your return to work?’’ The answer scale is a seven-point Likert-type rating scale (1 = Not an obstacle, 7 = Big obstacle). Only if the participant selects an answer rated greater than 1 (i.e., the item represents a potential obstacle to the RTW) is he or she invited to answer the corresponding question in Part B: ‘‘How capable do you feel of overcoming this obstacle?’’ Again the answer scale is a seven-point Likert-type rating scale (1 = Not at all capable, 7 = Completely capable).  **Interpretation:** Higher scores on this questionnaire is positively associated with RTW (reduce sickness absence duration) of workers with a MSD.  **Accessibility:** Available in [52] |
|  | Huijs et al., 2012 [43] | **Tool 41 (T41)**  **Description:** Return to work self-efficacy questionnaire (11 items)  **Scoring:** 6-point scale, from 0 (disagree entirely) to 5 (agree entirely)  **Interpretation:** Higher scores on this questionnaire is positively associated with RTW (reduce sickness absence duration) of workers with a MSD.  **Accessibility:** Available in [53] |
|  | **Summary** | 2 studies used a standardized questionnaire |

^a^ (+): statistically significant positive association; (-) statistically significant negative association; (NS) non statistically significant association. Rules to determine the level of evidence are explained in Fig. 2. Tools in S3 and S5 files have the same numbers (e.g., T4 correspond to the same tool in both files).

**Table S13.** Evidentiary support for *Referred pain (back pain)* – Moderate evidence for predicting RTW after a MSD (low back pain)

| **Number of studies and significant results** ^a^ | **Measurement tool used in each study showing a statistically significant predictive value**  **(Tools appraisal using psychometric (n = 6) and usability (n = 4) criteria is provided in Table 4)** | |
| --- | --- | --- |
|  | **Authors** | **Description, scoring and accessibility information** |
| 4 articles   1. Du Bois & Donceel (2008) [9] (-) 2. Schultz et al. (2002) [54] (-) 3. Turner et al. (2008) [4] (-) 4. Grøvle et al. (2013) [23] (NS)   Level of evidence (majority negative): MODERATE | Du Bois & Donceel (2008) [9] | **Tool 42 (T42)**  **Description:** This is a single question: *Where did you have pain last week?*  **Scoring:** Back or above knee only: 0; Below knee also:2  **Interpretation:** Having pain below the knee negatively impacts RTW (increase sickness absence duration) of workers with low back pain.  **Accessibility:** Available in [9] |
|  | Schultz et al. (2002) [54] | **Tool 43 (T43)**  **Description:** Left and right leg typical sciatica from the physical examination.  **Scoring:** Yes/No  **Interpretation:** Having left or right sciatica negatively impacts RTW (increase sickness absence duration) of workers with low back pain.  **Accessibility:** This is a routine clinical test in patients with low back pain or sciatica. |
|  | Turner et al. (2008) [4] | **Tool 44 (T44)**  **Description:** Evidence of radiculopathy from the physical examination  **Scoring:** Yes/No  **Interpretation:** Observing some evidence of radiculopathy negatively impacts RTW (increase sickness absence duration) of workers with low back pain.  **Accessibility:** This is a routine clinical test in patients with low back pain or sciatica. |
|  | **Summary** | 1 study used a single question  2 studies used clinical tests |

^a^ (+): statistically significant positive association; (-) statistically significant negative association; (NS) non statistically significant association. Rules to determine the level of evidence are explained in Fig. 2. Tools in S3 and S5 files have the same numbers (e.g., T4 correspond to the same tool in both files).

**Table S14.** Evidentiary support for *Activities (disability/ODI)* – Moderate evidence for predicting RTW after a MSD (low back pain)

| **Number of studies and significant results** ^a^ | **Measurement tool used in each study showing a statistically significant predictive value**  **(Tools appraisal using psychometric (n = 6) and usability (n = 4) criteria is provided in Table 4)** | |
| --- | --- | --- |
|  | **Authors** | **Description, scoring and accessibility information** |
| 2 articles   1. Asher et al. (2017) [55] (-) 2. Opsahl et al. (2016) [24](-)   Level of evidence (all negative): MODERATE | Asher et al. (2017) [55]  Opsahl et al. (2016) [24] | **Tool 45 (T45)**  **Description:** Standardized questionnaire Oswestry disability index (10 items), version 2.0 [56, 57]  **Scoring:** Each items has different response options scored on a 6-point Likert scale, from “no limitation” = 1 to “maximal limitation” = 5 (total score range: 0-100)  **Interpretation:** Higher scores on this questionnaire means higher perceived disability, which negatively impacts RTW (increase sickness absence duration) of workers with a MSD.  **Accessibility:** Available in [57] |
|  | **Summary** | 2 studies used the same standardized questionnaire |

^a^ (+): statistically significant positive association; (-) statistically significant negative association; (NS) non statistically significant association. Rules to determine the level of evidence are explained in Fig. 2. Tools in S3 and S5 files have the same numbers (e.g., T4 correspond to the same tool in both files).

**Table S15.** Evidentiary support for *Activities (disability/SF36)* – Moderate evidence for predicting RTW after a MSD

| **Number of studies and significant results** ^a^ | **Measurement tool used in each study showing a statistically significant predictive value**  **(Tools appraisal using psychometric (n = 6) and usability (n = 4) criteria is provided in Table 4)** | |
| --- | --- | --- |
|  | **Authors** | **Description, scoring and accessibility information** |
| 3 articles   1. Post et al. (2006) [58](-) 2. Storheim et al. (2005) [27](-) 3. Schultz et al. (2005) [34](NS)   Level of evidence (majority negative): MODERATE | Post et al. (2006) [58] | **Tool 46 (T46)**  **Description:** Physical functioning subscale (10 items) of the RAND-36 standardized questionnaire [59]  **Scoring:** 3-point scale: 1. Yes, limited a lot = 0%; 2. Yes, limited a little = 50%; 3. No, not limited at all=100% (score range: 0-100%)  **Interpretation:** Lower scores on this questionnaire means higher perceived disability, which negatively impacts RTW (increase sickness absence duration) of workers with a MSD.  **Accessibility:** Available in [59] and online (automatized report and scoring of the SF-36 version 1): [https://orthotoolkit.com/sf-36/](https://can01.safelinks.protection.outlook.com/?url=https%3A%2F%2Forthotoolkit.com%2Fsf-36%2F&data=05%7C01%7Cdiana.zidarov%40umontreal.ca%7C59eaf5a3e5f442bf548408dab299d9ee%7Cd27eefec2a474be7981e0f8977fa31d8%7C1%7C0%7C638018671109622416%7CUnknown%7CTWFpbGZsb3d8eyJWIjoiMC4wLjAwMDAiLCJQIjoiV2luMzIiLCJBTiI6Ik1haWwiLCJXVCI6Mn0%3D%7C3000%7C%7C%7C&sdata=PVEbQrFv7w5Am5HWpN8jmQxEoiqBVtMefOpLtgegHA0%3D&reserved=0)  Note: The physical functioning subscale is the same for RAND-36 and SF-36 (version 1) [60] |
|  | Storheim et al. (2005) [27] | **Tool 47 (T47)**  **Description:** Physical functioning subscale (10 items) of the SF-36 (version 1) standardized questionnaire [59, 61]  **Scoring:** 3-point scale: 1. Yes, limited a lot = 0%; 2. Yes, limited a little = 50%; 3. No, not limited at all=100% (score range: 0-100%)  **Interpretation:** Lower scores on this questionnaire means higher perceived disability, which negatively impacts RTW (increase sickness absence duration) of workers with a MSD.  **Accessibility:** Available in [59] and online (automatized report and scoring of the SF-36 version 1): [https://orthotoolkit.com/sf-36/](https://can01.safelinks.protection.outlook.com/?url=https%3A%2F%2Forthotoolkit.com%2Fsf-36%2F&data=05%7C01%7Cdiana.zidarov%40umontreal.ca%7C59eaf5a3e5f442bf548408dab299d9ee%7Cd27eefec2a474be7981e0f8977fa31d8%7C1%7C0%7C638018671109622416%7CUnknown%7CTWFpbGZsb3d8eyJWIjoiMC4wLjAwMDAiLCJQIjoiV2luMzIiLCJBTiI6Ik1haWwiLCJXVCI6Mn0%3D%7C3000%7C%7C%7C&sdata=PVEbQrFv7w5Am5HWpN8jmQxEoiqBVtMefOpLtgegHA0%3D&reserved=0) |
|  | **Summary** | 2 studies used the same subscale of a standardized questionnaire (RAND-36 and SF-36 are the same) |

^a^ (+): statistically significant positive association; (-) statistically significant negative association; (NS) non statistically significant association. Rules to determine the level of evidence are explained in Fig. 2. Tools in S3 and S5 files have the same numbers (e.g., T4 correspond to the same tool in both files).

**Table S16.** Evidentiary support for *Catastrophizing (pain)* – Moderate evidence for predicting RTW after a MSD

| **Number of studies and significant results** ^a^ | **Measurement tool used in each study showing a statistically significant predictive value**  **(Tools appraisal using psychometric (n = 6) and usability (n = 4) criteria is provided in Table 4)** | |
| --- | --- | --- |
|  | **Authors** | **Description, scoring and accessibility information** |
| 4 articles   1. Gauthier et al (2006) [62](-) 2. Bosman et al (2019) [39] (-) 3. Turner et al (2008) [4](-) 4. Turner at al (2006) [18] (NS)   Level of evidence (majority negative): MODERATE | Gauthier et al (2006) [62]  Bosman et al (2019) [39] | **Tool 48 (T48)**  **Description:** The Pain Catastrophizing Scale (13 items) [63], describing different thoughts and feeling related to pain, assesses the following dimensions: rumination (4 items), magnification (3 items), and helplessness (6 items).  **Scoring:** 5-point Likert scale, from “not at all” = 0 to “all the time” = 4. Total score range: 0-52  **Interpretation:** Higher scores on this questionnaire means more pain catastrophizing thoughts, which negatively impacts RTW (increase sickness absence duration) of workers with a MSD.  **Accessibility:** Available in [63] and <https://eprovide.mapi-trust.org/instruments/pain-catastrophizing-scale> |
|  | Turner et al (2008) [4] | **Tool 49 (T49)**  **Description:** Mean of responses to 3 items from the Pain Catastrophizing Scale [63], as further detailed in [18]:   1. item 5: *'I feel I can't stand it anymore'* 2. item 4: *'It is awful and I feel that it overwhelms me'* 3. item 11: *'I keep thinking about how badly I want it to stop'*   **Scoring:** 5-point Likert scale, from “not at all” = 0 to “all the time” = 4. Total score range: 0-4.  **Interpretation:** Higher scores on this questionnaire means more pain catastrophizing thoughts, which negatively impacts RTW (increase sickness absence duration) of workers with a MSD.  **Accessibility:** Available in [63] and <https://eprovide.mapi-trust.org/instruments/pain-catastrophizing-scale> |
|  | **Summary** | 2 studies used a standardized questionnaire  1 study used a brief non-validated version of a standardized questionnaire |

^a^ (+): statistically significant positive association; (-) statistically significant negative association; (NS) non statistically significant association. Rules to determine the level of evidence are explained in Fig. 2. Tools in S3 and S5 files have the same numbers (e.g., T4 correspond to the same tool in both files).

**Table S17.** Evidentiary support for *All fear factors* – Moderate evidence for predicting RTW after a MSD

| **Number of studies and significant results** ^a^ | **Measurement tool used in each study showing a statistically significant predictive value**  **(Tools appraisal using psychometric (n = 6) and usability (n = 4) criteria is provided in Table 4)** | |
| --- | --- | --- |
|  | **Authors** | **Description, scoring and accessibility information** |
| 15 articles   1. Corbière, et al (2017)[52] (-) 2. Du Bois, et al (2009) [10] (-) 3. Grøvle et al. (2013) [23] (-) 4. Hagen et al. (2005) [32](-) 5. Opsahl et al. (2016) [24](-) 6. Oyeflaten, et al. (2008) [25] (-) 7. Soucy et al. (2006) [26] (-) 8. Storheim et al. (2005)[27] (-) 9. Turner at al (2006) [18](-) 10. Turner et al. (2008) [4](-) 11. Cougot et al. (2015) [64](NS) 12. Gauthier et al. (2006) [62](NS) * 13. Grøvle et al. (2013) [23](NS) 14. Oyeflaten, et al. (2008) [25] (NS) * 15. Nicholas et al. (2019) [14] (NS)   Level of evidence (majority negative): MODERATE  ***** This study used 2 tools measuring this concept (fear), which would bias the level of evidence if not accounted for. Keeping only the tool with the best psychometric properties , namely the FABQ-W (against FABQ-PA or TSK), this factor remained MODERATE. | Corbière, et al (2017) [52] | **Tool 50 (T50)**  **Description:** Fear of relapse subscale (4 items) of Part A (obstacles of RTW) of the Return-to-Work Obstacles and Self-Efficacy Scale (ROSES) standardized questionnaire [52]  **Scoring:** 7-point Likert scale, from “not an obstacle” = 1 to “big obstacle” = 7; Subscale score range (mean): 1-7.  **Interpretation:** Higher scores on this subscale means that fear of relapse represents an obstacle to the RTW, which negatively impacts RTW (increase sickness absence duration) of workers with a MSD.  **Accessibility:** Available online for free: <http://www.santementaletravail.ca/membership-join/> |
|  | Du Bois, et al (2009) [10] | **Tool 51 (T51)**  **Description:** Single item from the Tampa Scale for Kinesiophobia (TSK) [65] standardized questionnaire: *It is not advisable to be physically active*  **Scoring:** 4-point Likert scale, from “strongly disagree” = 1; “disagree” = 2 and “agree” = 3 to “strongly agree” = 4; Score range: 1-4.  **Interpretation:** Higher scores on this questionnaire means more fear of movement, which negatively impacts RTW (increase sickness absence duration) of workers with a MSD.  **Accessibility:** Available in [65] |
|  | Hagen et al. (2005) [32] | **Tool 52 (T52)**  **Description:** Single item of the Graded Reduced Work Ability Scale[37]: *If you continue working, what effect will that have on your complaints?*  **Scoring:** The 5-point scale was found elsewhere [49]: “a great deal” = 1; “a lot” = 2; “some” = 3; “not much” = 4; “very little” = 5; Score range: 1-5, lower scores indicate higher fears  **Interpretation:** Lower scores on this questionnaire means that higher fears that continuing to work would cause more complaints,, which negatively impacts RTW (increase sickness absence duration) of workers with a MSD.  **Accessibility:** Available in [37] but the information above is more complete (scoring). |
|  | Grøvle et al. (2013) [23]  Opsahl et al. (2016) [24]  Oyeflaten, et al. (2008) [25]  Soucy et al. (2006) [26]  Storheim et al. (2005) [27]  Turner at al (2006) [18]  Turner et al. (2008) [4] | **Tool 53 (T53)**  **Description:** Work subscale (7 items) of the Fear-Avoidance Beliefs Questionnaire (FABQ) [21]  **Scoring:** 7-point Likert scale, from “completely disagree” = 0 to “completely agree” = 6; Subscale score range: 0-42.  **Interpretation:** Higher scores on this subscale means more fear-avoidance of work activities, which negatively impacts RTW (increase sickness absence duration) of workers with a MSD.  **Accessibility:** Available in [21] |
|  | **Summary** | 2 studies used one item of a standardized questionnaire  8 studies used a subscale of standardized questionnaires |

^a^ (+): statistically significant positive association; (-) statistically significant negative association; (NS) non statistically significant association. Rules to determine the level of evidence are explained in Fig. 2. Tools in S3 and S5 files have the same numbers (e.g., T4 correspond to the same tool in both files).

**Table S18.** Evidentiary support for *Illness behaviour* – Moderate evidence for predicting RTW after a MSD

| **Number of studies and significant results** ^a^ | **Measurement tool used in each study showing a statistically significant predictive value**  **(Tools appraisal using psychometric (n = 6) and usability (n = 4) criteria is provided in Table 4)** | |
| --- | --- | --- |
|  | **Authors** | **Description, scoring and accessibility information** |
| 4 articles   1. Gaines & Hegmann (1999) [45] (-) 2. Schultz et al. (2002) [54](-) 3. Schultz et al. (2005) [34](-) 4. Beemster et al. (2021) (NS)   Level of evidence (majority negative): MODERATE | Gaines & Hegmann (1999) [45] | **Tool 54 (T54)**  **Description:** Waddell’s nonorganic signs (n = 8) can be observed during the physical examination, organized in five types: (1) tenderness (2 signs), (2) simulation (2 signs), (3) distraction, (4) regional (2 signs) and (5) overreaction. Only the simulation signs (pain with simulated axial loading; pain with simulated rotation) were predictive of RTW.  **Scoring:** If one of both simulation signs is positive, the simulation type is positive.  **Interpretation:** A positive simulation sign (pain with simulated axial loading or pain with simulated rotation) means more illness behaviours, which negatively impacts RTW (increase sickness absence duration) of workers with a MSD.  **Accessibility:** Described in and more in details in [66] |
|  | Schultz et al. (2002) [54] | **Tool 55 (T55)**  **Description:** The Pain behaviour observation system (PBOS) tests 5 categories of pain behaviour (guarding, touching, words, sounds, and facial expressions), as assessed during a standardized physical examination [67, 68]. Only the 'guarding' pain behaviour was predictive of RTW.  **Scoring:** Coding across multiple physical tests  **Interpretation:** Evidence of guarding pain behaviours would negatively impact RTW (increase sickness absence duration) of workers with a MSD.  **Accessibility:** Not available anymore, as confirmed by Dr Prkachin |
|  | Schultz et al. (2005) [34] | **Tool 56 (T56)**  **Description:** Waddell’s symptoms (n = 7) can be identified during the clinical interview: (1) tailbone pain, (2) whole leg pain, (3) whole leg numbness, (4) whole leg giving way, (5) no pain-free spells, (6) intolerance of treatments, (7) emergency admissions  **Scoring:** Sum of positive symptoms (score range: 0-7)  **Interpretation:** More positive symptoms mean more illness behaviours, which negatively impacts RTW (increase sickness absence duration) of workers with a MSD.  **Accessibility:** Available in [69] |
|  | **Summary** | 2 studies used a standardized testing protocol during physical examination  1 study used the anamnesis of symptoms (clinical interview) |

^a^ (+): statistically significant positive association; (-) statistically significant negative association; (NS) non statistically significant association. Rules to determine the level of evidence are explained in Fig. 2. Tools in S3 and S5 files have the same numbers (e.g., T4 correspond to the same tool in both files).

**Table S19.** Evidentiary support for *Mental vitality* – Moderate evidence for predicting RTW after a MSD

| **Number of studies and significant results** ^a^ | **Measurement tool used in each study showing a statistically significant predictive value**  **(Tools appraisal using psychometric (n = 6) and usability (n = 4) criteria is provided in Table 4)** | |
| --- | --- | --- |
|  | **Authors** | **Description, scoring and accessibility information** |
| 4 articles   1. Schultz et al. (2002) [54](+) 2. Schultz et al. (2005)[34] (+) 3. Selander et al. (2007)[38] (+) 4. Post et al. (2006)[58] (NS)   Level of evidence (majority positive): MODERATE | Schultz et al. (2002) [54]  Schultz et al. (2005) [34]  Selander et al. (2007) [38] | **Tool 57 (T57)**  **Description:** Vitality subscale (4 items) of the SF-36 (version 1) standardized questionnaire [59]  **Scoring:** Two 6-point scales are used, depending on the items: 1. Scoring needs to consider reversed items (score range: 0-100%) [60]  **Interpretation:** Higher scores on this subscale means more vitality, which positively impacts RTW (reduce sickness absence duration) of workers with a MSD.  **Accessibility:** Available in [59] and online (automatized report and scoring of the SF-36 version 1): [https://orthotoolkit.com/sf-36/](https://can01.safelinks.protection.outlook.com/?url=https%3A%2F%2Forthotoolkit.com%2Fsf-36%2F&data=05%7C01%7Cdiana.zidarov%40umontreal.ca%7C59eaf5a3e5f442bf548408dab299d9ee%7Cd27eefec2a474be7981e0f8977fa31d8%7C1%7C0%7C638018671109622416%7CUnknown%7CTWFpbGZsb3d8eyJWIjoiMC4wLjAwMDAiLCJQIjoiV2luMzIiLCJBTiI6Ik1haWwiLCJXVCI6Mn0%3D%7C3000%7C%7C%7C&sdata=PVEbQrFv7w5Am5HWpN8jmQxEoiqBVtMefOpLtgegHA0%3D&reserved=0) |
|  | **Summary** | 3 studies used a subscale from the same standardized questionnaire |

^a^ (+): statistically significant positive association; (-) statistically significant negative association; (NS) non statistically significant association. Rules to determine the level of evidence are explained in Fig. 2. Tools in S3 and S5 files have the same numbers (e.g., T4 correspond to the same tool in both files).

**Table S20.** Evidentiary support for *Positive health change* – Moderate evidence for predicting RTW after a MSD

| **Number of studies and significant results** ^a^ | **Measurement tool used in each study showing a statistically significant predictive value**  **(Tools appraisal using psychometric (n = 6) and usability (n = 4) criteria is provided in Table 4)** | |
| --- | --- | --- |
|  | **Authors** | **Description, scoring and accessibility information** |
| 4 articles   1. Post et al. (2006)[58] (-) 2. Schultz et al. (2002)[54] (-) 3. Schultz et al. (2004) [33](-) 4. Schultz et al. (2005) [34](NS)   Level of evidence (majority negative): MODERATE | Post et al. (2006) [58]  Schultz et al. (2002) [54]  Schultz et al. (2004) [33] | **Tool 58 (T58)**  **Description:** Single item from the RAND-36 or SF-36 version 1 (same as RAND-36)**:** Compared to one year ago, how would you rate your health in general now?  **Scoring:** 5-point Likert scale, from “much better now than one year ago” = 1, “somewhat better now than one year ago” = 2, “about the same as one year ago” = 3 and “somewhat worse now than one year ago” = 4 to “much worse now than one year ago” = 5 (score range = 1-5).  **Interpretation:** A lower score on this subscale means that health has improved in the past year, which positively impacts RTW (increase sickness absence duration) of workers with a MSD.  **Accessibility:** Available in [59] but it is the same as provided above. |
|  | **Summary** | 3 studies used the same item from a standardized questionnaire (RAND-36 and SF-36 (version 1) are the same) |

^a^ (+): statistically significant positive association; (-) statistically significant negative association; (NS) non statistically significant association. Rules to determine the level of evidence are explained in Fig. 2. Tools in S3 and S5 files have the same numbers (e.g., T4 correspond to the same tool in both files).

**Table S21.** Evidentiary support for *Sleep quality* – Moderate evidence for predicting RTW after a MSD

| **Number of studies and significant results** ^a^ | **Measurement tool used in each study showing a statistically significant predictive value**  **(Tools appraisal using psychometric (n = 6) and usability (n = 4) criteria is provided in Table 4)** | |
| --- | --- | --- |
|  | **Authors** | **Description, scoring and accessibility information** |
| 3 articles   1. Hara et al (2018) [13] (+) 2. Nicholas et al (2019) [14] (+) 3. Reme et al (2009) [15] (NS)   Level of evidence (majority negative): MODERATE | Hara et al (2018) [13] | **Tool 59 (T59)**  **Description:** Insomnia Severity Index (7 items)  **Scoring:** Different 0-4 scales, depending on the item (total score range: 0-28)  **Interpretation:** A lower score on this questionnaire means less sleep disturbances, which positively impacts RTW (reduce sickness absence duration) of workers with a MSD.  **Accessibility:**  This questionnaire is copyright protected but available for free here if not for commercial use: <https://eprovide.mapi-trust.org/isi-insomnia-severity-index/> |
|  | Nicholas et al (2019) [14] | **Tool 60 (T60)**  **Description:** Item 4 (sleep disturbance) of the Örebro Musculoskeletal Pain Screening Questionnaire- 10-item-short-form (ÖMPSQ-SF) standardized questionnaire[22]: I can sleep at night.  **Scoring:** 11-point Likert scale, from “can’t do it because of pain problem” = 0 to “can do it without pain being a problem” = 10 (reversed scoring; 10 - x); Score range: 0-10.  **Interpretation:** Considering the reversed score (0 = Can do it without pain being a problem interference; 10 = Can’t do it because of pain problem), a lower score means less sleep disturbances, which positively impacts RTW (reduce sickness absence duration) of workers with a MSD.  **Accessibility:** Available in [70] but the item and response scale are identical as provided above. |
|  | **Summary** | 1 study used a standardized questionnaire  1 study used a single item from a standardized questionnaire |

^a^ (+): statistically significant positive association; (-) statistically significant negative association; (NS) non statistically significant association. Rules to determine the level of evidence are explained in Fig. 2. Tools in S3 and S5 files have the same numbers (e.g., T4 correspond to the same tool in both files).

# Prognostic factors of return to work (CMD)

**Table S22.** Evidentiary support for *Expectation (RTW) –* Strong evidence for predicting RTW after a CMD

| **Number of studies and significant results^a^** | **Measurement tool used in each study showing a statistically significant predictive value**  **(Tools appraisal using psychometric (n = 6) and usability (n = 4) criteria is provided in Table 4)** | |
| --- | --- | --- |
|  | **Authors** | **Description, scoring and accessibility information** |
| 6 articles   1. Hara et al., (2018) [13](+) 2. Løvvik et al., (2014) [71] (+) 3. Nielsen et al., (2011) [72](+) 4. Nieuwenhuijsen et al., (2006)[73] (+) 5. Sampere et al., (2012) [16](+) 6. Hedlund et al. (2022) [74] (+)   Level of evidence (all positive): STRONG | Hara et al., (2018) [13] | **Tool 10 (T10)**  **Description:** One item from Fear Avoidance Beliefs Questionnaire (FABQ) – Work Subscale: *I do not think that I will be back in my ordinary work within three months*  **Scoring:** 7-point scale, from 0 (completely disagree) to 6 (completely agree). Higher score indicates a worse fear of not returning to work. The cut-off was set at ≥3 indicating a non-positive expectation defined as an “uncertain or even poorer expectation of RTW”.  **Interpretation:** A positive expectation concerning RTW is positively associated with RTW (reduce sickness absence duration) of workers with a CMD.  **Accessibility:** Available in [21] |
|  | Løvvik et al., (2014) [71] | **Tool 61 (T61)**  **Description:** This is a single item in the wording: *I expect to be back at work within the next few weeks*.  **Scoring:** 5-point Likert scale (strongly agree to strongly disagree). Responses were grouped into three types comprising those who strongly agreed or agreed into positive RTW-expectations, those answering ‘neither agree nor disagree’ into uncertain RTW-expectations and those either disagreeing or strongly disagreeing into negative RTW-expectations  **Interpretation**: A positive expectation concerning RTW is positively associated with RTW (reduce sickness absence duration) of workers with a CMD.  **Accessibility:** Available in [71] |
|  | Nielsen et al., (2011) [72] | **Tool 62 (T62)**  **Description:** This is a single item in the wording: *Do you expect to be able to return to your workplace?*  **Scoring:** Yes/No  **Interpretation:** : A positive expectation concerning RTW is positively associated with RTW (reduce sickness absence duration) of workers with a CMD.  **Accessibility:** Available in [72] |
|  | Nieuwenhuijsen et al., (2006) [73] | **Tool 63 (T63)**  **Description:** This is a single item in the wording: *How many months do you think it will take you to fully return to work?*  **Scoring:** answers were dichotomized into expected duration ≤3 months versus >3 months  **Interpretation:** A lower time expectation to RTW is positively associated with RTW (reduce sickness absence duration) of workers with a CMD.  **Accessibility:** Available in [73] |
|  | Sampere et al., (2012) [16] | **Tool 13 (T13)**  **Description:** This is a single item in the wording: *Approximately how long do you think you will need to return to the job you had before you went on sick leave? (we understand that this question is difficult to answer, please try to give an answer, even if it is only approximate)*  **Scoring:** Possible answers are ‘Less than 1 week’, ‘Between 1 and 4 weeks’, ‘Between 1 and 3 months’, ‘Between 4 and 6 months’, ‘Over 6 months’, ‘I will never be able to perform the job I used to before’, ‘I do not know’, ‘I have no idea how long I will take to recover’. Response options are then collapsed into <1, 1–3, >3 months, I will never be and I don’t know.  **Interpretation:** Time (lower) estimated by the worker to be able to perform the same job is related to a quicker time to RTW.  **Accessibility:** Available in [16] |
|  | Hedlund et al. (2022) [74] | **Tool 64 (T64)**  **Description:** This is the RTW intention subscale of the RTW Beliefs Questionnaire. It consists of three items: *I expect to return to work within 3 months; I want to return to work within 3 months; I intend to return to work within 3 months.*  **Scoring:** 7-point Likert scale (1= strongly disagree; 7 strongly agree)  **Interpretation:** Stronger intentions to RTW are related to a quicker time to RTW.  **Accessibility:** Available in [75] |
|  | **Summary** | 1 study used a subscale of a standardized questionnaire  5 studies used a single item |

^a^ (+): statistically significant positive association; (-) statistically significant negative association; (NS) non statistically significant association. Rules to determine the level of evidence are explained in Fig. 2. Tools in S3 and S5 files have the same numbers (e.g., T4 correspond to the same tool in both files).

**Table S23**. Evidentiary support for *Job strain* – Moderate evidence for predicting RTW after a CMD

| **Number of studies and significant results^a^** | **Measurement tool used in each study showing a statistically significant predictive value**  **(Tools appraisal using psychometric (n = 6) and usability (n = 4) criteria is provided in Table 4)** | |
| --- | --- | --- |
|  | **Authors** | **Description, scoring and accessibility information** |
| 2 articles   1. Haveraaen et al., 2016 [47] (-) 2. Haveraaen et al., 2017 [48](-)   Level of evidence (all negative): MODERATE | Haveraaen et al., 2016[47]  Haveraaen et al., 2017[48] | **Tool 36 (T36)**  **Description:** Two subscales of the Job Content Questionnaire (5 items for Job demands and 9 items for decision latitude)  **Scoring:** 4-point Likert scale, ‘strongly disagree’ to ‘strongly agree’.  **Interpretation:** High levels of job strain negatively impacts RTW (increase sickness absence duration) of workers with a MSD.  **Accessibility:** This questionnaire is copyright protected. Requests should be made to the JCQ Center in Denmark by telephone (+45 40461000) or e-mail (jcqcenter@oresundsynergy.com). Access is free in the vast majority of cases, but a fee may be charged for research and commercial projects. |
|  | **Summary** | 2 studies used a standardized questionnaire |

^a^ (+): statistically significant positive association; (-) statistically significant negative association; (NS) non statistically significant association. Rules to determine the level of evidence are explained in Fig. 2. Tools in S3 and S5 files have the same numbers (e.g., T4 correspond to the same tool in both files).

**Table S24.** Evidentiary support for *Job demands (psychological)* – Moderate evidence for predicting RTW after a CMD

| **Number of studies and significant results *** | **Measurement tool used in each study showing a statistically significant predictive value**  **(Tools appraisal using psychometric (n = 6) and usability (n = 4) criteria is provided in Table 4)** | |
| --- | --- | --- |
|  | **Authors** | **Description, scoring and accessibility information** |
| 2 articles   1. Haveraaen et al., 2016 [47] (-) 2. Haveraaen et al., 2017 [48] (-)   Level of evidence (all negative): MODERATE | Haveraaen et al., 2016 [47]  Haveraaen et al., 2017 [48] | **Tool 65 (T65)**  **Description:** Subscale of the Job Content Questionnaire (psychological demands, 5 items)  **Scoring:** 4-point Likert scale, ‘strongly disagree’ to ‘strongly agree’  **Interpretation:** High levels of job demands (psychological) negatively impacts RTW (increase sickness absence duration) of workers with a MSD.  **Accessibility:** This questionnaire is copyright protected. Requests should be made to the JCQ Center in Denmark by telephone (+45 40461000) or e-mail (jcqcenter@oresundsynergy.com). Access is free in the vast majority of cases, but a fee may be charged for research and commercial projects. |
|  | **Summary** | Two papers used a standardized questionnaire |

^a^ (+): statistically significant positive association; (-) statistically significant negative association; (NS) non statistically significant association. Rules to determine the level of evidence are explained in Fig. 2. Tools in S3 and S5 files have the same numbers (e.g., T4 correspond to the same tool in both files).

**Table S25.** Evidentiary support for *Sleep quality* – Moderate evidence for predicting RTW after a CMD

| **Number of studies and significant results** ^a^ | **Measurement tool used in each study showing a statistically significant predictive value**  **(Tools appraisal using psychometric (n = 6) and usability (n = 4) criteria is provided in Table 4)** | |
| --- | --- | --- |
|  | **Authors** | **Description, scoring and accessibility information** |
| 2 articles   1. Gustafsson et al. (2013) [76](+) 2. Hara et al. (2018) [13] (+)   Level of evidence (all positive): MODERATE | Gustafsson et al. (2013) [76] | **Tool 66 (T66)**  **Description:** Single item from the Comprehensive Psychopathological Rating Scale Self Administered (CPRS-S-A): *Subjective experience of reduced duration or depth of sleep compared with the subject’s normal pattern when well.*  Note: Gustafsson et al. [76] re-phrased this item in a self-rating format as per Svanborg and Asberg [77] . However, the re-phrased item was not available in the article and our attempts to obtain it were unsuccessful.  **Scoring:** 4-point Likert scale, from “sleeps as usual” = 0; “slight difficulty dropping off to sleep or slightly reduced, light or fitful sleep” = 1 and “sleep reduced or broken by at least 2 hours” = 2 to “less than two- or three-hours’ sleep” = 3), dichotomized to 0 (‘Sleeps as usual’ and ‘Slight difficulty…’) and 1 (‘Sleep reduced…’ and ‘Less than…’), thus 1 indicating sleep disturbance.  **Interpretation:** A score lower than 2 on this 4-point scale means less sleep disturbances, which positively impacts RTW (reduce sickness absence duration) of workers with a CMD.  **Accessibility:** Available in [78], item 19, but please consider the note above (Description). |
|  | Hara et al. (2018) [13] | **Tool 59 (T59)**  **Description:** Insomnia Severity Index (7 items)  **Scoring:** Different 0-4 scales, depending on the item (total score range: 0-28)  **Interpretation:** A lower score on this questionnaire means less sleep disturbances, which positively impacts RTW (reduce sickness absence duration) of workers with a CMD.  **Accessibility:** Available in [79] |
|  | **Summary** | 1 study used a single item from a modified standardized questionnaire  1 study used a standardized questionnaire |

^a^ (+): statistically significant positive association; (-) statistically significant negative association; (NS) non statistically significant association. Rules to determine the level of evidence are explained in Fig. 2. Tools in S3 and S5 files have the same numbers (e.g., T4 correspond to the same tool in both files).

**Table S26.** Evidentiary support for *All participation factors* – Moderate evidence for predicting RTW after a CMD

| **Number of studies and significant results** ^a^ | **Measurement tool used in each study showing a statistically significant predictive value**  **(Tools appraisal using psychometric (n = 6) and usability (n = 4) criteria is provided in Table 4)** | |
| --- | --- | --- |
|  | **Authors** | **Description, scoring and accessibility information** |
| 3 articles   1. Gustaffson et al. (2013)[76] (+) 2. Laukkala et al. (2018) [80](+) 3. Post et al. (2006)[58] (NS)   Level of evidence (majority positive): MODERATE | Gustaffson et al. (2013) [76] | **Tool 67 (T67)**  **Description:** Social functioning subscale (2 items) of the Swedish SF-36 (version 2) standardized questionnaire, measuring participation to social life  **Scoring:** Different 5-point scales, depending on the item (subscale score range: 0-100)  **Interpretation:** A higher score on this subscale means higher social functioning, which positively impacts RTW (reduce sickness absence duration) of workers with a CMD.  **Accessibility:** Available online (fees): <https://www.qualitymetric.com/health-surveys-old/the-sf-36v2-health-survey/> |
|  | Laukkala et al. (2018) [80] | **Tool 68 (T68)**  **Description:** The Social and Occupational Functioning Assessment Scale (SOFAS) is a single-item rating from the DSM-IV, measuring participation in general (social and occupational activities)  **Scoring:** Global descriptions of social and occupational functioning are provided and ranked into 10 equal intervals (score range: 0 to 100, with lower scores representing lower functioning)  **Interpretation:** A higher score on this subscale means higher social functioning, which positively impacts RTW (reduce sickness absence duration) of workers with a CMD.  **Accessibility:** Available in [81] DSM-IV as an Axis V measure |
|  | **Summary** | 1 study used a subscale from a standardized questionnaire  1 study used a single-item tool |

^a^ (+): statistically significant positive association; (-) statistically significant negative association; (NS) non statistically significant association. Rules to determine the level of evidence are explained in Fig. 2. Tools in S3 and S5 files have the same numbers (e.g., T4 correspond to the same tool in both files).

# **References**

1. Hogg-Johnson S, Cole D. Early prognostic factors for duration on temporary total benefits in the first year among workers with compensated occupational soft tissue injuries. Occupational and Environmental Medicine. 2003;60(4):244-53.

2. Franche R-L, Severin CN, Hogg-Johnson S, Côté P, Vidmar M, Lee H. The impact of early workplace-based return-to-work strategies on work absence duration: a 6-month longitudinal study following an occupational musculoskeletal injury. Journal of occupational and environmental medicine. 2007;49(9):960-74. doi: 10.1097/jom.0b013e31814b2e9f. PubMed PMID: 17848852.

3. Iles RA, Sheehan LR, Gosling CM. Assessment of a new tool to improve case manager identification of delayed return to work in the first two weeks of a workers' compensation claim. Clin Rehabil. 2020;34(5):656-66. doi: 10.1177/0269215520911417. PubMed PMID: 32183561.

4. Turner JA, Franklin G, Fulton-Kehoe D, Sheppard L, Stover B, Wu R, et al. ISSLS prize winner: early predictors of chronic work disability: a prospective, population-based study of workers with back injuries. Spine (Phila Pa 1976). 2008;33(25):2809-18. Epub 2008/12/04. doi: 10.1097/BRS.0b013e31817df7a7. PubMed PMID: 19050587.

5. Gross DP, Battié MC. Factors influencing results of functional capacity evaluations in workers' compensation claimants with low back pain. Physical therapy. 2005;85(4):315-22.

6. Iles R, Sheehan L, Munk K, Gosling C. Development and pilot assessment of the PACE tool: helping case managers identify and respond to risk factors in workers’ compensation case management. Journal of Occupational Rehabilitation. 2020;30(2):167-82.

7. Steenstra IA, Busse JW, Tolusso D, Davilmar A, Lee H, Furlan AD, et al. Predicting time on prolonged benefits for injured workers with acute back pain. J Occup Rehabil. 2015;25(2):267-78. Epub 2014/08/29. doi: 10.1007/s10926-014-9534-5. PubMed PMID: 25164779; PubMed Central PMCID: PMC4436678.

8. Reneman MF, Beemster TT, Welling SJ, Mierau JO, Dijk HH. Vocational Rehabilitation for Patients with Chronic Musculoskeletal Pain With or Without a Work Module: An Economic Evaluation. J Occup Rehabil. 2021;31(1):84-91. Epub 2020/08/21. doi: 10.1007/s10926-020-09921-y. PubMed PMID: 32816203; PubMed Central PMCID: PMC7954755.

9. Du Bois M, Donceel P. A screening questionnaire to predict no return to work within 3 months for low back pain claimants. European Spine Journal. 2008;17(3):380-5. doi: 10.1007/s00586-007-0567-8.

10. Du Bois M, Szpalski M, Donceel P. Patients at risk for long-term sick leave because of low back pain. Spine J. 2009;9(5):350-9. doi: 10.1016/j.spinee.2008.07.003. PubMed PMID: 18790677.

11. Fishbain DA, Cutler RB, Rosomoff HL, Khalil T, Steele-Rosomoff R. Impact of chronic pain patients' job perception variables on actual return to work. Clin J Pain. 1997;13(3):197-206. doi: 10.1097/00002508-199709000-00004. PubMed PMID: 9303251.

12. Gross DP, Battie MC. Recovery Expectations Predict Recovery in Workers With Back Pain but Not Other Musculoskeletal Conditions. J Spinal DisordTech. 2010.

13. Hara KW, Bjorngaard JH, Jacobsen HB, Borchgrevink PC, Johnsen R, Stiles TC, et al. Biopsychosocial predictors and trajectories of work participation after transdiagnostic occupational rehabilitation of participants with mental and somatic disorders: a cohort study. BMC Public Health. 2018;18(1):1014. doi: 10.1186/s12889-018-5803-0. PubMed PMID: 30111291; PubMed Central PMCID: PMCPMC6094579.

14. Nicholas MK, Costa DSJ, Linton SJ, Main CJ, Shaw WS, Pearce R, et al. Predicting Return to Work in a Heterogeneous Sample of Recently Injured Workers Using the Brief OMPSQ-SF. J Occup Rehabil. 2019;29(2):295-302. Epub 2018/05/26. doi: 10.1007/s10926-018-9784-8. PubMed PMID: 29796980.

15. Reme SE, Hagen EM, Eriksen HR. Expectations, perceptions, and physiotherapy predict prolonged sick leave in subacute low back pain. BMC Musculoskelet Disord. 2009;10:139. doi: 10.1186/1471-2474-10-139. PubMed PMID: 19912626; PubMed Central PMCID: PMCPMC2780378.

16. Sampere M, Gimeno D, Serra C, Plana M, Lopez JC, Martinez JM, et al. Return to work expectations of workers on long-term non-work-related sick leave. Journal of Occupational Rehabilitation. 2012;22(1):15-26. doi: 10.1007/s10926-011-9313-5.

17. Steenstra IA, Koopman FS, Knol DL, Kat E, Bongers PM, de Vet HC, et al. Prognostic factors for duration of sick leave due to low-back pain in dutch health care professionals. J Occup Rehabil. 2005;15(4):591-605. doi: 10.1007/s10926-005-8037-9. PubMed PMID: 16254758.

18. Turner JA, Franklin G, Fulton-Kehoe D, Sheppard L, Wickizer TM, Wu R, et al. Worker recovery expectations and fear-avoidance predict work disability in a population-based workers' compensation back pain sample. Spine. 2006;31(6):682-9.

19. Wahlin C, Ekberg K, Persson J, Bernfort L, Oberg B. Association between clinical and work-related interventions and return-to-work for patients with musculoskeletal or mental disorders. J Rehabil Med. 2012;44(4):355-62.

20. Linton SJ, Boersma K. Early identification of patients at risk of developing a persistent back problem: the predictive validity of the Orebro Musculoskeletal Pain Questionnaire. Clin J Pain. 2003;19(2):80-6. Epub 2003/03/05. doi: 10.1097/00002508-200303000-00002. PubMed PMID: 12616177.

21. Waddell G, Newton M, Henderson I, Somerville D, Main CJ. A Fear-Avoidance Beliefs Questionnaire (FABQ) and the role of fear-avoidance beliefs in chronic low back pain and disability. Pain. 1993;52(2):157-68. Epub 1993/02/01. doi: 10.1016/0304-3959(93)90127-b. PubMed PMID: 8455963.

22. Linton SJ, Nicholas M, MacDonald S. Development of a short form of the Örebro Musculoskeletal Pain Screening Questionnaire. Spine (Phila Pa 1976). 2011;36(22):1891-5. Epub 2010/12/31. doi: 10.1097/BRS.0b013e3181f8f775. PubMed PMID: 21192286.

23. Grøvle L, Haugen AJ, Keller A, Ntvig B, Brox JI, Grotle M. Prognostic factors for return to work in patients with sciatica. The Spine Journal: Official Journal of the North American Spine Society. 2013;13(12):1849-57. doi: 10.1016/j.spinee.2013.07.433.

24. Opsahl J, Eriksen HR, Tveito TH. Do expectancies of return to work and Job satisfaction predict actual return to work in workers with long lasting LBP? BMC Musculoskeletal Disorders. 2016;17. doi: 10.1186/s12891-016-1314-2.

25. Oyeflaten I, Hysing M, Eriksen HR. Prognostic factors associated with return to work following multidisciplinary vocational rehabilitation. J Rehabil Med. 2008;40(7):548-54. doi: 10.2340/16501977-0202. PubMed PMID: 18758672.

26. Soucy I, Truchon M, Côté D. Work-related factors contributing to chronic disability in low back pain. Work (Reading, Mass). 2006;26(3):313-26.

27. Storheim K, Brox JI, Holm I, Bo K. Predictors of return to work in patients sick listed for sub-acute low back pain: a 12-month follow-up study. J Rehabil Med. 2005;37(6):365-71. doi: 10.1080/16501970510040344.

28. Koopman FS, Edelaar M, Slikker R, Reynders K, van der Woude LH, Hoozemans MJ. Effectiveness of a multidisciplinary occupational training program for chronic low back pain: a prospective cohort study. Am J Phys Med Rehabil. 2004;83(2):94-103. doi: 10.1097/01.PHM.0000107482.35803.11. PubMed PMID: 14758295.

29. Truchon M, Côté D. Predictive validity of the Chronic Pain Coping Inventory in subacute low back pain. Pain. 2005;116(3):205-12.

30. Rashid M, Kristofferzon ML, Nilsson A. Predictors of return to work among women with long-term neck/shoulder and/or back pain: A 1-year prospective study. PLoS One. 2021;16(11):e0260490. Epub 2021/11/24. doi: 10.1371/journal.pone.0260490. PubMed PMID: 34813601; PubMed Central PMCID: PMC8610267.

31. Cole DC, Mondloch MV, Hogg-Johnson S. Listening to injured workers: how recovery expectations predict outcomes--a prospective study. CMAJ. 2002;166(6):749-54.

32. Hagen EM, Svensen E, Eriksen HR. Predictors and modifiers of treatment effect influencing sick leave in subacute low back pain patients. Spine (Phila Pa 1976). 2005;30(24):2717-23. PubMed PMID: 16371893.

33. Schultz I, Crook J, Meloche G, Berkowitz J, Milner R, Zuberbier O, et al. Psychosocial factors predictive of occupational low back disability: towards development of a return-to-work model. Pain. 2004;107(1-2):77-85.

34. Schultz IZ, Crook J, Berkowitz J, Milner R, Meloche GR. Predicting Return to Work After Low Back Injury Using the Psychosocial Risk for Occupational Disability Instrument: A Validation Study. Journal of Occupational Rehabilitation. 2005;15(3):365-76. doi: 10.1007/s10926-005-5943-9.

35. Ebrahim S, Malachowski C, Kamal El Din M, Mulla SM, Montoya L, Bance S, et al. Measures of patients' expectations about recovery: a systematic review. J Occup Rehabil. 2015;25(1):240-55. Epub 2014/08/08. doi: 10.1007/s10926-014-9535-4. PubMed PMID: 25100443.

36. Sandström J, Esbjörnsson E. Return to work after rehabilitation. The significance of the patient's own prediction. Scand J Rehabil Med. 1986;18(1):29-33. Epub 1986/01/01. PubMed PMID: 2940677.

37. Haldorsen EM, Indahl A, Ursin H. Patients with low back pain not returning to work. A 12-month follow-up study. Spine. 1998;23(11):1202-7.

38. Selander J, Marnetoft SU, Asell M. Predictors for successful vocational rehabilitation for clients with back pain problems. Disabil Rehabil. 2007;29(3):215-20. doi: 10.1080/09638280600756208. PubMed PMID: 17364772.

39. Bosman LC, Twisk JWR, Geraedts AS, Heymans MW. Development of Prediction Model for the Prognosis of Sick Leave Due to Low Back Pain. J Occup Environ Med. 2019;61(12):1065-71. Epub 2019/10/28. doi: 10.1097/jom.0000000000001749. PubMed PMID: 31651601.

40. Hansson TH, Hansson EK. The effects of common medical interventions on pain, back function, and work resumption in patients with chronic low back pain: A prospective 2-year cohort study in six countries. Spine (Phila Pa 1976). 2000;25(23):3055-64. Epub 2001/01/06. doi: 10.1097/00007632-200012010-00013. PubMed PMID: 11145817.

41. Lötters F, Burdorf A. Prognostic factors for duration of sickness absence due to musculoskeletal disorders. The Clinical Journal of Pain. 2006;22(2):212-21.

42. Amick BC, 3rd, Lee H, Hogg-Johnson S, Katz JN, Brouwer S, Franche RL, et al. How Do Organizational Policies and Practices Affect Return to Work and Work Role Functioning Following a Musculoskeletal Injury? J Occup Rehabil. 2017;27(3):393-404. Epub 2016/09/23. doi: 10.1007/s10926-016-9668-8. PubMed PMID: 27654622.

43. Huijs JJ, Koppes LL, Taris TW, Blonk RW. Differences in predictors of return to work among long-term sick-listed employees with different self-reported reasons for sick leave. J Occup Rehabil. 2012;22(3):301-11. Epub 2012/02/04. doi: 10.1007/s10926-011-9351-z. PubMed PMID: 22302668.

44. Abásolo L, Carmona L, Lajas C, Candelas G, Blanco M, Loza E, et al. Prognostic factors in short-term disability due to musculoskeletal disorders. Arthritis Rheum. 2008;59(4):489-96. Epub 2008/04/03. doi: 10.1002/art.23537. PubMed PMID: 18383421.

45. Gaines WG, Jr., Hegmann KT. Effectiveness of Waddell's nonorganic signs in predicting a delayed return to regular work in patients experiencing acute occupational low back pain. Spine (Phila Pa 1976). 1999;24(4):396-400; discussion 1. Epub 1999/03/05. doi: 10.1097/00007632-199902150-00021. PubMed PMID: 10065525.

46. Hildebrandt VH, Bongers PM, van Dijk FJH, Kemper HCG, Dul J. Dutch Musculoskeletal Questionnaire: description and basic qualities. Ergonomics. 2001;44(12):1038-55. doi: 10.1080/00140130110087437.

47. Haveraaen LA, Skarpaas LS, Berg JE, Aas RW. Do psychological job demands, decision control and social support predictreturn to work three months after a return-to-work (RTW) programme? The rapid-RTW cohort study. Work. 2015;53(1):61-71. Epub 2015/12/20. doi: 10.3233/WOR-152216. PubMed PMID: 26684705.

48. Haveraaen LA, Skarpaas LS, Aas RW. Job demands and decision control predicted return to work: the rapid-RTW cohort study. BMC Public Health. 2017;17(1):154. Epub 2017/02/06. doi: 10.1186/s12889-016-3942-8. PubMed PMID: 28152995; PubMed Central PMCID: PMCPMC5288870.

49. C C. Changing perceptions of work ability in people with low back pain: a feasibility and economic evaluation: University of Nottingham; 2012.

50. Reiso H, Nygård JF, Brage S, Gulbrandsen P, Tellnes G. Work ability and duration of certified sickness absence. Scand J Public Health. 2001;29(3):218-25. Epub 2001/10/30. PubMed PMID: 11680774.

51. Ilmarinen J. The Work Ability Index (WAI). Occupational Medicine. 2007;57(2):160-. doi: 10.1093/occmed/kqm008.

52. Corbiere M, Negrini A, Durand MJ, St-Arnaud L, Briand C, Fassier JB, et al. Development of the Return-to-Work Obstacles and Self-Efficacy Scale (ROSES) and Validation with Workers Suffering from a Common Mental Disorder or Musculoskeletal Disorder. J Occup Rehabil. 2017;27(3):329-41. doi: 10.1007/s10926-016-9661-2. PubMed PMID: 27562583.

53. Lagerveld SE, Blonk RWB, Brenninkmeijer V, Schaufeli WB. Return to work among employees with mental health problems: Development and validation of a self-efficacy questionnaire. Work & Stress. 2010;24(4):359-75. doi: 10.1080/02678373.2010.532644.

54. Schultz IZ, Crook JM, Berkowitz J, Meloche GR, Milner R, Zuberbier OA, et al. Biopsychosocial multivariate predictive model of occupational low back disability. Spine. 2002;27(23):2720-5.

55. Asher AL, Devin CJ, Archer KR, Chotai S, Parker S, Bydon M, et al. An analysis from the Quality Outcomes Database, Part 2. Predictive model for return to work after elective surgery for lumbar degenerative disease. Journal of Neurosurgery Spine. 2017:1-12. doi: 10.3171/2016.8.SPINE16527.

56. Fairbank JC. Why are there different versions of the Oswestry Disability Index? J Neurosurg Spine. 2014;20(1):83-6. Epub 2013/11/12. doi: 10.3171/2013.9.Spine13344. PubMed PMID: 24206036.

57. Fairbank JC, Pynsent PB. The Oswestry Disability Index. Spine (Phila Pa 1976). 2000;25(22):2940-52; discussion 52. Epub 2000/11/14. doi: 10.1097/00007632-200011150-00017. PubMed PMID: 11074683.

58. Post M, Krol B, Groothoff JW. Self-rated health as a predictor of return to work among employees on long-term sickness absence. Disability and Rehabilitation: An International, Multidisciplinary Journal. 2006;28(5):289-97. doi: 10.1080/09638280500160303.

59. Ware JE, Snow KK, Kosinski M, Gandek B, New England Medical Center Hospital Health I. SF-36 health survey : manual and interpretation guide. Boston: Health Institute, New England Medical Center; 1993.

60. Hays RD, Sherbourne CD, Mazel RM. The RAND 36-Item Health Survey 1.0. Health Econ. 1993;2(3):217-27. Epub 1993/10/01. doi: 10.1002/hec.4730020305. PubMed PMID: 8275167.

61. Ware JE, Jr. SF-36 health survey update. Spine (Phila Pa 1976). 2000;25(24):3130-9. Epub 2000/12/22. doi: 10.1097/00007632-200012150-00008. PubMed PMID: 11124729.

62. Gauthier N, Sullivan MJ, Adams H, Stanish WD, Thibault P. Investigating risk factors for chronicity: the importance of distinguishing between return-to-work status and self-report measures of disability. JOccupEnvironMed. 2006;48(3):312-8.

63. Sullivan MJL, Bishop SR, Pivik J. The Pain Catastrophizing Scale: Development and validation. Psychological Assessment. 1995;7(4):524-32. doi: 10.1037/1040-3590.7.4.524.

64. Cougot B, Petit A, Paget C, Roedlich C, Fleury-Bahi G, Fouquet M, et al. Chronic low back pain among French healthcare workers and prognostic factors of return to work (RTW): a non-randomized controlled trial. J Occup Med Toxicol. 2015;10:40. doi: 10.1186/s12995-015-0082-5. PubMed PMID: 26516339; PubMed Central PMCID: PMCPMC4625968.

65. Vlaeyen JWS, Kole-Snijders AMJ, Boeren RGB, van Eek H. Fear of movement/(re)injury in chronic low back pain and its relation to behavioral performance. Pain. 1995;62(3):363-72. Epub 1995/09/01. doi: 10.1016/0304-3959(94)00279-n. PubMed PMID: 8657437.

66. Waddell G, McCulloch JA, Kummel E, Venner RM. Nonorganic physical signs in low-back pain. Spine (Phila Pa 1976). 1980;5(2):117-25. Epub 1980/03/01. doi: 10.1097/00007632-198003000-00005. PubMed PMID: 6446157.

67. Prkachin KM, Hughes E, Schultz I, Joy P, Hunt D. Real-time assessment of pain behavior during clinical assessment of low back pain patients. Pain. 2002;95(1-2):23-30. Epub 2002/01/16. doi: 10.1016/s0304-3959(01)00369-4. PubMed PMID: 11790464.

68. Prkachin KM, Schultz I, Berkowitz J, Hughes E, Hunt D. Assessing pain behaviour of low-back pain patients in real time: concurrent validity and examiner sensitivity. Behav Res Ther. 2002;40(5):595-607. Epub 2002/06/01. doi: 10.1016/s0005-7967(01)00075-4. PubMed PMID: 12038651.

69. Waddell G, Main CJ, Morris EW, Di Paola M, Gray IC. Chronic low-back pain, psychologic distress, and illness behavior. Spine (Phila Pa 1976). 1984;9(2):209-13. Epub 1984/03/01. doi: 10.1097/00007632-198403000-00013. PubMed PMID: 6233714.

70. SJ L. Manual for the Örebro Musculoskeletal Pain Screening Questionnaire: the early identification of patients at risk for chronic pain: Department of Occupational and Environmental Medicine, Örebro Medical Center. 1999.

71. Løvvik C, Shaw W, Øverland S, Reme SE. Expectations and illness perceptions as predictors of benefit recipiency among workers with common mental disorders: secondary analysis from a randomised controlled trial. BMJ Open. 2014;4(3):e004321. doi: 10.1136/bmjopen-2013-004321.

72. Nielsen MB, Madsen IE, Bultmann U, Christensen U, Diderichsen F, Rugulies R. Predictors of return to work in employees sick-listed with mental health problems: findings from a longitudinal study. Eur J Public Health. 2011;21(6):806-11. Epub 2010/12/04. doi: 10.1093/eurpub/ckq171. PubMed PMID: 21126986.

73. Nieuwenhuijsen K, Verbeek JH, de Boer AG, Blonk RW, van Dijk FJ. Predicting the duration of sickness absence for patients with common mental disorders in occupational health care. Scand J Work Environ Health. 2006;32(1):67-74. PubMed PMID: 16539174.

74. Hedlund Å, Nilsson A, Boman E, Kristofferzon M-L. Predictors of return to work and psychological well-being among women during/after long-term sick leave due to common mental disorders - a prospective cohort study based on the theory of planned behaviour. Health & Social Care in the Community. 2022;30(6):e5245-e58. doi: <https://doi.org/10.1111/hsc.13943>.

75. Hedlund Å, Kristofferzon ML, Boman E, Nilsson A. Are return to work beliefs, psychological well-being and perceived health related to return-to-work intentions among women on long-term sick leave for common mental disorders? A cross-sectional study based on the theory of planned behaviour. BMC Public Health. 2021;21(1):535. Epub 2021/03/21. doi: 10.1186/s12889-021-10562-w. PubMed PMID: 33740921; PubMed Central PMCID: PMC7977300.

76. Gustafsson K, Lundh G, Svedberg P, Linder J, Alexanderson K, Marklund S. Psychological factors are related to return to work among long-term sickness absentees who have undergone a multidisciplinary medical assessment. J Rehabil Med. 2013;45(2):186-91. doi: 10.2340/16501977-1077. PubMed PMID: 23138390.

77. Svanborg P, Asberg M. A new self-rating scale for depression and anxiety states based on the Comprehensive Psychopathological Rating Scale. Acta Psychiatr Scand. 1994;89(1):21-8. Epub 1994/01/01. doi: 10.1111/j.1600-0447.1994.tb01480.x. PubMed PMID: 8140903.

78. Asberg M, Montgomery SA, Perris C, Schalling D, Sedvall G. A comprehensive psychopathological rating scale. Acta Psychiatr Scand Suppl. 1978;(271):5-27. Epub 1978/01/01. doi: 10.1111/j.1600-0447.1978.tb02357.x. PubMed PMID: 277059.

79. Bastien CH, Vallières A, Morin CM. Validation of the Insomnia Severity Index as an outcome measure for insomnia research. Sleep Med. 2001;2(4):297-307. Epub 2001/07/05. doi: 10.1016/s1389-9457(00)00065-4. PubMed PMID: 11438246.

80. Laukkala T, Heikinheimo S, Vuokko A, Junttila IS, Tuisku K. Subjective and objective measures of function and return to work: an observational study with a clinical psychiatric cohort. Soc Psychiatry Psychiatr Epidemiol. 2018;53(5):537-40. Epub 2017/12/25. doi: 10.1007/s00127-017-1479-5. PubMed PMID: 29275503.

81. Cooper J. Diagnostic and Statistical Manual of Mental Disorders (4th edn, text revision) (DSM–IV–TR) Washington, DC: American Psychiatric Association 2000. 943 pp. £39.99 (hb). ISBN 0 89042 025 4. British Journal of Psychiatry. 2001;179(1):85-. Epub 2018/01/02. doi: 10.1192/bjp.179.1.85-a.
