# Supplementary material for: Systematic review and tools appraisal of prognostic factors of return to work in workers on sick leave due to musculoskeletal and common mental disorders
Source: PLoS One. 2024 Jul 17;19(7):e0307284. doi: 10.1371/journal.pone.0307284 (PMC11253986; doi:10.1371/journal.pone.0307284)
Supplement: S4 File — (DOCX) [file pone.0307284.s004.docx]

**Supplementary material 4, Table S27**. Summary of evidentiary support for the MSDs and CMDs prognostic factors of return to work reaching inconsistent or insufficient level of evidence or showing only non-significant results.

| **Inconsistent evidence** | **Insufficient evidence** | **Only non-significant results** |
| --- | --- | --- |
| Body functions and structures | | |
| *Body functions (reflexes)*:  **MSD =** 1-/2; Neg.: [1]; Pos.: none; N.S.: [2] | *Body functions (joint mobility/flexibility)*:  **MSD** = 1-, 1+/3; Neg.: [2]; Pos.: [3]; N.S.: [4] | *Body functions (pain):* **MSD =** 1 [5] |
| *Body functions (muscular)*:  **MSD =** 1-/2; Neg.: [4] ; Pos.: none; N.S.: [2] | *CMD symptoms (anxiety)*:  **MSD =** 2-/6; Neg.: [6, 7]; Pos.: none; N.S.: [4, 8-10] | *Body functions (sensory):* **MSD =** 1 [2] |
| *CMD symptoms (depression/Beck-Depressions-Inventory):*  **MSD =** 1-/2; Neg.: [4]; Pos.: none; N.S.: [2] | *All CMD symptoms (depression) tools*  **MSD =** 4-/10; Neg.: [6, 7, 11, 12]; Pos.: none; N.S.: [5, 8, 10, 13-15]  **CMD =** 2-/6; Neg.: [16, 17]; Pos.: none; N.S.: [10, 18-20] | *CMD symptoms (anxiety):* **CMD =** 1 [10] |
| *CMD symptoms (depression/Center for Epidemiologic Studies Depression Scale)*:  **MSD =** 1-/2; Neg.: [11]; Pos.: none; N.S.: [14] | *All CMD symptoms factors*:  **MSD =** 4-/12; Neg.: [6, 7, 11, 12]; Pos.: none; N.S.: [4, 5, 8-10, 13-15, 21]  **CMD =** 2-/7; Neg.: [16, 17]; Pos.: none; N.S.: [10, 18-21] | *CMD symptoms (cognitive difficulties):*  **MSD =** 1 [21];  **CMD =** 1 [21] |
| *Pain qualities:*  **MSD =** 1-/2; Neg.: [13]; Pos.: none; N.S.: [12] | *Pain intensity:*  **MSD =** 6-/19; Neg.: [7, 8, 14, 22-24]; Pos.: none; N.S.: [2, 4, 11, 15, 25-27], [28]-DNK, [28]-GER, [28]-ISR, [28]-NLD, [28]-SWE, [28]-US ^b^ | *CMD symptoms (general):* **CMD =** 1 [29] |
|  |  | *Pain intensity:* **CMD =** 1 [17] |
|  |  | *Pain interference:* **CMD =** 1 [30] |
| *MSD symptoms:*  **MSD =** 1-/2; Neg.: [2]; Pos.: none; N.S.: [22] | *Pain interference:*  ***MSD =*** *3-/7;* Neg.: [26, 31, 32]; Pos.: none; N.S.: [25, 30, 33, 34] | *Physical capacity:* **MSD =** 1 [33] |
| Activities & Participation | | |
| *Activities (disability/Quebec Back Pain Disability Questionnaire):*  **MSD =** 1-/2; Neg.: [35]; Pos.: none; N.S.: [33] | *All activities - disability questionnaire tools*:  **MSD =** 14-/29; Neg.: [1, 4, 7, 8, 24, 26, 27, 30, 34-39]; Pos.: none; N.S.: [2, 11, 14, 15, 22, 33, 40-44], [28]-DNK, [28]-GER, [28]-NLD, [28]-US ^b^  **CMD =** 2-/5: Neg.: [16, 45]; N.S.: [29, 30, 44] | *Activities (disability/SF-36):* **CMD =** 1 [30] |
| *Activities (disability/Roland Morris Disability Questionnaire)*:  **MSD =** 5-/10; Neg.: [1, 4, 27, 36, 37]; Pos.: none; N.S.: [2, 14, 15, 22, 43] | *Quality of life:*  **MSD =** 1+/4; Neg.: none; Pos.: [46]; N.S.: [8, 27, 44] | *Activities (handling stress / psychological demands)*: **MSD =** 1 [33] |
| *Activities (mobility)*:  **MSD =** 1-/2; Neg.: [47]; Pos.: none; N.S.: [8] |  | *Role limitations (emotional):*  **MSD =** 2 [30, 48]; **CMD =**3 [16, 30, 48] |
| *All activities - physical factors*:  **MSD =** 16-/32; Neg.: [1, 4, 7, 8, 24, 26, 27, 30, 34-39, 47, 49]; Pos.: none; N.S.: [2, 8, 11, 14, 15, 22, 33, 40-44], [28]-DNK, [28]-GER, [28]-NLD, [28]-US ^b^  **CMD =** 2-/5: Neg.: [16, 45]; N.S.: [29, 30, 44] |  | *Role limitations (physical):* **CMD =** 2 [30, 48] |
|  |  | *Participation (relationships):* **MSD =** 1 [33] |
|  |  | *Participation (social life):* **MSD =** 1 [30] |
| *Participation (social life):*  **CMD =** 1+/2; Neg.: none; Pos.: [16]; N.S.: [30] |  | *All participation factors:* **MSD =** 2 [30, 33] |
| *Role limitations (physical):*  **MSD =** 1-/2; Neg.: [30]; Pos.: none; N.S.: [48] |  | *Quality of life:* **CMD =** 1 [44] |
| Work related environmental (organizational) factors: Task contents | | |
| ***All job demands - physical factors:***  **MSDs =** 12-/21; Neg.: [26, 36, 50-54]; Pos: none; N.S.: [9-11, 55-58] | ***All job demands factors:***  **MSDs** = 19/40-; Neg.: [1-7, 16]; Pos.: none; N.S.: [7-14] | *Job demands (awkward postures):*  **MSDs** = 1 [56] |
| *Job control:*  **CMDs** = 2+/4; Neg.: none ; Pos.: [59, 60]; N.S.: [61, 62] | ***All job demands - psychological factors:***  **MSDs** = 3/9-; Neg.:[54, 59, 61] ; Pos.: none; N.S.: [54, 55] | *Job demands (prolonged sitting):*  **MSDs** = 1 [56] |
| *Job demands (general):*  **CMDs** = 1-/2; Neg.:[21]; Pos.: none; N.S.: [62] | *Job control:*  **MSDs** = 3+/10; Neg.: none; Pos.: [54, 59]; N.S.: [54, 55, 61, 63] |  |
|  | *Job demands (forceful work):*  **MSDs** = 1-/3; Neg.: [53]; Pos: none; N.S.: [9, 58] |  |
|  | *Job demands (general):*  **MSDs** = 4-/10; Neg.: [26, 36, 50-52, 54]; Pos.: none; N.S.: [10, 11, 55-57] |  |
|  | *Job demands (psychological):*  **MSDs** = 3-/9; Neg.:[54, 59, 61] ; Pos.: none; N.S.: [54, 55] |  |
|  | *Job demands (physical):*  **CMDs** = 1-/3; Neg.:[52]; Pos: none; N.S.: [10, 60] |  |
| Work related environmental (organizational) factors: Terms of employment | | |
|  |  | ***All Terms employment factors:***  **MSDs** = 3; [10, 55, 64] |
|  |  | *Terms employment (contract):*  **MSDs** = 2; [10, 64] |
|  |  | *Terms employment (working time arrangements):* **MSDs** = 1; [55] |
|  |  | *Work accommodations (workload):*  **MSDs** = 1 [27] |
| Work related environmental (organizational) factors: Social relationships at work | | |
| *Social support (mix of work and outside):*  **MSDs** = 1+/2; Neg.: none; Pos.:[65]; N.S.:[46] | ***All social support factors:***  **MSDs** = 5/18+; Neg.: none; Pos.: [21] [61, 66, 67] [24]; N.S.: [21, 50, 68-71] [72, 73] [46] [10, 64, 73]  **CMDs** *=* 2/9+; Neg.: none; Pos.: [61] [16]; N.S.: [10, 19, 21, 60] | ***All communication factors:***  **MSDs** = 2 [74] [73] |
| *Social support (supervisor and coworkers):*  **MSDs** = 3+/6; Neg.: none; Pos.: [61, 66, 67]; N.S.: [10, 64, 73]  **CMDs** = 1+/2; Neg.: none; Pos.:[61]; N.S.:[10] |  | *Communication (early contact):*  **MSDs** = 1 [74]  **CMDs** = 1 [18] |
| *Social support (outside work)*:  **MSDs** = 1+/2; Neg.: none; Pos.: [73]; N.S.: [71] |  | *Communication stakeholders (worker/employer):* **MSDs** = 1 [73] |
|  |  | *Conflicts (coworkers):*  **MSDs** = 1 [75] |
|  |  | *Conflicts (supervisor):*  **MSDs** = 2 [75] |
|  |  | *Social support (coworkers):*  **MSDs** = 5 [21, 50, 68-70]  **CMDs** = 3 [19, 21, 60] |
|  |  | *Social support (employer):* **MSDs** = 2 [72, 73] |
|  |  | *Social support (supervisor):* **CMDs** = 3 [19, 21, 60] |
| Work related environmental (organizational) factors: Working conditions | | |
|  |  |  |
| Work related environmental (organizational) factors: Characteristics of company | | |
| *Organization policies:*  **MSDs** = 2+/4; Neg.: none; Pos.: [11, 68]; N.S.: [64, 73] |  | *Insurance:*  **CMDs** = 1 [21] |
| Work related personal factors | | |
| *Job / work absence relationship (past):*  **CMDs** = 1-/2; Neg.: [76]; Pos.: none; N.S.: [77] | ***All job attitudes factors:***  **MSDs** =1-/1+/13; Neg.: [63]; Pos.: [65]; N.S.:[14, 25, 64, 68, 73, 75] [76] [21] | ***All job attitudes factors:***  **CMDs = 2**  Neg.: none; Pos.: none; N.S.: [21, 76] |
| *Work ability:*  **CMDs** = 2+/4; Neg.: none; Pos.: [62, 78]; N.S.: [76, 79] | *Job attitudes (insecurity):*  **MSDs** = 1-/4; Neg.:[63]; Pos.: none; N.S.: [68, 75, 76] | *Injustice (organizational)*:  **CMDs** = 1 [21] |
| *Self-efficacy (RTW):*  **CMDs** = 2+/4; Neg.: none; Pos.:[18, 21]; N.S.: [10, 80] |  | *Job attitudes (insecurity):*  **CMDs** = 1 [76] |
|  |  | *Job attitudes (job satisfaction):*  **MSDs** = 7 [14, 25, 64, 68, 73, 75] |
|  |  | *Job attitudes (motivation to RTW):*  **MSDs** = 1 [21]  **CMDs** = 1 [21] |
|  |  | *Job / work absence relationship (past):*  **MSDs** = 1 [76] |
|  |  | *Perceived relation between job and health:*  **CMDs** =1 [78] |
|  |  | *Satisfaction (job):* **MSDs** = 1[33] |
|  |  | *Work-life balance:*  **MSDs** = 1 [21]  **CMDs** = 2 [21, 60] |
| Personal factors: General ‘mental’ personal factors / psychological assets | | |
| *Coping (strategies):*  **MSD** = 1-/2; Neg.: [81]; Pos.: none; N.S.: [10] | *Fear (Tampa Scale of Kinesiophobia):*  **MSD =** 1-/3; Neg.: [32]; Pos.: none; N.S.: [2, 13] | *Blame for injury*: **MSD =** 1 [43] |
| *Coping (style):*  **MSD & CMD** = 1-/2; Neg.: [81]; Pos.: none; N.S.: [10] | *Self-efficacy (General):*  **MSD =** 1+/4; Neg.: none; Pos.: [82]; N.S.: [44, 81, 83]  **CMD =** 1+/5; Neg.: none; Pos.: [82]; N.S.: [44, 81, 83, 84] | *Fear (Fear Avoidance Belief Questionnaire* - *Physical activities):*  **MSD =** 1 [81]; **CMD =**1 [81] |
| *All fear factors:*  **CMD =** 1-/2; Neg.: [81]; Pos.: none; N.S.: [21] |  | *Fear (Örebro Musculoskeletal Pain Screening Questionnaire):* **MSD =** 1 [7] |
| *Self-efficacy (pain):*  **MSD =** 1+/2; Neg.: none; Pos.: [85]; N.S.: [34] |  | *Fear (work/health interference):*  **CMD =** 1 [21] |
| *Vitality:*  **CMD =** 1+/2; Neg.: none; Pos.: [16]; N.S.: [30] |  | *Hopelessness:* **CMD =** 1 [81] |
|  |  | *Illness perceptions:* **CMD =** 1 [86] |
|  |  | *Sense of coherence:* **MSD =** 2 [46, 87] |
| Personal factors: Disease related factors (including comorbidity) | | |
| *Comorbidity:* **MSD =** 4-/8; Neg.: [9, 11, 38, 53]; Pos.: none; N.S.: [2, 8, 33, 81] | *General Health:* **MSD =** 4+/9; Neg.: none; Pos.: [1, 2, 83, 88]; N.S.: [22, 25, 30, 41, 89] | *Comorbidity:* **CMD =** 2 [81, 86] |
|  |  | *Medications:* **CMD =** 1 [21] |
| *Physical health:*  **MSD =** 1+/2; Neg.: none; Pos.: [5]; N.S.: [27] | *Mental Health:*  **MSD =** 5+/13; Neg.: none; Pos.: [1, 30, 32, 38, 41]; N.S.: [2, 43, 44, 48, 90], [28]-NLD, [28]-SWE, [28]-US ^b^  **CMD =** 1+/5; Neg.: none; Pos.: [30]; N.S.: [44, 48, 62, 86] | *Negative health change*: **CMD** = 1 [30] |
|  |  | *Perception of injury severity:* **MSD =** 1 [41] |
|  |  | *Lifestyle (hobbies):* **MSD =** 1 [6] |
|  |  | *Lifestyle (no smoking):* **MSD =** 3 [5, 8, 91] |
| Personal factors: Lifestyle (habits) | | |
|  | *All lifestyle factors:*  **MSD =** 3+/8; Neg.: none; Pos.: [3, 7, 48]; N.S.: [5, 6, 8, 26, 91] | *Multiple burden (housekeeping):* **MSD =** 1 [56] |
|  |  | *BMI/Weight:* **MSD =** 1 [8] |
| Unclassified | | |
|  | *Presence of RTW coordinator* *(can be in the work, healthcare or insurance system)*:  **MSD** = 1+/2; Neg.: none; Pos.:[73]; N.S.: [74, 92] | *Presence of RTW coordinator* *(can be in the work, healthcare or insurance system):*  **CMD** = 1[92] |

^a^ (+): statistically significant positive association; (-) statistically significant negative association; (N.S,) non statistically significant association. Rules to determine the level of evidence are reported in Figure 1. Example of interpretation: “Job demands (general) 1-/2”, seen in the “Inconsistent” evidence column, means that job demands (general) reached inconsistent evidence of a negative association with RTW (risk factor), as 1 out of the 2 studies assessing this factor showed this negative association.

**References**

1. Turner J, Franklin G, Fulton-Kehoe D, Sheppard L, Stover B, Wu R, et al. Early predictors of chronic work disability: a prospective, population-based study of workers with back injuries. Spine. 2008;33(25):2809-18.

2. Grøvle L, Haugen AJ, Keller A, Ntvig B, Brox JI, Grotle M. Prognostic factors for return to work in patients with sciatica. The Spine Journal: Official Journal of the North American Spine Society. 2013;13(12):1849-57. doi: 10.1016/j.spinee.2013.07.433.

3. Haldorsen EM, Indahl A, Ursin H. Patients with low back pain not returning to work. A 12-month follow-up study. Spine. 1998;23(11):1202-7.

4. Schultz IZ, Crook JM, Berkowitz J, Meloche GR, Milner R, Zuberbier OA, et al. Biopsychosocial multivariate predictive model of occupational low back disability. Spine. 2002;27(23):2720-5.

5. Katz J, Amick B, Keller R, Fossel A, Ossman J, Soucie V, et al. Determinants of work absence following surgery for carpal tunnel syndrome. American Journal of Industrial Medicine. 2005;47(2):120-30. doi: 10.1002/ajim.20127.

6. Bontoux L, Roquelaure Y, Billabert C, Dubus V, Sancho PO, Colin D, et al. [Prospective study of the outcome at one year of patients with chronic low back pain in a program of intensive functional restoration and ergonomic intervention. Factors predicting their return to work]. Annales de réadaptation et de médecine physique. 2004;47(8):563-72. doi: 10.1016/j.annrmp.2004.03.006.

7. Nicholas MK, Costa DSJ, Linton SJ, Main CJ, Shaw WS, Pearce R, et al. Predicting Return to Work in a Heterogeneous Sample of Recently Injured Workers Using the Brief ÖMPSQ-SF. J Occup Rehabil. 2019;29(2):295-302. Epub 2018/05/26. doi: 10.1007/s10926-018-9784-8. PubMed PMID: 29796980.

8. Asher AL, Devin CJ, Archer KR, Chotai S, Parker S, Bydon M, et al. An analysis from the Quality Outcomes Database, Part 2. Predictive model for return to work after elective surgery for lumbar degenerative disease. Journal of Neurosurgery Spine. 2017:1-12. doi: 10.3171/2016.8.SPINE16527.

9. Hagen EM, Svensen E, Eriksen HR. Predictors and modifiers of treatment effect influencing sick leave in subacute low back pain patients. Spine (Phila Pa 1976). 2005;30(24):2717-23. PubMed PMID: 16371893.

10. Huijs JJ, Koppes LL, Taris TW, Blonk RW. Differences in predictors of return to work among long-term sick-listed employees with different self-reported reasons for sick leave. J Occup Rehabil. 2012;22(3):301-11. Epub 2012/02/04. doi: 10.1007/s10926-011-9351-z. PubMed PMID: 22302668.

11. Amick BC, 3rd, Lee H, Hogg-Johnson S, Katz JN, Brouwer S, Franche RL, et al. How Do Organizational Policies and Practices Affect Return to Work and Work Role Functioning Following a Musculoskeletal Injury? J Occup Rehabil. 2017;27(3):393-404. Epub 2016/09/23. doi: 10.1007/s10926-016-9668-8. PubMed PMID: 27654622.

12. Carriere JS, Thibault P, Sullivan MJ. The Mediating Role of Recovery Expectancies on the Relation Between Depression and Return-to-Work. J Occup Rehabil. 2015;25(2):348-56. doi: 10.1007/s10926-014-9543-4. PubMed PMID: 25252609.

13. Gauthier N, Sullivan MJ, Adams H, Stanish WD, Thibault P. Investigating risk factors for chronicity: the importance of distinguishing between return-to-work status and self-report measures of disability. JOccupEnvironMed. 2006;48(3):312-8.

14. Steenstra IA, Franche RL, Furlan AD, Amick B, 3rd, Hogg-Johnson S. The Added Value of Collecting Information on Pain Experience When Predicting Time on Benefits for Injured Workers with Back Pain. J Occup Rehabil. 2016;26(2):117-24. doi: 10.1007/s10926-015-9592-3. PubMed PMID: 26152837.

15. Truchon M, Côté D. Predictive validity of the Chronic Pain Coping Inventory in subacute low back pain. Pain. 2005;116(3):205-12.

16. Gustafsson K, Lundh G, Svedberg P, Linder J, Alexanderson K, Marklund S. Psychological factors are related to return to work among long-term sickness absentees who have undergone a multidisciplinary medical assessment. J Rehabil Med. 2013;45(2):186-91. doi: 10.2340/16501977-1077. PubMed PMID: 23138390.

17. Nielsen MB, Bultmann U, Madsen IE, Martin M, Christensen U, Diderichsen F, et al. Health, work, and personal-related predictors of time to return to work among employees with mental health problems. Disabil Rehabil. 2012;34(15):1311-6. Epub 2011/12/28. doi: 10.3109/09638288.2011.641664. PubMed PMID: 22200251.

18. Lagerveld SE, Brenninkmeijer V, Blonk RW, Twisk J, Schaufeli WB. Predictive value of work-related self-efficacy change on RTW for employees with common mental disorders. Occup Environ Med. 2017;74(5):381-3.

19. Nieuwenhuijsen K, Verbeek JH, de Boer AG, Blonk RW, van Dijk FJ. Predicting the duration of sickness absence for patients with common mental disorders in occupational health care. Scand J Work Environ Health. 2006;32(1):67-74. PubMed PMID: 16539174.

20. Lepiece B, Reynaert C, Jacques D, Zdanowicz N. Returning to Work after a Common Mental Health Disorder: a New Preoccupation for Mental Health Professionals? Psychiatr Danub. 2017;29(Suppl 3):262-6. Epub 2017/09/28. PubMed PMID: 28953774.

21. Corbiere M, Negrini A, Durand MJ, St-Arnaud L, Briand C, Fassier JB, et al. Development of the Return-to-Work Obstacles and Self-Efficacy Scale (ROSES) and Validation with Workers Suffering from a Common Mental Disorder or Musculoskeletal Disorder. J Occup Rehabil. 2017;27(3):329-41. doi: 10.1007/s10926-016-9661-2. PubMed PMID: 27562583.

22. Lötters F, Burdorf A. Prognostic factors for duration of sickness absence due to musculoskeletal disorders. The Clinical Journal of Pain. 2006;22(2):212-21.

23. Lydell M, Grahn B, Månsson J, Baigi A, Marklund B. Predictive factors of sustained return to work for persons with musculoskeletal disorders who participated in rehabilitation. Work (Reading, Mass). 2009;33(3):317-28. doi: 10.3233/WOR-2009-0879.

24. Westman A, Linton SJ, Öhrvik J, Wahlén P, Leppert J. Do psychosocial factors predict disability and health at a 3-year follow-up for patients with non-acute musculoskeletal pain?: a validation of the Örebro Musculoskeletal Pain Screening Questionnaire. European Journal of Pain. 2008;12(5):641-9.

25. Beemster TT, van Bennekom CAM, van Velzen JM, Frings-Dresen MHW, Reneman MF. Vocational Rehabilitation with or without Work Module for Patients with Chronic Musculoskeletal Pain and Sick Leave from Work: Longitudinal Impact on Work Participation. J Occup Rehabil. 2021;31(1):72-83. Epub 2020/05/08. doi: 10.1007/s10926-020-09893-z. PubMed PMID: 32378023; PubMed Central PMCID: PMC7954725.

26. Reme SE, Hagen EM, Eriksen HR. Expectations, perceptions, and physiotherapy predict prolonged sick leave in subacute low back pain. BMC Musculoskelet Disord. 2009;10:139. Epub 2009/11/17. doi: 10.1186/1471-2474-10-139. PubMed PMID: 19912626; PubMed Central PMCID: PMC2780378.

27. van Duijn M, Lotters F, Burdorf A. Influence of modified work on return to work for employees on sick leave due to musculoskeletal complaints. J Rehabil Med. 2005;37(3):172-9. doi: 10.1080/16501970410023434.

28. Hansson TH, Hansson EK. The effects of common medical interventions on pain, back function, and work resumption in patients with chronic low back pain: A prospective 2-year cohort study in six countries. Spine. 2000;25(23):3055-64.

29. Adams H, Thibault P, Ellis T, Moore E, Sullivan M. The Relation Between Catastrophizing and Occupational Disability in Individuals with Major Depression: Concurrent and Prospective Associations. J Occup Rehabil. 2017;27(3):405-12. Epub 2016/10/23. doi: 10.1007/s10926-016-9669-7. PubMed PMID: 27770242.

30. Post M, Krol B, Groothoff JW. Self-rated health as a predictor of return to work among employees on long-term sickness absence. Disabil Rehabil. 2006;28(5):289-97. Epub 2006/02/24. doi: 10.1080/09638280500160303. PubMed PMID: 16492623.

31. Du Bois M, Donceel P. A screening questionnaire to predict no return to work within 3 months for low back pain claimants. European Spine Journal. 2008;17(3):380-5. doi: 10.1007/s00586-007-0567-8.

32. Du Bois M, Szpalski M, Donceel P. Patients at risk for long-term sick leave because of low back pain. Spine J. 2009;9(5):350-9. doi: 10.1016/j.spinee.2008.07.003. PubMed PMID: 18790677.

33. Cougot B, Petit A, Paget C, Roedlich C, Fleury-Bahi G, Fouquet M, et al. Chronic low back pain among French healthcare workers and prognostic factors of return to work (RTW): a non-randomized controlled trial. J Occup Med Toxicol. 2015;10:40. doi: 10.1186/s12995-015-0082-5. PubMed PMID: 26516339; PubMed Central PMCID: PMCPMC4625968.

34. Storheim K, Brox JI, Holm I, Bo K. Predictors of return to work in patients sick listed for sub-acute low back pain: a 12-month follow-up study. J Rehabil Med. 2005;37(6):365-71. doi: 10.1080/16501970510040344.

35. Koopman FS, Edelaar M, Slikker R, Reynders K, van der Woude LH, Hoozemans MJ. Effectiveness of a multidisciplinary occupational training program for chronic low back pain: a prospective cohort study. Am J Phys Med Rehabil. 2004;83(2):94-103. doi: 10.1097/01.PHM.0000107482.35803.11. PubMed PMID: 14758295.

36. Bosman LC, Twisk JWR, Geraedts AS, Heymans MW. Development of Prediction Model for the Prognosis of Sick Leave Due to Low Back Pain. J Occup Environ Med. 2019;61(12):1065-71. Epub 2019/10/28. doi: 10.1097/jom.0000000000001749. PubMed PMID: 31651601.

37. Faber E, Burdorf A, Bierma-Zeinstra SM, Miedema HS, Koes BW. Determinants for improvement in different back pain measures and their influence on the duration of sickness absence. Spine. 2006;31(13):1477-83.

38. Opsahl J, Eriksen HR, Tveito TH. Do expectancies of return to work and Job satisfaction predict actual return to work in workers with long lasting LBP? BMC Musculoskeletal Disorders. 2016;17. doi: 10.1186/s12891-016-1314-2.

39. Hogg-Johnson S, Cole D. Early prognostic factors for duration on temporary total benefits in the first year among workers with compensated occupational soft tissue injuries. Occupational and Environmental Medicine. 2003;60(4):244-53.

40. Baltov P, Cote J, Truchon M, Feldman DE. Psychosocial and socio-demographic factors associated with outcomes for patients undergoing rehabilitation for chronic whiplash associated disorders: a pilot study. DisabilRehabil. 2008;30(25):1947-55.

41. Schultz IZ, Crook J, Berkowitz J, Milner R, Meloche GR. Predicting Return to Work After Low Back Injury Using the Psychosocial Risk for Occupational Disability Instrument: A Validation Study. Journal of Occupational Rehabilitation. 2005;15(3):365-76. doi: 10.1007/s10926-005-5943-9.

42. Iles R, Sheehan L, Munk K, Gosling C. Development and Pilot Assessment of the PACE Tool: Helping Case Managers Identify and Respond to Risk Factors in Workers' Compensation Case Management. J Occup Rehabil. 2020;30(2):167-82. doi: 10.1007/s10926-019-09858-x. PubMed PMID: 31541425.

43. Turner JA, Franklin G, Fulton-Kehoe D, Sheppard L, Wickizer TM, Wu R, et al. Worker recovery expectations and fear-avoidance predict work disability in a population-based workers' compensation back pain sample. Spine. 2006;31(6):682-9.

44. Wahlin C, Ekberg K, Persson J, Bernfort L, Oberg B. Association between clinical and work-related interventions and return-to-work for patients with musculoskeletal or mental disorders. J Rehabil Med. 2012;44(4):355-62.

45. Laukkala T, Heikinheimo S, Vuokko A, Junttila IS, Tuisku K. Subjective and objective measures of function and return to work: an observational study with a clinical psychiatric cohort. Soc Psychiatry Psychiatr Epidemiol. 2018;53(5):537-40. Epub 2017/12/25. doi: 10.1007/s00127-017-1479-5. PubMed PMID: 29275503.

46. Lydell M, Marklund B, Baigi A, Mattsson B, Mansson J. Return or no return--psychosocial factors related to sick leave in persons with musculoskeletal disorders: a prospective cohort study. Disabil Rehabil. 2011;33(8):661-6. doi: 10.3109/09638288.2010.506237. PubMed PMID: 20690859.

47. Steenstra IA, Koopman FS, Knol DL, Kat E, Bongers PM, de Vet HC, et al. Prognostic factors for duration of sick leave due to low-back pain in dutch health care professionals. J Occup Rehabil. 2005;15(4):591-605. doi: 10.1007/s10926-005-8037-9. PubMed PMID: 16254758.

48. Hara KW, Bjorngaard JH, Jacobsen HB, Borchgrevink PC, Johnsen R, Stiles TC, et al. Biopsychosocial predictors and trajectories of work participation after transdiagnostic occupational rehabilitation of participants with mental and somatic disorders: a cohort study. BMC Public Health. 2018;18(1):1014. doi: 10.1186/s12889-018-5803-0. PubMed PMID: 30111291; PubMed Central PMCID: PMCPMC6094579.

49. Gross DP, Battie MC, Cassidy JD. The prognostic value of functional capacity evaluation in patients with chronic low back pain: part 1: timely return to work. Spine. 2004;29(8):914-9.

50. Lötters F, Burdorf A. Prognostic factors for duration of sickness absence due to musculoskeletal disorders. Clin J Pain. 2006;22(2):212-21. Epub 2006/01/24. doi: 10.1097/01.ajp.0000154047.30155.72. PubMed PMID: 16428958.

51. Steenstra IA, Busse JW, Tolusso D, Davilmar A, Lee H, Furlan AD, et al. Predicting time on prolonged benefits for injured workers with acute back pain. J Occup Rehabil. 2015;25(2):267-78. Epub 2014/08/29. doi: 10.1007/s10926-014-9534-5. PubMed PMID: 25164779; PubMed Central PMCID: PMC4436678.

52. Hara KW, Bjørngaard JH, Jacobsen HB, Borchgrevink PC, Johnsen R, Stiles TC, et al. Biopsychosocial predictors and trajectories of work participation after transdiagnostic occupational rehabilitation of participants with mental and somatic disorders: a cohort study. BMC Public Health. 2018;18(1):1014. doi: 10.1186/s12889-018-5803-0.

53. Nordin M, Hiebert R, Pietrek M, Alexander M, Crane M, Lewis S. Association of comorbidity and outcome in episodes of nonspecific low back pain in occupational populations. J Occup Environ Med. 2002;44(7):677-84. Epub 2002/07/24. doi: 10.1097/00043764-200207000-00015. PubMed PMID: 12134532.

54. Hansson TH, Hansson EK. The effects of common medical interventions on pain, back function, and work resumption in patients with chronic low back pain: A prospective 2-year cohort study in six countries. Spine (Phila Pa 1976). 2000;25(23):3055-64. Epub 2001/01/06. doi: 10.1097/00007632-200012010-00013. PubMed PMID: 11145817.

55. Storheim K, Brox, J. I., Holm, I., & Bo, K. . Predictors of return to work in patients sick listed for sub-acute low back pain: a 12-month follow-up study. Journal of rehabilitation medicine. 2005;37(6):365-71. doi: <https://doi.org/10.1080/16501970510040344>.

56. Abásolo L, Carmona L, Lajas C, Candelas G, Blanco M, Loza E, et al. Prognostic factors in short-term disability due to musculoskeletal disorders. Arthritis Rheum. 2008;59(4):489-96. Epub 2008/04/03. doi: 10.1002/art.23537. PubMed PMID: 18383421.

57. Gaines WG, Jr., Hegmann KT. Effectiveness of Waddell's nonorganic signs in predicting a delayed return to regular work in patients experiencing acute occupational low back pain. Spine (Phila Pa 1976). 1999;24(4):396-400; discussion 1. Epub 1999/03/05. doi: 10.1097/00007632-199902150-00021. PubMed PMID: 10065525.

58. Turner JA, Franklin G, Fulton-Kehoe D, Sheppard L, Stover B, Wu R, et al. ISSLS prize winner: early predictors of chronic work disability: a prospective, population-based study of workers with back injuries. Spine (Phila Pa 1976). 2008;33(25):2809-18. Epub 2008/12/04. doi: 10.1097/BRS.0b013e31817df7a7. PubMed PMID: 19050587.

59. Haveraaen LA, Skarpaas LS, Aas RW. Job demands and decision control predicted return to work: the rapid-RTW cohort study. BMC Public Health. 2017;17(1):154. doi: 10.1186/s12889-016-3942-8.

60. Norder G, Roelen CAM, van der Klink JJL, Bültmann U, Sluiter JK, Nieuwenhuijsen K. External Validation and Update of a Prediction Rule for the Duration of Sickness Absence Due to Common Mental Disorders. J Occup Rehabil. 2017;27(2):202-9. Epub 2016/06/05. doi: 10.1007/s10926-016-9646-1. PubMed PMID: 27260170; PubMed Central PMCID: PMC5405096.

61. Haveraaen LA, Skarpaas LS, Berg JE, Aas RW. Do psychological job demands, decision control and social support predictreturn to work three months after a return-to-work (RTW) programme? The rapid-RTW cohort study. Work. 2015;53 1:61-71.

62. Netterstrom B, Eller NH, Borritz M. Prognostic Factors of Returning to Work after Sick Leave due to Work-Related Common Mental Disorders: A One- and Three-Year Follow-Up Study. Biomed Res Int. 2015;2015:596572. doi: 10.1155/2015/596572. PubMed PMID: 26557678; PubMed Central PMCID: PMCPMC4628746.

63. Schultz IZ, Crook JM, Berkowitz J, Meloche GR, Milner R, Zuberbier OA, et al. Biopsychosocial multivariate predictive model of occupational low back disability. Spine (Phila Pa 1976). 2002;27(23):2720-5. Epub 2002/12/04. doi: 10.1097/00007632-200212010-00012. PubMed PMID: 12461399.

64. Soucy I, Truchon M, Côté D. Work-related factors contributing to chronic disability in low back pain. Work. 2006;26(3):313-26. Epub 2006/05/25. PubMed PMID: 16720972.

65. Brouwer S, Reneman MF, Bültmann U, van der Klink JJ, Groothoff JW. A prospective study of return to work across health conditions: perceived work attitude, self-efficacy and perceived social support. J Occup Rehabil. 2010;20(1):104-12. Epub 2009/11/07. doi: 10.1007/s10926-009-9214-z. PubMed PMID: 19894106; PubMed Central PMCID: PMC2832875.

66. Baltov P, Côte J, Truchon M, Feldman DE. Psychosocial and socio-demographic factors associated with outcomes for patients undergoing rehabilitation for chronic whiplash associated disorders: a pilot study. Disabil Rehabil. 2008;30(25):1947-55. Epub 2008/07/09. doi: 10.1080/09638280701791245. PubMed PMID: 18608396.

67. Opsahl J, Eriksen HR, Tveito TH. Do expectancies of return to work and Job satisfaction predict actual return to work in workers with long lasting LBP? BMC Musculoskelet Disord. 2016;17(1):481. Epub 2016/11/20. doi: 10.1186/s12891-016-1314-2. PubMed PMID: 27855684; PubMed Central PMCID: PMC5114779.

68. Katz JN, Amick BC, 3rd, Keller R, Fossel AH, Ossman J, Soucie V, et al. Determinants of work absence following surgery for carpal tunnel syndrome. Am J Ind Med. 2005;47(2):120-30. Epub 2005/01/22. doi: 10.1002/ajim.20127. PubMed PMID: 15662641.

69. Schultz IZ, Crook J, Meloche GR, Berkowitz J, Milner R, Zuberbier OA, et al. Psychosocial factors predictive of occupational low back disability: towards development of a return-to-work model. Pain. 2004;107(1-2):77-85. Epub 2004/01/13. doi: 10.1016/j.pain.2003.09.019. PubMed PMID: 14715392.

70. Turner JA, Franklin G, Fulton-Kehoe D, Sheppard L, Wickizer TM, Wu R, et al. Worker recovery expectations and fear-avoidance predict work disability in a population-based workers' compensation back pain sample. Spine (Phila Pa 1976). 2006;31(6):682-9. Epub 2006/03/17. doi: 10.1097/01.brs.0000202762.88787.af. PubMed PMID: 16540874.

71. Rashid M, Kristofferzon ML, Nilsson A. Predictors of return to work among women with long-term neck/shoulder and/or back pain: A 1-year prospective study. PLoS One. 2021;16(11):e0260490. Epub 2021/11/24. doi: 10.1371/journal.pone.0260490. PubMed PMID: 34813601; PubMed Central PMCID: PMC8610267.

72. Schultz IZ, Crook J, Berkowitz J, Milner R, Meloche GR. Predicting return to work after low back injury using the Psychosocial Risk for Occupational Disability Instrument: a validation study. J Occup Rehabil. 2005;15(3):365-76. Epub 2005/08/27. doi: 10.1007/s10926-005-5943-9. PubMed PMID: 16119227.

73. Iles RA, Sheehan LR, Gosling CM. Assessment of a new tool to improve case manager identification of delayed return to work in the first two weeks of a workers' compensation claim. Clin Rehabil. 2020;34(5):656-66. Epub 2020/03/19. doi: 10.1177/0269215520911417. PubMed PMID: 32183561.

74. Franche R-L, Severin CN, Hogg-Johnson S, Côté P, Vidmar M, Lee H. The impact of early workplace-based return-to-work strategies on work absence duration: a 6-month longitudinal study following an occupational musculoskeletal injury. Journal of occupational and environmental medicine. 2007;49(9):960-74. doi: 10.1097/jom.0b013e31814b2e9f. PubMed PMID: 17848852.

75. Fishbain DA, Cutler RB, Rosomoff HL, Khalil T, Steele-Rosomoff R. Impact of chronic pain patients' job perception variables on actual return to work. Clin J Pain. 1997;13(3):197-206. Epub 1997/09/26. doi: 10.1097/00002508-199709000-00004. PubMed PMID: 9303251.

76. Sampere M, Gimeno D, Serra C, Plana M, López JC, Martínez JM, et al. Return to work expectations of workers on long-term non-work-related sick leave. J Occup Rehabil. 2012;22(1):15-26. Epub 2011/06/28. doi: 10.1007/s10926-011-9313-5. PubMed PMID: 21701951.

77. Løvvik C, Shaw W, Overland S, Reme SE. Expectations and illness perceptions as predictors of benefit recipiency among workers with common mental disorders: secondary analysis from a randomised controlled trial. BMJ Open. 2014;4(3):e004321. Epub 2014/03/05. doi: 10.1136/bmjopen-2013-004321. PubMed PMID: 24589824; PubMed Central PMCID: PMC3948454.

78. Victor M, Lau B, Ruud T. Predictors of Return to Work 6 Months After the End of Treatment in Patients with Common Mental Disorders: A Cohort Study. J Occup Rehabil. 2018;28(3):548-58. Epub 2017/12/14. doi: 10.1007/s10926-017-9747-5. PubMed PMID: 29234955; PubMed Central PMCID: PMC6096513.

79. Wåhlin C, Ekberg K, Persson J, Bernfort L, Oberg B. Association between clinical and work-related interventions and return-to-work for patients with musculoskeletal or mental disorders. J Rehabil Med. 2012;44(4):355-62. Epub 2012/03/22. doi: 10.2340/16501977-0951. PubMed PMID: 22434378.

80. Hedlund Å, Nilsson A, Boman E, Kristofferzon ML. Predictors of return to work and psychological well-being among women during/after long-term sick leave due to common mental disorders - a prospective cohort study based on the theory of planned behaviour. Health Soc Care Community. 2022;30(6):e5245-e58. Epub 2022/07/28. doi: 10.1111/hsc.13943. PubMed PMID: 35894151; PubMed Central PMCID: PMC10087653.

81. Oyeflaten I, Hysing M, Eriksen HR. Prognostic factors associated with return to work following multidisciplinary vocational rehabilitation. J Rehabil Med. 2008;40(7):548-54. doi: 10.2340/16501977-0202. PubMed PMID: 18758672.

82. Brouwer S, Reneman MF, Bultmann U, van der Klink JJ, Groothoff JW. A prospective study of return to work across health conditions: perceived work attitude, self-efficacy and perceived social support. J Occup Rehabil. 2010;20(1):104-12. doi: 10.1007/s10926-009-9214-z. PubMed PMID: 19894106; PubMed Central PMCID: PMCPMC2832875.

83. Sampere M, Gimeno D, Serra C, Plana M, Lopez JC, Martinez JM, et al. Return to work expectations of workers on long-term non-work-related sick leave. Journal of Occupational Rehabilitation. 2012;22(1):15-26. doi: 10.1007/s10926-011-9313-5.

84. Victor M, Lau B, Ruud T. Predictors of return to work among patients in treatment for common mental disorders: a pre-post study. BMC Public Health. 2017;18(1):27. doi: 10.1186/s12889-017-4581-4. PubMed PMID: 28720129; PubMed Central PMCID: PMCPMC5516307.

85. Brouwer S, Amick BC, 3rd, Lee H, Franche RL, Hogg-Johnson S. The Predictive Validity of the Return-to-Work Self-Efficacy Scale for Return-to-Work Outcomes in Claimants with Musculoskeletal Disorders. J Occup Rehabil. 2015;25(4):725-32. doi: 10.1007/s10926-015-9580-7. PubMed PMID: 25990375; PubMed Central PMCID: PMCPMC4636988.

86. Løvvik C, Shaw W, Øverland S, Reme SE. Expectations and illness perceptions as predictors of benefit recipiency among workers with common mental disorders: secondary analysis from a randomised controlled trial. BMJ Open. 2014;4(3):e004321. doi: 10.1136/bmjopen-2013-004321.

87. Lillefjell M, Jakobsen K. Sense of coherence as a predictor of work reentry following multidisciplinary rehabilitation for individuals with chronic musculoskeletal pain. Journal of Occupational Health Psychology. 2007;12(3):222-31. doi: 10.1037/1076-8998.12.3.222.

88. Selander J, Marnetoft SU, Asell M. Predictors for successful vocational rehabilitation for clients with back pain problems. Disabil Rehabil. 2007;29(3):215-20. doi: 10.1080/09638280600756208. PubMed PMID: 17364772.

89. Kvam L, Vik K, Eide AH. Importance of participation in major life areas matters for return to work. J Occup Rehabil. 2015;25(2):368-77. doi: 10.1007/s10926-014-9545-2. PubMed PMID: 25319539; PubMed Central PMCID: PMCPMC4436658.

90. Lindell O, Johansson S-E, Strender L-E. Predictors of stable return-to-work in non-acute, non-specific spinal pain: low total prior sick-listing, high self prediction and young age. A two-year prospective cohort study. BMC Fam Pract. 2010;11:53. doi: 10.1186/1471-2296-11-53.

91. Gaines Jr WG, Hegmann KT. Effectiveness of Waddell's nonorganic signs in predicting a delayed return to regular work in patients experiencing acute occupational low back pain. Spine. 1999;24(4):396-400.

92. Skarpaas LS, Haveraaen LA, Småstuen MC, Shaw WS, Aas RW. The association between having a coordinator and return to work: the rapid-return-to-work cohort study. BMJ Open. 2019;9(2):e024597. doi: 10.1136/bmjopen-2018-024597.
