# Supplementary material for: Systematic review and tools appraisal of prognostic factors of return to work in workers on sick leave due to musculoskeletal and common mental disorders
Source: PLoS One. 2024 Jul 17;19(7):e0307284. doi: 10.1371/journal.pone.0307284 (PMC11253986; doi:10.1371/journal.pone.0307284)
Supplement: S5 File — (DOCX) [file pone.0307284.s005.docx]

**Supplementary material 5**

**Table S28**. Tools appraisal using psychometric (n = 6) and usability (n = 4) criteria

| Prognostic factor name   - Tool (items) / question 1 (references for psychometric properties) - Tool (items) / question 2 - Tool (items) / question … | Population | Psychometric criteria | | | | | | |  | Usability criteria ^a^ | | | | | | Overall classification ^b^ |
| --- | --- | --- | --- | --- | --- | --- | --- | --- | --- | --- | --- | --- | --- | --- | --- | --- |
|  |  | 1. Face validity | 2.Construct validity | 3.Convergent validity | 4. Internal consistency | 5. Test-retest reliability | 6.Predictive validity | Psychometric score (/6) |  | 1. Time | 2. Administration | 3. Interpretation | 4. Accessibility | Usability score (/4) |  | |
| *Work accommodations (offer/availability/feasibility)*: strong evidence for MSDs |  |  |  |  |  |  |  |  |  |  |  |  |  |  |  | |
| T1 - Single item (Respondents were asked whether their workplace had made any offers of special arrangements to help them return to work) [1] | MSD | √ |  |  |  |  | √ | 2/6 |  | √ | √ | √ | √ | 4/4 | Q | |
| T2 - Two items:1) Have you been offered work accommodation? and 2) Did you accept the work accommodation offer? [2] | MSD | √ |  |  |  |  | √ | 2/6 |  | √ | √ | √ | √ | 4/4 | Q | |
| T3 - Single item stemming from the Plan of Action for a Case (PACE) tool (41 items in total). The item is the following: Is the employer able to provide suitable duties? [3] | MSD | √ |  |  |  |  | √ | 2/6 |  | √ | √ | √ | √ | 4/4 | Q | |
| T4 - Single item asking respondents if the employer offered job accommodation [4] | MSD | √ |  |  |  |  | √ | 2/6 |  | √ | √ | √ | √ | 4/4 | Q | |
| T5 - Single item asking whether an ergonomic assessment and recommendations have been carried out at the workplace | MSD | √ |  |  |  |  | √ | 2/6 |  | √ | √ | √ | √ | 4/4 | Q | |
| *Expectations (RTW)*: strong evidence for MSDs and CMDs |  |  |  |  |  |  |  |  |  |  |  |  |  |  |  | |
| T6 - Single item (certainty that they will be working in six months) [5] [4, 6] | MSD | √ |  |  |  |  | √ | 2/6 |  | √ | √ | √ | √ | 4/4 | Q | |
| T7 - Single item from the Örebro Musculoskeletal Pain Screening Questionnaire (item 16: Do you expect to return to work within 6 months?) [7] [8] | MSD | √ |  |  |  |  | √ | 2/6 |  | √ | √ | √ | √ | 4/4 | Q | |
| T8 - Single item (intent to return to preinjury type of work) [9] | MSD | √ |  |  |  |  | √ | 2/6 |  | √ | √ | √ | √ | 4/4 | Q | |
| T9 - Work-Related Recovery Expectations Questionnaire (3 items) [10] [11] | MSD | √ |  |  | √ |  | √ | 3/6 |  | √ | √ |  | √ | 3/4 | G | |
| T10 - Single item from the Fear Avoidance Beliefs Questionnaire (FABQ), work subscale: I do not think that I will be back in my ordinary work within 3 months [12] | MSD/CMD | √ |  |  |  |  | √ | 2/6 |  | √ | √ | √ | √ | 4/4 | Q | |
| T11 - Single item (estimation of chances to be working normal duties in 3 months) from the Örebro Musculoskeletal Pain Screening Questionnaire- short form (ÖMPSQ-SF) (10 items) [13] | MSD | √ |  |  |  |  | √ | 2/6 |  | √ | √ | √ | √ | 4/4 | Q | |
| T12 - Single item (expectation to return to work within the next few weeks) [14] | MSD | √ |  |  |  |  | √ | 2/6 |  | √ | √ | √ | √ | 4/4 | Q | |
| T13 - Single item (Approximately how long do you think you will need to return to the job you had before you went on sick leave?) [15] | MSD/CMD | √ |  |  |  |  | √ | 2/6 |  | √ | √ | √ | √ | 4/4 | Q | |
| T14 - Single item (expected duration of sick leave) [16] | MSD | √ |  |  |  |  | √ | 2/6 |  | √ | √ | √ | √ | 4/4 | Q | |
| T15 - Single item (chances to be working in 6 months?) [17] | MSD | √ |  |  |  |  | √ | 2/6 |  | √ | √ | √ | √ | 4/4 | Q | |
| T61 - Single item (I expect to be back at work within the next few weeks) [18] | CMD | √ |  |  |  |  | √ | 2/6 |  | √ | √ | √ | √ | 4/4 | Q | |
| T62 - Single item (Do you expect to be able to return to your workplace?) [19] | CMD | √ |  |  |  |  | √ | 2/6 |  | √ | √ | √ | √ | 4/4 | Q | |
| T63 - Single item (How many months do you think it will take you to fully return to work?) [20] | CMD | √ |  |  |  |  | √ | 2/6 |  | √ | √ | √ | √ | 4/4 | Q | |
| T64 - RTW intention subscale of the RTW Beliefs Questionnaire (three items on expectation to, willing to, and intention to RTW within three months) [21, 22] | CMD | √ |  | √ | √ |  | √ | 4/6 |  | √ | √ | √ | √ | 4/4 | E | |
| *Fear (FABQ-W)*: Strong evidence for MSDs |  |  |  |  |  |  |  |  |  |  |  |  |  |  |  | |
| T16 - Work subscale (7 items) of the FABQ [23-28] | MSD | √ | √ | √ | √ | √ | √ | 6/6 |  | √ | √ | √ | √ | 4/4 | E | |
| T16 - The French-Canadian version of the Fear-Avoidance Beliefs Questionnaire (FABQ) [29, 30] | MSD | √ | √ |  |  | √ | √ | 4/6 |  | √ | √ | √ | √ | 4/4 | E | |
| T17 - Two items (my work might harm my back; my work makes, or might make, my pain worse) from the Work subscale (7 items) of the Fear-Avoidance Beliefs Questionnaire (FABQ) [4, 6] | MSD | √ |  |  |  |  | √ | 2/6 |  | √ | √ | √ | √ | 4/4 | Q | |
| *All Coping strategies factors*: moderate evidence for MSDs |  |  |  |  |  |  |  |  |  |  |  |  |  |  |  | |
| T18 - Coping (cognitive strategies): Reinterpretations of pain sensations subscale (6 items) of the Coping Strategy Questionnaire [31-33] | MSD | √ |  | √ | √ | √ | √ | 5/6 |  | √ | √ | √ |  | 3/4 | E | |
| T19 - Coping (behavioural strategies): “Guarding” (9 items) subscale of the Chronic Pain Coping Inventory [32, 34, 35] | MSD | √ | √ | √ | √ |  | √ | 5/6 |  |  | √ | √ |  | 2/4 | E | |
| T20 - Coping (behavioural strategies): Increasing pain behaviour subscale (6 items) of the Coping Strategy Questionnaire [31-33] | MSD | √ |  |  |  | √ | √ | 3/6 |  | √ | √ | √ |  | 3/4 | G | |
| *Expectations (recovery)*: strong evidence for MSDs |  |  |  |  |  |  |  |  |  |  |  |  |  |  |  | |
| T21 - Three single items [36] | MSD | √ |  |  |  |  | √ | 2/6 |  | √ | √ | √ | √ | 4/4 | Q | |
| T22 - Single item: Belief that their back pain won’t disappear? [37] | MSD | √ |  |  |  |  | √ | 2/6 |  | √ | √ | √ | √ | 4/4 | Q | |
| T23 - Single item: When recover? [38] | MSD | √ |  |  |  |  | √ | 2/6 |  | √ | √ | √ | √ | 4/4 | Q | |
| T24 - Expectations of Recovery scale (7 items) [39] | MSD | √ |  |  |  |  | √ | 2/6 |  | √ | √ | √ | √ | 4/4 | Q | |
| T25 - Expectations of Recovery scale (6 items) [40] | MSD | √ |  |  |  |  | √ | 2/6 |  | √ | √ | √ | √ | 4/4 | Q | |
| T26 - Single item: In your view, how large is the risk that your current pain may become persistent? [41] ^c^ | MSD | √ |  |  |  |  | √ | 2/6 |  | √ | √ | √ | √ | 4/4 | Q | |
| *Locus of control*: strong evidence for MSDs |  |  |  |  |  |  |  |  |  |  |  |  |  |  |  | |
| T27 - Subscale Chance externality (6 items) of the Multidimensional Health Locus of Control questionnaire – Form A [42-44] | MSD | √ | √ | √ |  |  | √ | 4/6 |  | √ | √ | √ | √ | 4/4 | E | |
| T28 - Subscale Internality (6 items) of the Multidimensional Health Locus of Control questionnaire - Form A [42-44] | MSD | √ | √ | √ | √ |  | √ | 5/6 |  | √ | √ | √ | √ | 4/4 | E | |
| T29 - Subscale Internal locus of control (3 items) of the modified version of Wallston’s Health Locus of Control scale [45] | MSD | √ |  |  | √ | √ | √ | 4/6 |  | √ | √ | √ | √ | 4/4 | E | |
| *Job demands (physical)*: Moderate evidence for MSDs |  |  |  |  |  |  |  |  |  |  |  |  |  |  |  | |
| T30 - Dutch Musculoskeletal Questionnaire, 17 items (not clear in the predictive study which items among the 63 forming the DMQ) [46] | MSD | ? | ? | ? | ? | ? | √ | ? |  |  | ? | ? |  | ? | ? | |
| T31 - Three ad-hoc items about physical demands (working in twisted positions, working in the same position during a prolonged time, heavy lifts) [47] | MSD | √ |  |  |  |  | √ | 2/6 |  | √ | √ | √ | √ | 4/4 | Q | |
| T32 - One item from the Psychosocial Assessment Instrument [12] | MSD | √ |  |  |  |  | √ | 2/6 |  | √ | √ | √ | √ | 4/4 | Q | |
| T33 - Dutch Musculoskeletal Questionnaire, 7 items (manual material handling, frequent bending twisting of the trunk, whole body vibration, working in awkward postures, working in static postures, and strenuous work with neck/shoulder and the upper limb) [48] | MSD | ? | ? | ? | ? | ? | √ | ? |  | √ | ? | ? |  | ? | ? | |
| T34 - Four ad-hoc items about physical demands (repetitive movements, positions with constant strain on the back, hands above shoulder heights, and lifting more than 20 kg) [14] | MSD | √ |  |  |  |  | √ | 2/6 |  | √ | √ | √ | √ | 4/4 | Q | |
| T35 - Claim electronic file information based on the National Occupational Code (i.e., manual/high physical demands, mixed or non-manual work) [49] | MSD | √ |  |  |  |  | √ | 2/6 |  | √ |  | √ |  | 2/4 | Q | |
| *Job strain*: Moderate evidence for MSDs and for CMDs |  |  |  |  |  |  |  |  |  |  |  |  |  |  |  | |
| T36 - Two subscales of the Job Content Questionnaire (5 items for Job demands and 9 items for decision latitude) [29, 50-53] | MSD/CMD | √ | √ |  | √ | √ | √ | 5/6 |  |  | √ |  |  | 1/4 | G | |
| *Work ability:* Moderate evidence for MSDs |  |  |  |  |  |  |  |  |  |  |  |  |  |  |  | |
| T37 - Graded Reduced Work Ability scale (5 items) [54, 55] | MSD | √ | √ |  | √ |  | √ | 4/6 |  | √ | √ | √ | √ | 4/4 | E | |
| T38 - Single item (ability to perform the ordinary work) inspired by the Graded Reduced Work Ability scale [15, 56] | MSD | √ |  |  |  |  | √ | 2/6 |  | √ | √ | √ | √ | 4/4 | Q | |
| T39 - Single item from the Work ability index (current work ability compared with the lifetime best) [17, 57, 58] | MSD | √ |  |  |  |  | √ | 2/6 |  | √ | √ | √ | √ | 4/4 | Q | |
| *Self-efficacy (RTW):* Moderate evidence for MSDs |  |  |  |  |  |  |  |  |  |  |  |  |  |  |  | |
| T40 - Return-to-Work Obstacles and Self-Efficacy Scale (ROSES) [59] | MSD | √ | √ |  | √ | √ | √ | 5/6 |  |  | √ |  | √ | 2/4 | G | |
| T41 - Return to work self-efficacy questionnaire (11 items, RTW-SE) [60, 61] | MSD | √ | √ | √ | √ | √ | √ | 6/6 |  |  | √ |  | √ | 2/4 | G | |
| *Referred pain (back pain)*: moderate evidence for MSDs |  |  |  |  |  |  |  |  |  |  |  |  |  |  |  | |
| T42 - Single item: Where did you have pain last week? | MSD | √ |  |  |  |  | √ | 2/6 |  | √ | √ | √ | √ | 4/4 | Q | |
| T43 - Left and right leg typical sciatica from the physical examination | MSD |  |  |  |  |  |  | N.A. |  |  |  |  |  | N.A. |  | |
| T44 - Evidence of radiculopathy from the physical examination | MSD |  |  |  |  |  |  | N.A. |  |  |  |  |  | N.A. |  | |
| *Activities (disability/ODI)*: moderate evidence for MSDs |  |  |  |  |  |  |  |  |  |  |  |  |  |  |  | |
| T45 - Oswestry disability index (10 items) [62, 63] | MSD | √ | √ | √ | √ | √ | √ | 6/6 |  |  | √ |  | √ | 2/4 | E | |
| *Activities (disability/SF-36)*: moderate evidence for MSDs |  |  |  |  |  |  |  |  |  |  |  |  |  |  |  | |
| T46 - Physical functioning subscale (10 items) of the RAND-36 [64] | MSD | √ | √ | √ | √ | √ | √ | 6/6 |  |  | √ |  | √ | 2/4 | E | |
| T47 - Physical functioning subscale (10 items) of the SF-36 [64, 65] | MSD | √ | √ | √ | √ | √ | √ | 6/6 |  |  | √ |  |  | 1/4 | E | |
| *Catastrophizing (pain)*: moderate evidence for MSDs |  |  |  |  |  |  |  |  |  |  |  |  |  |  |  | |
| T48 - The Pain Catastrophizing Scale (13 items) [66, 67] | MSD | √ | √ | √ | √ | √ | √ | 6/6 |  |  | √ | √ | √ | 3/4 | E | |
| T49 - Mean of three items from the Pain Catastrophizing Scale [66, 67] ^c^ | MSD | √ |  |  |  |  | √ | 2/6 |  | √ | √ | √ | √ | 4/4 | Q | |
| *All fear factors*: moderate evidence for MSDs |  |  |  |  |  |  |  |  |  |  |  |  |  |  |  | |
| T50 - Fear of relapse subscale (4 Items) of the ROSES [59] | MSD | √ | √ |  | √ | √ | √ | 5/6 |  | √ | √ | √ | √ | 4/4 | E | |
| T51 - Single item: *It is not advisable to be physically active* [24, 68] ^c^ | MSD | √ |  |  |  |  | √ | 2/6 |  | √ | √ | √ | √ | 4/4 | Q | |
| T52 - Single item: *If you continue working, what effect will that have on your complaints?* | MSD | √ |  |  |  |  | √ | 2/6 |  | √ | √ | √ | √ | 4/4 | Q | |
| T53 - Work subscale (7 items) of the FABQ [23, 24] | MSD | √ | √ | √ | √ | √ | √ | 6/6 |  | √ | √ | √ | √ | 4/4 | E | |
| *Illness behaviours*: moderate evidence for MSDs |  |  |  |  |  |  |  |  |  |  |  |  |  |  |  | |
| T54 - Two simulation tests from the Waddell’s nonorganic signs [69, 70] | MSD | √ | √ | √ |  |  | √ | 4/6 |  | √ | √ | √ | √ | 4/4 | E | |
| T55 - Guarding pain behaviour, from the pain behaviour observation system [71, 72] | MSD | √ | √ | √ | √ |  | √ | 5/6 |  |  |  |  |  | 0/4 | G | |
| T56 - Waddell’s symptoms [70] | MSD | √ | √ | √ |  |  | √ | 4/6 |  | √ | √ | √ | √ | 4/4 | E | |
| *Mental vitality*: moderate evidence for MSDs |  |  |  |  |  |  |  |  |  |  |  |  |  |  |  | |
| T57 - Vitality subscale (4 items) of the SF-36 V1 [64, 65] | MSD | √ | √ | √ | √ | √ | √ | 6/6 |  | √ | √ |  | √ | 3/4 | E | |
| *Positive health change*: moderate evidence for MSDs |  |  |  |  |  |  |  |  |  |  |  |  |  |  |  | |
| T58 - Single item from RAND-36 or SF-36-v1: Compared to one year ago, how would you rate your health in general now? ^c^ | MSD | √ |  |  |  |  | √ | 2/6 |  | √ | √ | √ | √ | 4/4 | Q | |
| *Sleep quality*: moderate evidence for MSDs and CMDs |  |  |  |  |  |  |  |  |  |  |  |  |  |  |  | |
| T59 - Insomnia Severity Index (7 items) [73, 74] | MSD/  CMD | √ | √ | √ | √ |  | √ | 5/6 |  | √ | √ | √ | √ | 4/4 | E | |
| T60 - Single item: I can sleep at night. [41] ^c^ | MSD | √ |  |  |  |  | √ | 2/6 |  | √ | √ |  | √ | 3/4 | Q | |
| T66 - Single item: Subjective experience of reduced duration or depth of sleep compared with the subject’s normal pattern when well. [75] c | CMD | √ |  |  |  |  | √ | 2/6 |  | √ | √ | √ | √ | 4/4 | Q | |
| *Job demands (psychological):* Moderate evidence for CMDs |  |  |  |  |  |  |  |  |  |  |  |  |  |  |  | |
| T65 - Subscale of the Job Content Questionnaire (psychological demands, 5 items) [50, 51, 53] | CMD | √ | √ |  | √ | √ | √ | 5/6 |  | √ | √ | √ |  | 3/4 | E | |
| *All participation factors*: moderate evidence for CMDs |  |  |  |  |  |  |  |  |  |  |  |  |  |  |  | |
| T67 - Social functioning subscale (2 items) of the Swedish SF-36-v2 [76, 77] | CMD | √ | √ | √ |  | √ | √ | 5/6 |  | √ | √ | √ |  | 3/4 | E | |
| T68 - Social and Occupational Functioning Assessment Scale [78, 79] | CMD | √ | √ | √ |  |  | √ | 4/6 |  | √ | √ | √ |  | 3/4 | E | |

^a^ Some usability information is provided in the **S3 file**

^b^ E: Excellent; G: Good; Q: Questionable; according to **Table 2**.

^c^ The reported psychometric properties correspond to a single item (or shorter scale) are not as good (usually unknown) as reported for the whole questionnaire or subscale for which references are provided.

N.A.: not applicable

# **References**

1. Hogg-Johnson S, Cole DC. Early prognostic factors for duration on temporary total benefits in the first year among workers with compensated occupational soft tissue injuries. Occup Environ Med. 2003;60(4):244-53. Epub 2003/03/28. doi: 10.1136/oem.60.4.244. PubMed PMID: 12660372; PubMed Central PMCID: PMC1740514.

2. Franche R-L, Severin CN, Hogg-Johnson S, Côté P, Vidmar M, Lee H. The impact of early workplace-based return-to-work strategies on work absence duration: a 6-month longitudinal study following an occupational musculoskeletal injury. Journal of occupational and environmental medicine. 2007;49(9):960-74. doi: 10.1097/jom.0b013e31814b2e9f. PubMed PMID: 17848852.

3. Iles RA, Sheehan LR, Gosling CM. Assessment of a new tool to improve case manager identification of delayed return to work in the first two weeks of a workers' compensation claim. Clin Rehabil. 2020;34(5):656-66. Epub 2020/03/19. doi: 10.1177/0269215520911417. PubMed PMID: 32183561.

4. Turner JA, Franklin G, Fulton-Kehoe D, Sheppard L, Stover B, Wu R, et al. ISSLS prize winner: early predictors of chronic work disability: a prospective, population-based study of workers with back injuries. Spine (Phila Pa 1976). 2008;33(25):2809-18. Epub 2008/12/04. doi: 10.1097/BRS.0b013e31817df7a7. PubMed PMID: 19050587.

5. Beemster TT, van Bennekom CAM, van Velzen JM, Frings-Dresen MHW, Reneman MF. Vocational Rehabilitation with or without Work Module for Patients with Chronic Musculoskeletal Pain and Sick Leave from Work: Longitudinal Impact on Work Participation. J Occup Rehabil. 2021;31(1):72-83. Epub 2020/05/08. doi: 10.1007/s10926-020-09893-z. PubMed PMID: 32378023; PubMed Central PMCID: PMC7954725.

6. Turner JA, Franklin G, Fulton-Kehoe D, Sheppard L, Wickizer TM, Wu R, et al. Worker recovery expectations and fear-avoidance predict work disability in a population-based workers' compensation back pain sample. Spine (Phila Pa 1976). 2006;31(6):682-9. Epub 2006/03/17. doi: 10.1097/01.brs.0000202762.88787.af. PubMed PMID: 16540874.

7. Du Bois M, Donceel P. A screening questionnaire to predict no return to work within 3 months for low back pain claimants. Eur Spine J. 2008;17(3):380-5. Epub 2008/01/04. doi: 10.1007/s00586-007-0567-8. PubMed PMID: 18172698; PubMed Central PMCID: PMC2270393.

8. Du Bois M, Szpalski M, Donceel P. Patients at risk for long-term sick leave because of low back pain. Spine J. 2009;9(5):350-9. doi: 10.1016/j.spinee.2008.07.003. PubMed PMID: 18790677.

9. Fishbain DA, Cutler RB, Rosomoff HL, Khalil T, Steele-Rosomoff R. Impact of chronic pain patients' job perception variables on actual return to work. Clin J Pain. 1997;13(3):197-206. Epub 1997/09/26. doi: 10.1097/00002508-199709000-00004. PubMed PMID: 9303251.

10. Gross DP, Battié MC. Factors Influencing Results of Functional Capacity Evaluations in Workers' Compensation Claimants With Low Back Pain. Physical Therapy. 2005;85(4):315-22. doi: 10.1093/ptj/85.4.315.

11. Gross DP, Battié MC. Recovery expectations predict recovery in workers with back pain but not other musculoskeletal conditions. J Spinal Disord Tech. 2010;23(7):451-6. Epub 2010/04/24. doi: 10.1097/BSD.0b013e3181d1e633. PubMed PMID: 20414134.

12. Hara KW, Bjørngaard JH, Jacobsen HB, Borchgrevink PC, Johnsen R, Stiles TC, et al. Biopsychosocial predictors and trajectories of work participation after transdiagnostic occupational rehabilitation of participants with mental and somatic disorders: a cohort study. BMC Public Health. 2018;18(1):1014. doi: 10.1186/s12889-018-5803-0.

13. Nicholas MK, Costa DSJ, Linton SJ, Main CJ, Shaw WS, Pearce R, et al. Predicting Return to Work in a Heterogeneous Sample of Recently Injured Workers Using the Brief ÖMPSQ-SF. J Occup Rehabil. 2019;29(2):295-302. Epub 2018/05/26. doi: 10.1007/s10926-018-9784-8. PubMed PMID: 29796980.

14. Reme SE, Hagen EM, Eriksen HR. Expectations, perceptions, and physiotherapy predict prolonged sick leave in subacute low back pain. BMC Musculoskelet Disord. 2009;10:139. Epub 2009/11/17. doi: 10.1186/1471-2474-10-139. PubMed PMID: 19912626; PubMed Central PMCID: PMC2780378.

15. Sampere M, Gimeno D, Serra C, Plana M, López JC, Martínez JM, et al. Return to work expectations of workers on long-term non-work-related sick leave. J Occup Rehabil. 2012;22(1):15-26. Epub 2011/06/28. doi: 10.1007/s10926-011-9313-5. PubMed PMID: 21701951.

16. Steenstra IA, Koopman FS, Knol DL, Kat E, Bongers PM, de Vet HC, et al. Prognostic factors for duration of sick leave due to low-back pain in dutch health care professionals. J Occup Rehabil. 2005;15(4):591-605. doi: 10.1007/s10926-005-8037-9. PubMed PMID: 16254758.

17. Wåhlin C, Ekberg K, Persson J, Bernfort L, Oberg B. Association between clinical and work-related interventions and return-to-work for patients with musculoskeletal or mental disorders. J Rehabil Med. 2012;44(4):355-62. Epub 2012/03/22. doi: 10.2340/16501977-0951. PubMed PMID: 22434378.

18. Løvvik C, Shaw W, Overland S, Reme SE. Expectations and illness perceptions as predictors of benefit recipiency among workers with common mental disorders: secondary analysis from a randomised controlled trial. BMJ Open. 2014;4(3):e004321. Epub 2014/03/05. doi: 10.1136/bmjopen-2013-004321. PubMed PMID: 24589824; PubMed Central PMCID: PMC3948454.

19. Nielsen MB, Madsen IE, Bultmann U, Christensen U, Diderichsen F, Rugulies R. Predictors of return to work in employees sick-listed with mental health problems: findings from a longitudinal study. Eur J Public Health. 2011;21(6):806-11. Epub 2010/12/04. doi: 10.1093/eurpub/ckq171. PubMed PMID: 21126986.

20. Nieuwenhuijsen K, Verbeek JH, de Boer AG, Blonk RW, van Dijk FJ. Predicting the duration of sickness absence for patients with common mental disorders in occupational health care. Scand J Work Environ Health. 2006;32(1):67-74. PubMed PMID: 16539174.

21. Hedlund Å, Nilsson A, Boman E, Kristofferzon ML. Predictors of return to work and psychological well-being among women during/after long-term sick leave due to common mental disorders - a prospective cohort study based on the theory of planned behaviour. Health Soc Care Community. 2022;30(6):e5245-e58. Epub 2022/07/28. doi: 10.1111/hsc.13943. PubMed PMID: 35894151; PubMed Central PMCID: PMC10087653.

22. Hedlund Å, Kristofferzon ML, Boman E, Nilsson A. Are return to work beliefs, psychological well-being and perceived health related to return-to-work intentions among women on long-term sick leave for common mental disorders? A cross-sectional study based on the theory of planned behaviour. BMC Public Health. 2021;21(1):535. Epub 2021/03/21. doi: 10.1186/s12889-021-10562-w. PubMed PMID: 33740921; PubMed Central PMCID: PMC7977300.

23. Waddell G, Newton M, Henderson I, Somerville D, Main CJ. A fear-avoidance beliefs questionnaire (FABQ) and the role of fear-avoidance beliefs in chronic low back pain and disability. Pain. 1993;52:157-68.

24. Lundberg M, Grimby-Ekman A, Verbunt J, Simmonds MJ. Pain-related fear: a critical review of the related measures. Pain Res Treat. 2011;2011:494196. doi: 10.1155/2011/494196. PubMed PMID: 22191022; PubMed Central PMCID: PMCPMC3236324.

25. Grøvle L, Haugen AJ, Keller A, Ntvig B, Brox JI, Grotle M. Prognostic factors for return to work in patients with sciatica. Spine J. 2013;13(12):1849-57. Epub 2013/09/26. doi: 10.1016/j.spinee.2013.07.433. PubMed PMID: 24060231.

26. Opsahl J, Eriksen HR, Tveito TH. Do expectancies of return to work and Job satisfaction predict actual return to work in workers with long lasting LBP? BMC Musculoskelet Disord. 2016;17(1):481. Epub 2016/11/20. doi: 10.1186/s12891-016-1314-2. PubMed PMID: 27855684; PubMed Central PMCID: PMC5114779.

27. Oyeflaten I, Hysing M, Eriksen HR. Prognostic factors associated with return to work following multidisciplinary vocational rehabilitation. J Rehabil Med. 2008;40(7):548-54. doi: 10.2340/16501977-0202. PubMed PMID: 18758672.

28. Storheim K, Brox, J. I., Holm, I., & Bo, K. . Predictors of return to work in patients sick listed for sub-acute low back pain: a 12-month follow-up study. Journal of rehabilitation medicine. 2005;37(6):365-71. doi: <https://doi.org/10.1080/16501970510040344>.

29. Soucy I, Truchon M, Côté D. Work-related factors contributing to chronic disability in low back pain. Work. 2006;26(3):313-26. Epub 2006/05/25. PubMed PMID: 16720972.

30. Chaory K, Fayad F, Rannou F, Lefèvre-Colau MM, Fermanian J, Revel M, et al. Validation of the French version of the fear avoidance belief questionnaire. Spine (Phila Pa 1976). 2004;29(8):908-13. Epub 2004/04/15. doi: 10.1097/00007632-200404150-00018. PubMed PMID: 15082995.

31. Rosenstiel AK, Keefe FJ. The use of coping strategies in chronic low back pain patients: relationship to patient characteristics and current adjustment. Pain. 1983;17(1):33-44. PubMed PMID: 6226916.

32. Banerjee A, Hendrick P, Bhattacharjee P, Blake H. A systematic review of outcome measures utilised to assess self-management in clinical trials in patients with chronic pain. Patient education and counseling. 2018;101(5):767-78.

33. Abbott A. The Coping Strategy Questionnaire. Journal of physiotherapy. 2010;56(1). doi: 10.1016/s1836-9553(10)70061-8.

34. Jensen MP, Turner JA, Romano JM, Strom SE. The Chronic Pain Coping Inventory: development and preliminary validation. Pain. 1995;60(2):203-16.

35. Truchon M, Cote D, Irachabal S. The Chronic Pain Coping Inventory: confirmatory factor analysis of the French version. BMC musculoskeletal disorders. 2006;7:13. doi: 10.1186/1471-2474-7-13. PubMed PMID: 16478541; PubMed Central PMCID: PMCPMC1386669.

36. Cole DC, Mondloch MV, Hogg-Johnson S. Listening to injured workers: how recovery expectations predict outcomes--a prospective study. CMAJ. 2002;166(6):749-54.

37. Hagen EM, Svensen E, Eriksen HR. Predictors and modifiers of treatment effect influencing sick leave in subacute low back pain patients. Spine (Phila Pa 1976). 2005;30(24):2717-23. PubMed PMID: 16371893.

38. Hogg-Johnson S, Cole D. Early prognostic factors for duration on temporary total benefits in the first year among workers with compensated occupational soft tissue injuries. Occupational and Environmental Medicine. 2003;60(4):244-53.

39. Schultz IZ, Crook J, Berkowitz J, Milner R, Meloche GR. Predicting Return to Work After Low Back Injury Using the Psychosocial Risk for Occupational Disability Instrument: A Validation Study. Journal of Occupational Rehabilitation. 2005;15(3):365-76. doi: 10.1007/s10926-005-5943-9.

40. Schultz I, Z., Crook J, Meloche GR, Berkowitz J, Milner R, Zuberbier OA, et al. Psychosocial factors predictive of occupational low back disability: towards development of a return-to-work model. Pain. 2004;107(1-2):77-85.

41. Linton SJ, Nicholas M, Macdonald S. Development of a short form of the orebro musculoskeletal pain screening questionnaire. Spine (Phila Pa 1976). 2011;36(22):1891-5.

42. Wallston KA, Wallston BS, DeVellis R. Development of the Multidimensional Health Locus of Control (MHLC) Scales. Health Educ Monogr. 1978;6(2):160-70. doi: 10.1177/109019817800600107. PubMed PMID: 689890.

43. Wallston KA. The validity of the multidimensional health locus of control scales. J Health Psychol. 2005;10(5):623-31. doi: 10.1177/1359105305055304. PubMed PMID: 16033784.

44. Ross TP, Ross LT, Short SD, Cataldo S. The Multidimensional Health Locus of Control Scale: Psychometric Properties and Form Equivalence. Psychol Rep. 2015;116(3):889-913. Epub 2015/05/12. doi: 10.2466/09.02.PR0.116k29w3. PubMed PMID: 25961714.

45. Murphy GC, Young AE, Vo K-M. Using locus of control to predict the return-to-work achievements of back-injured occupational rehabilitation clients. The Australian Journal of Rehabilitation Counselling. 1995;1(2):83-92.

46. Bosman LC, Twisk JWR, Geraedts AS, Heymans MW. Development of Prediction Model for the Prognosis of Sick Leave Due to Low Back Pain. J Occup Environ Med. 2019;61(12):1065-71. Epub 2019/10/28. doi: 10.1097/jom.0000000000001749. PubMed PMID: 31651601.

47. Hansson TH, Hansson EK. The effects of common medical interventions on pain, back function, and work resumption in patients with chronic low back pain: A prospective 2-year cohort study in six countries. Spine (Phila Pa 1976). 2000;25(23):3055-64. Epub 2001/01/06. doi: 10.1097/00007632-200012010-00013. PubMed PMID: 11145817.

48. Lötters F, Burdorf A. Prognostic factors for duration of sickness absence due to musculoskeletal disorders. Clin J Pain. 2006;22(2):212-21. Epub 2006/01/24. doi: 10.1097/01.ajp.0000154047.30155.72. PubMed PMID: 16428958.

49. Steenstra IA, Busse JW, Tolusso D, Davilmar A, Lee H, Furlan AD, et al. Predicting time on prolonged benefits for injured workers with acute back pain. J Occup Rehabil. 2015;25(2):267-78. Epub 2014/08/29. doi: 10.1007/s10926-014-9534-5. PubMed PMID: 25164779; PubMed Central PMCID: PMC4436678.

50. Haveraaen LA, Skarpaas LS, Aas RW. Job demands and decision control predicted return to work: the rapid-RTW cohort study. BMC Public Health. 2017;17(1):154. doi: 10.1186/s12889-016-3942-8.

51. Haveraaen LA, Skarpaas LS, Berg JE, Aas RW. Do psychological job demands, decision control and social support predictreturn to work three months after a return-to-work (RTW) programme? The rapid-RTW cohort study. Work. 2015;53 1:61-71.

52. Karasek R, Theorell Tr. Healthy work : stress, productivity, and the reconstruction of working life. New York: Basic Books New York; 1990.

53. Karasek R, Brisson C, Kawakami N, Houtman I, Bongers P, Amick B. The Job Content Questionnaire (JCQ): an instrument for internationally comparative assessments of psychosocial job characteristics. J Occup Health Psychol. 1998;3(4):322-55. Epub 1998/11/07. doi: 10.1037//1076-8998.3.4.322. PubMed PMID: 9805280.

54. Haldorsen EM, Indahl A, Ursin H. Patients with low back pain not returning to work. A 12-month follow-up study. Spine (Phila Pa 1976). 1998;23(11):1202-7; discussion 8. Epub 1998/06/24. doi: 10.1097/00007632-199806010-00004. PubMed PMID: 9636972.

55. Coole C. Changing perceptions of work ability in people with low back pain: a feasibility and economic evaluation.: University of Nottingham; 2012.

56. Reiso H, Nygård JF, Brage S, Gulbrandsen P, Tellnes G. Work ability and duration of certified sickness absence. Scand J Public Health. 2001;29(3):218-25. Epub 2001/10/30. PubMed PMID: 11680774.

57. Tuomi K, Ilmarinen J, Jahkola A, Katajarinne L, Tulkki A. Work ability index: Finnish Institute of Occupational Health Helsinki; 1998.

58. Ilmarinen J. The Work Ability Index (WAI). Occupational Medicine. 2007;57(2):160-. doi: 10.1093/occmed/kqm008.

59. Corbiere M, Negrini A, Durand MJ, St-Arnaud L, Briand C, Fassier JB, et al. Development of the Return-to-Work Obstacles and Self-Efficacy Scale (ROSES) and Validation with Workers Suffering from a Common Mental Disorder or Musculoskeletal Disorder. J Occup Rehabil. 2017;27(3):329-41. doi: 10.1007/s10926-016-9661-2. PubMed PMID: 27562583.

60. Huijs JJ, Koppes LL, Taris TW, Blonk RW. Differences in predictors of return to work among long-term sick-listed employees with different self-reported reasons for sick leave. J Occup Rehabil. 2012;22(3):301-11. Epub 2012/02/04. doi: 10.1007/s10926-011-9351-z. PubMed PMID: 22302668.

61. Lagerveld SE, Blonk RWB, Brenninkmeijer V, Schaufeli WB. Return to work among employees with mental health problems: Development and validation of a self-efficacy questionnaire. Work & Stress. 2010;24(4):359-75. doi: 10.1080/02678373.2010.532644.

62. Fairbank JCT, Couper J, Davies J, O'Brien J. The Oswestry low back pain disability questionnaire. Physiotherapy. 1980;66(8):271-3.

63. Fairbank JCT, Pynsent PB. The Oswestry disability index. Spine. 2000;25(22):2940-53.

64. Ware JE, Snow KK, Kosinski M, Gandek B. SF-36 Health survey : manual and interpretation guide. Boston: Health Institute, New England Medical Center; 1993 1993.

65. Ware Jr JE. SF-36 health survey update. Spine. 2000;25(24):3130-9.

66. Sullivan MJL, Bishop SR, Pivik J. The Pain Catastrophizing Scale: Development and validation. Psychological Assessment. 1995;7:524-32.

67. Wheeler CHB, Williams ACC, Morley SJ. Meta-analysis of the psychometric properties of the Pain Catastrophizing Scale and associations with participant characteristics. Pain. 2019;160(9):1946-53. Epub 2019/01/30. doi: 10.1097/j.pain.0000000000001494. PubMed PMID: 30694929.

68. Vlaeyen JWS, Kole-Snijders AMJ, Boeren RGB, van Eek H. Fear of movement / (re)injury in chronic low back pain and its relation to behavioral performance. Pain. 1995;62(3):363-72.

69. Waddell G, McCulloch JA, Kummel E, Venner RM. Nonorganic physical signs in low-back pain. Spine. 1980;5(2):117-25.

70. Waddell G, Main CJ, Morris EW, Di Paola M, Gray IC. Chronic low-back pain, psychologic distress, and illness behavior. Spine. 1984;9(2):209-13.

71. Prkachin KM, Hughes E, Schultz I, Joy P, Hunt D. Real-time assessment of pain behavior during clinical assessment of low back pain patients. Pain. 2002;95(1-2):23-30.

72. Prkachin KM, Schultz I, Berkowitz J, Hughes E, Hunt D. Assessing pain behaviour of low-back pain patients in real time: concurrent validity and examiner sensitivity. BehavResTher. 2002;40(5):595-607.

73. Morin CM, Belleville G, Bélanger L, Ivers H. The Insomnia Severity Index: psychometric indicators to detect insomnia cases and evaluate treatment response. Sleep. 2011;34(5):601-8.

74. Bastien CH, Vallières A, Morin CM. Validation of the Insomnia Severity Index as an outcome measure for insomnia research. Sleep Medicine. 2001;2(4):297-307.

75. Svanborg P, Åsberg M. A new self‐rating scale for depression and anxiety states based on the Comprehensive Psychopathological Rating Scale. Acta Psychiatrica Scandinavica. 1994;89(1):21-8.

76. Sullivan M, Karlsson J. The Swedish SF-36 Health Survey III. Evaluation of criterion-based validity: results from normative population. J Clin Epidemiol. 1998;51(11):1105-13. doi: 10.1016/s0895-4356(98)00102-4. PubMed PMID: 9817128.

77. Ware JE, Gandek B. Overview of the SF-36 Health Survey and the International Quality of Life Assessment (IQOLA) Project. Journal of Clinical Epidemiology. 1998;51(11):903-12. doi: 10.1016/s0895-4356(98)00081-x.

78. Hilsenroth MJ, Ackerman SJ, Blagys MD, Baumann BD, Baity MR, Smith SR, et al. Reliability and validity of DSM-IV axis V. Am J Psychiatry. 2000;157(11):1858-63. doi: 10.1176/appi.ajp.157.11.1858. PubMed PMID: 11058486.

79. Goldman HH, Skodol AE, Lave TR. Revising axis V for DSM-IV: a review of measures of social functioning. Am J Psychiatry. 1992;149(9):1148-56. doi: 10.1176/ajp.149.9.1148. PubMed PMID: 1386964.
